# Effect of Thionation of the Carbonyl Groups in Naphthalimide‐Phenoxazine Electron Donor–Acceptor Dyads on the Excited‐ State Dynamics: Transient Optical and Electron Paramagnetic Resonance Spectral Studies

**DOI:** 10.1002/chem.202502885

**Published:** 2025-11-10

**Authors:** Yuqiong Zhang, Xue Zhang, Shihui He, Sveva Linarello, Antonio Toffoletti, Jianzhang Zhao, Yan Wan, Antonio Barbon

**Affiliations:** ^1^ State Key Laboratory of Fine Chemicals Frontier Science Center for Smart Materials School of Chemical Engineering Dalian University of Technology 2 Ling Gong Rd. Dalian 116024 P. R. China; ^2^ College of Chemistry Beijing Normal University Beijing 100875 P. R. China; ^3^ Dipartimento di Scienze Chimiche Università degli Studi di Padova Via Marzolo, 1 Padova 35131 Italy

**Keywords:** charge separation, intersystem crossing, thionation, time‐resolved electron paramagnetic resonance, triplet photosensitizer

## Abstract

We performed thionation of the electron acceptor naphthalimide (NI) moiety in donor–acceptor (D‐A) dyads in a series of thio‐substituted D‐A dyads. Thionation significantly enhances the electron‐accepting ability of the chromophore, facilitating the formation of low‐lying charge‐separated (CS) states. Femtosecond transient absorption spectra revealed intersystem crossing (ISC) rate constants of **S‐NI‐PXZ** (*k*_ISC_ = 3.8 × 10^11^ s^−1^) and **DS‐NI‐PXZ** (*k*_ISC_ = 2.4 × 10^11^ s^−1^), which are interestingly independent of the number of sulfur atoms. Generally, fluorescence quenching was observed due to the rapid ISC process. Native **NI‐PXZ** exhibits thermally activated delayed fluorescence (TADF). Thionation increased the singlet‐triplet energy gap, making the TADF vanish. In *n*‐hexane, nanosecond transient absorption spectroscopy detected the triplet lifetimes shortened from 11.6 µs of **NI‐PXZ** to 0.66 µs for **S‐NI‐PXZ** and 0.25 µs for **DS‐NI‐PXZ**. Time‐resolved electron paramagnetic resonance revealed the large zero‐field splitting *D* value (4200–9800 MHz) of the triplet state of the thionated derivatives, which is due to the much larger SOC matrix elements of triplet state of **DS‐NI‐PXZ** (ca. 135 cm^−1^) and **DS‐NI** (ca. 136 cm^−1^) than the nonthionated **NI** (3.1 cm^−1^). Thionation of the carbonyl groups in chromophore is a promising method for design of triplet photosensitizers to achieve fast ISC.

## Graphical abstract

Thionation: Enhanced SOC and Unusually Large ZFS Parameters: Thionated electron donor–acceptor dyads based on naphthalimide (NI) and phenoxazine (PXZ) were prepared and the photophysical properties were studied. Fluorescence quenching and efficient intersystem crossing (ISC) were observed for the dyad with the NI unit thionated. Large zero field splitting *D* value of the triplet state indicates that the enhanced spin‐orbit coupling effect by the thionated carbonyls in the chromophore.

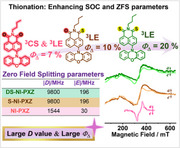

## 1 Introduction

Mimicking the natural photosynthesis center by formation of long‐lived charge‐separated (CS) states in electron donor − acceptor dyads has attracted much attention for decades.^[^ ^1^, ^2^, ^3^, ^4^, ^5^, ^6^, ^7^, ^8^, ^9^ ^]^ Long‐lived CS state is critical in solar cells,^[^ ^10^, ^11^, ^12^, ^13^ ^]^ photocatalysis,^[^ ^14^, ^15^, ^16^, ^17^, ^18^ ^]^ etc. Marcus theory of electron transfer (Equation 1) can be used to guide the molecular structure design to obtain long‐lived CS state,^[^ ^19^, ^20^ ^]^

(1) $$k_{\mathrm{ET}}=\left(\frac{4\pi^{3}}{h^{2}\lambda k_{B}T}\right)V^{2}\exp\left[-\frac{{\left(\Delta G_{\mathrm{ET}}^{0}+\lambda \right)}^{2}}{4\lambda k_{B}T}\right]$$

where *h* and *k*_B_ represent the Planck and Boltzmann constants, respectively, $\Delta G_{\mathrm{ET}}^{0}$ is the Gibbs free energy change of the electron transfer, *T* is temperature, *V* is the electronic matrix element, and *λ* is the total reorganization energy. For instance, the Marcus inverted region effect can be used to prolong the CS state lifetime, by pushing the CS state energy higher (mainly by using of electron donor and acceptor with proper redox potentials).^[^ ^20^, ^21^, ^22^ ^]^ These dyads be designed to show absorption in the UV or blue spectral range, clearly are not suitable for applications where visible light source is used, for instance, solar light, in solar cell, or photocatalytic redox organic reactions, etc. By using electron donor and acceptor show small reorganization energy (*λ*), With small *λ* values, the charge recombination (CR) process easily goes into the Marcus inverted region of electron transfer.^[^ ^2^, ^23^ ^]^ Moreover, reducing the electronic coupling matrix elements (*V*) can also slow down the CR process.^[^ ^24^ ^]^ Based on these strategies, electron donor − acceptor dyads showing long‐lived CS states have been successfully developed. However, the molecular structures based on these strategies are usually complicated, and the synthesis is challenging.^[^ ^22^, ^25^, ^26^, ^27^, ^28^ ^]^

Recently, another method to prolong the CS state lifetimes has been proposed, that is, the electron spin control approach. With this method, a triplet CS state, that is, ^3^CS state, is formed upon photoexcitation of the electron donor − acceptor dyad, instead of the singlet CS state, that is, ^1^CS state, formed for most of the conventional electron donor − acceptor dyads. Because the CS and CR processes are both electron spin‐selective, both the intersystem crossing (ISC), ^3^CS→^1^CS and the CR of ^3^CS→S_0_ are electron spin forbidden, whereas the CR of ^1^CS→S_0_ is electron spin allowed, thus, ^3^CS state should be longer‐lived than the ^1^CS state.^[^ ^26^, ^29^, ^30^, ^31^, ^32^ ^]^ This approach has been successfully confirmed by a few electron donor − acceptor dyads. However, it is not a trivial task to implement this strategy to obtain long lived CS state. First, the coupling between the electron donor and acceptor must be strong enough, so that the electron exchange (*J*) is large enough. As a result, a stable ^1^CS state with respect to ^3^CS state can be formed. In the case of small *J* value, a spin‐correlated radical pair (SCRP) or a free radical pair will be resulted from the electron transfer process, activating deactivation processes which reduce the CS state lifetime.^[^ ^3^ ^]^ Second, since the CS process is also electron spin selective a triplet precursor is required to produce the ^3^CS state, such as a singlet localized excited state. Thus, a chromophore showing fast ISC is required to compete with fast electron transfer. Transition metal complex molecular structure motifs have been used to achieve this goal.^[^ ^31^, ^33^, ^34^ ^]^ Recently we proposed to use orthogonal electron donor − acceptor dyads to achieve the electron spin control effect to prolong the CS state lifetime,^[^ ^35^, ^36^, ^37^ ^]^ especially the electron donor–acceptor dyads showing the thermally activated delayed fluorescence (TADF) property.^[^ ^38^, ^39^, ^40^, ^41^, ^42^, ^43^, ^44^ ^]^ Usually, a short linker is used between the donor and acceptor. However, such examples are rare; more molecular structure motifs need to be exploited.

Concerning orthogonality, electron donor − acceptor dyads showing TADF is of particular interest.^[^ ^45^, ^46^, ^47^, ^48^, ^49^, ^50^, ^51^ ^]^ There are three states showing similar energy for these dyads, that is, the emissive ^1^CS state (note that the coupling between the donor and acceptor is strong for these dyads; thus, the ^1^CS state usually relax through a radiative path) and the ^3^LE and ^3^CS state are dark states. Upon photoexcitation, ^1^LE/^1^CS states are produced firstly, then via spin‐orbit charge transfer ISC (SOCT‐ISC), the locally excited triplet (^3^LE) state is produced, the secondary CS process of ^3^LE→^3^CS produce the ^3^CS state.^[^ ^45^, ^52^, ^53^, ^54^, ^55^ ^]^ We found the ^3^CS state has a long lifetime (∼ µs) with nanosecond transient absorption (ns‐TA) spectroscopy, whereas the ^1^CS state is short‐lived (∼ ns).^[^ ^42^, ^56^, ^57^ ^]^

A detailed photophysical study on the electron donor − acceptor dyads showing ^3^CS state is rare. The fluorescence spectroscopy only characterize the emissive S_1_ state.^[^ ^58^ ^]^ Transient absorption spectroscopy (on femtosecond and nanosecond time scale) characterize the formation process of the states,^[^ ^59^, ^60^, ^61^ ^]^ and the lifetimes of ^3^LE and ^3^CS state. However, it is not a trivial task to discriminate the ^1^CS state and ^3^CS state with transient optical absorption spectroscopy,^[^ ^41^ ^−^ ^43^ ^]^ because the difference of the two states is only the electron spin angular momentum, not the electronic transitions (energy gap, molecular orbital occupancy, and transition probability of the electronic states), this is different from the scenario of the different character of the excited state absorption (ESA) of S_1_ state and T_1_ state. To this end, the time‐resolved electron paramagnetic resonance (TREPR) spectroscopy has been used to characterize the electron donor–acceptor TADF dyads. Using this technique, the ^3^CS state was observed, as well as the ^3^LE state.^[^ ^62^, ^63^, ^64^, ^65^ ^]^ We also used the TREPR spectroscopy to confirm that the ^3^LE state is produced via the CR mechanism, not the ordinary SOC effect, based on the electron spin polarization pattern of the ^3^LE state TREPR spectra.^[^ ^38^, ^39^, ^41^, ^57^, ^66^ ^]^ In addition, the triplet state signal and the zero‐field splitting (ZFS) parameters |*D*| and |*E*| determined with TREPR spectra reveal the electron spin multiplicity of the CS state and distinguish the character of n–π^∗^ and π–π^∗^.^[^ ^67^ ^]^ However, more molecular systems are needed to fully unveil the photophysics of these TADF emitters.

Recently, we prepared naphthalimide (NI)‐phenothiazine (PTZ) and phenoxazine (PXZ) dyads as model TADF emitters.^[^ ^38^, ^41^ ^]^ We used femtosecond and nanosecond transient absorption spectroscopy, as well as TREPR to study the photophysics of these compact electron donor‐acceptor dyads. We also tuned the photophysics of the dyads by changing the redox potentials of the donor and acceptor, or the distance between the donor and the acceptor.^[^ ^44^, ^68^ ^]^ In order to fully unveil the photophysical property of the dyads, it is critical to develop a novel approach to tune one specific property of the TADF emitters, while keep other properties intact.^[^ ^56^, ^64^, ^68^, ^69^ ^]^ To the best of our knowledge, such a strategy was rarely reported for the study of the photophysical property of the electron donor–acceptor dyads.

**Scheme 1:**
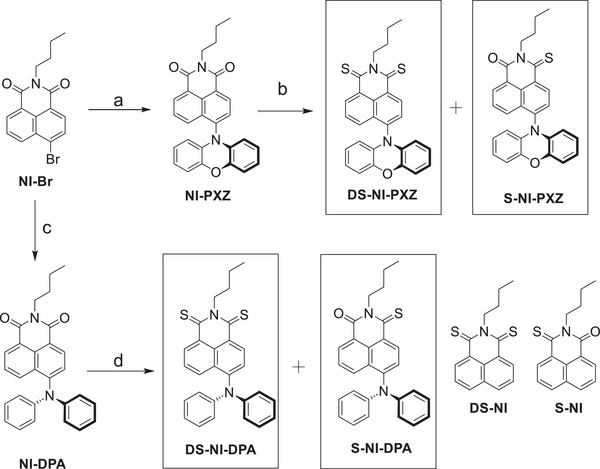
Synthesis of the target compounds. a) Phenoxazine, Pd(OAc)_2_, P(*t*‐Bu)_3_HBF_4_, *t*‐BuONa, dry toluene (TOL), reflux, 10 hours, yield: 48%; b) Lawesson's reagent, dry TOL, 110 °C, N_2_, reflux, 18 hours, yield: 24% (**DS‐NI‐PXZ**) and 20% (**S‐NI‐PXZ**), respectively; c) Diphenylamine, Pd(OAc)_2_, P(*t*‐Bu)_3_HBF_4_, *t*‐BuONa, reflux, 8 hours, yield: 57%; d) Lawesson's reagent, dry TOL, 110 °C, N_2_, reflux, 18 hours, yield: 37% (**DS‐NI‐DPA**) and 31% (**S‐NI‐DPA**), respectively.

Herein, we used another strategy to tune the photophysical property of the dyads, that is, thionation of the carbonyl groups in the NI chromophore. Replacing the oxygen atom in a carbonyl group of a chromophore with sulfur atom impose a substantial effect on the photophysical property of the chromophore, such as the S_1_ and T_1_ state energy levels of the NI moiety, while keeping a minimal alternation of the structure of the chromophore (one‐atom substitution).,^[^ ^70^, ^71^, ^72^, ^73^, ^74^, ^75^, ^76^ ^]^ First, the ISC ability of the thionated chromophore will be enhanced, which is beneficial to produce ^3^LE state as the precursor of ^3^CS state. Second, thionation of chromophores decreases the LUMO energy, that is, makes the thionated chromophore a stronger electron acceptor compared to the oxo analogue.^[^ ^36^, ^77^, ^78^, ^79^ ^]^ This stronger electron accepting ability will make the CS state energy lower, then it is more likely to reside below the ^3^LE state, which is mandatory for observation of a ^3^CS state. However, thionation of a chromophore may also reduce the ^3^LE state energy level of a chromophore.^[^ ^73^, ^77^, ^78^ ^]^ Therefore, the photophysics of such thionated dyads need to be studied in detail. Herein, we prepared a series of thionated dyads (Scheme 1); the photophysics were studied by using various steady‐state and time‐resolved transient optical spectroscopic methods, and TREPR spectroscopy.

## 2 Results and Discussion

### 2.1 Molecular Structure Design

NI is used as electron acceptor in electron donor–acceptor dyad, which has a high triplet energy level (*E*_T_ = 2.29 eV)^[^ ^80^ ^]^ and the reduction potential is *E*_RED_ = −1.72 V (vs. Fc/Fc^+^).^[^ ^41^ ^]^ PXZ is selected as electron donor, which has oxidation potential (*E*_OX_ = +0.22 V vs. Fc/Fc^+^).^[^ ^81^ ^]^ Previously, we observed TADF property for the **NI‐PXZ** orthogonal dyad. We also observed a ^3^CS state for **NI‐PXZ**, based on the lifetime of the transient species.^[^ ^41^ ^]^ TREPR spectra have indicated the electron spin multiplicity of the ^3^CS and ^3^LE state of **NI‐PXZ** and the SOCT‐ISC mechanism that contribute to the formation of the triplet states of **NI‐PXZ**.^[^ ^38^, ^41^ ^]^

Thionation of the carbonyl group can enhance the electron‐accepting capability of electron acceptor,^[^ ^77^ ^]^ and facilitate the ISC process, while reducing the energy level of the T_1_ state, and the triplet state lifetime of the chromophores. Therefore, to study the effect of thionation on the photophysical properties of TADF molecules, we carried out thionation of the NI chromophore in **NI‐PXZ**. The precursor dyads were prepared using the Buchwald–Hartwig coupling reaction.^[^ ^82^, ^83^ ^]^ The thionated target compounds (**S‐NI‐PXZ**, **DS‐NI‐PXZ**, **S‐NI‐DPA** and **DS‐NI‐DPA**) were prepared by treatment of the **NI‐PXZ** and **NI‐DPA** with Lawesson's reagent (Scheme 1).^[^ ^70^, ^84^ ^]^ The compounds **DS‐NI** and **S‐NI** were used in this study as reference compounds.

To confirm the molecular structure of the **S‐NI‐PXZ**, we investigated the 1D ^13^C NMR, 2D ^1^H − ^1^H Correlation Spectroscopy (COSY) NMR and performed the 2D ^13^C─^1^H Heteronuclear Multiple Bond Correlation (HMBC) NMR spectra (Figures S8 and S9).^[^ ^85^ ^]^ The ^1^H─^1^H COSY NMR spectrum of **S‐NI‐PXZ** shows two characteristic doublet peaks of protons of the thiocarbonylation NI (S‐NI) unit (H_2_ and H_3_ in Figure S8), and the interaction between adjacent characteristic peaks of H_5_, H_6,_ and H_7_, which confirms that PXZ unit is substituted at 4‐position but not at 5‐position. Through long‐range correlation signals for ^13^C─^1^H spin pairs recorded in the HMBC spectra, a chemical shift of 193.17 ppm is observed for the carbon atom at the 1‐position, coupling with H_2_, and 161.04 ppm for the carbon atom at the 8‐position, interacting with H_7_. Due to the larger chemical shift of C_1_ compared to C_8_, the sulfur atom is connected to C_1_. (Figure S9)^[^ ^76^, ^86^ ^]^

By thionation of the NI moiety, the n‐π* transition may facilitate rapid ISC. Moreover, thionation of the carbonyl group of the NI electron acceptor in the compact **NI‐PXZ** dyads enhances the electron‐accepting ability, thus decreases the CS state energy.^[^ ^38^, ^77^ ^]^ The photophysical property of the dyads were studied with steady‐state and time‐resolved optical and electron paramagnetic resonance spectroscopic methods.

### 2.2 UV − Vis Absorption and Fluorescence Spectra

**Figure 1:**
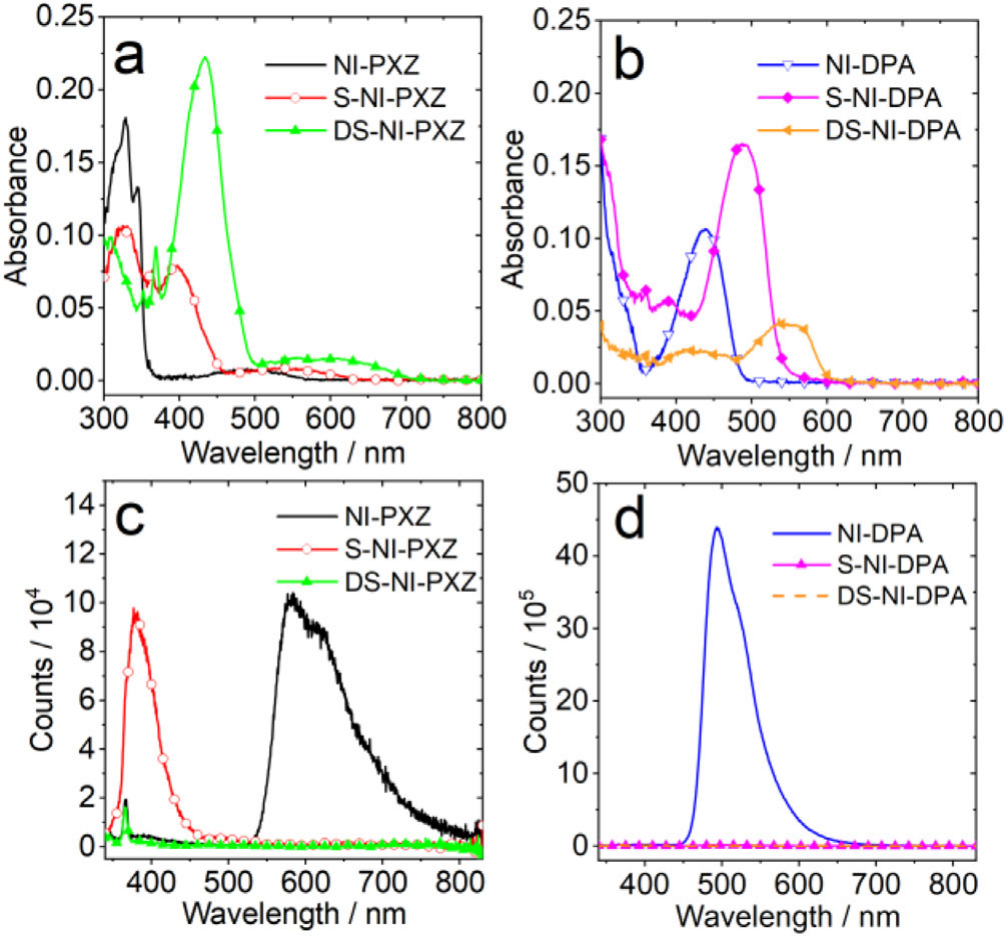
UV − vis absorption spectra of a) **NI‐PXZ, S‐NI‐PXZ** and **DS‐NI‐PXZ,** b) **NI‐DPA, S‐NI‐DPA** and **DS‐NI‐DPA** in *n*‐HEX. *c* = 1.0 × 10^−5^ M. Comparison of the fluorescence spectra of c) **NI‐PXZ, S‐NI‐PXZ,** and **DS‐NI‐PXZ,** d) **NI‐DPA, S‐NI‐DPA,** and **DS‐NI‐DPA** in *n*‐HEX. Optically matched solutions were used (*A *= 0.100 at *λ*_ex_ = 330 nm), 25 °C.

To study the effect of thionation on the photophysical properties of the electron donor–acceptor (D–A) dyads, the UV − vis absorption spectra of the compounds were studied. For **NI‐PXZ** (Figure 1a), the characteristic absorption peaks are located in the range 300–350 nm. This is the typical structured absorption band of NI chromophore in the range of 300–350 nm.^[^ ^87^ ^]^ The weak, broad, structureless absorption band centered at 490 nm is the CT absorption band (S_0_→^1^CT transition).^[^ ^38^, ^88^ ^]^ The absorption bands in the range of 300–440 nm for **S‐NI‐PXZ** are the characteristic absorption of **S‐NI** moiety, based on the absorption spectrum of **S‐NI** (Figure S19a) in *n*‐hexane (*n*‐HEX). Interestingly, we found the CT absorption band of **S‐NI‐PXZ** is centered at 550 nm, which is red‐shifted by 60 nm compared to the CT absorption band of **NI‐PXZ** (490 nm). This effect is attributed to the stronger electron withdrawing ability of the thionated NI unit in **S‐NI‐PXZ**. As the number of sulfur atoms substituting the carbonyl oxygen increases, the CT absorption band is red‐shift to 600 nm. The absorption band of **DS‐NI‐PXZ** shows a more significant red‐shift, with the maximum absorption peak located at 434 nm, which is approximately 100 nm shifted compared to the maximum absorption band of **NI‐PXZ**. Both **DS‐NI‐PXZ** and **S‐NI‐PXZ** possess strong visible light absorption, which is beneficial for application in photodynamic therapy (PDT).^[^ ^89^ ^]^

The UV − vis absorption spectra of the **NI‐DPA**, **S‐NI‐DPA,** and **DS‐NI‐DPA** are presented in Figure 1b. **NI‐DPA** shows a strong CT absorption band centered at 437 nm, which is different from **NI‐PXZ**. The broader absorption bands appeared in the range of 400–600 nm in the thionate derivatives of **NI‐DPA**. Similar red‐shifting of the absorption bands upon thionation was observed for **NI‐DPA**, **S‐NI‐DPA** (centered at 490 nm), and **DS‐NI‐DPA** (centered at 550 nm, Figure 1b). This band is attributed to the CT absorption. The three compounds represent a completely coupled system, for which a *π*‐conjugated framework has been established in the amine N atom and the NI moiety,^[^ ^38^ ^]^ which are different from the weakly coupled systems of the orthogonal configuration molecule dyads discussed above containing PXZ moiety (Figure 1a).

The fluorescence spectra of the compounds are presented in Figure 1c,d. The fluorescence emission band of **NI‐PXZ** is centered as 580 nm in *n*‐HEX, which are broad and structureless, indicating the CT character of the emissive state. We found the fluorescence of **NI‐PXZ** is completely quenched upon thionation (Figure 1c). According to the previous studies on the photoluminescence of the thionated compounds,^[^ ^72^, ^73^ ^]^ we propose that the quenching of the fluorescence is due to the enhanced nonradiative decay channel, such as the ISC process.^[^ ^90^ ^]^

Interestingly, we noticed that **S‐NI‐PXZ** has a fluorescence band centered at 380 nm in *n*‐HEX. The same emission band of **S‐NI‐PXZ** was observed in TOL and dichloromethane (DCM). However, **NI‐PXZ** shows a broad, weaker CT emission band centered at 700 nm in TOL (Figure S20a). Therefore, we attribute the emission band of **S‐NI‐PXZ** centered at 380 nm to the fluorescence of the ^1^LE state. ISC enhanced by the thionation of the carbonyl groups in NI chromophore was also observed for **S‐NI‐DPA** and **DS‐NI‐DPA** (Figure 1d). This result is rationalized by the increased singlet oxygen quantum yield of the compound upon thionation of the carbonyl groups (Table 1).

**Table 1:** Photophysical Data of the Compounds.^[a]^

Compounds	*λ*_abs_[nm]^[b]^	*ε* ^[c]^	*λ*_em_[nm]^[d]^	*Φ*_Δ_[%]^[e]^	τ_F_[ns]^[h]^	τ_T_[µs]^[j]^
**DS‐NI**	432	2.34	‒^[^ ^g^ ^]^	‒^[^ ^g^ ^]^; 46^[^ ^f^ ^]^	‒^[^ ^g^ ^]^	0.2
**DS‐NI‐PXZ**	434	2.21	‒^[^ ^g^ ^]^	21; 24^[^ ^f^ ^]^	‒^[^ ^g^ ^]^	0.25
**S‐NI‐PXZ**	330; 398	1.05; 0.79	380	10; ‒^[^ ^g^ ^]^	2.2	0.66
**DS‐NI‐DPA**	540	0.4	‒^[^ ^g^ ^]^	50; 43^[^ ^f^ ^]^	‒^[^ ^g^ ^]^	2.6
**S‐NI‐DPA**	487	1.64	547	80; 87^[^ ^f^ ^]^	−^[^ ^g^ ^]^	3.0
**NI‐PXZ**	330	1.78	584	7	17.1/12 000^[k]^	11.6
**NI‐DPA**	440	1.06	493	‒^[^ ^g^ ^]^; 45^[^ ^f^ ^]^	10.2^[^ ^i^ ^]^	101.9
**S‐NI**	392	1.46	366	‒^[^ ^g^ ^]^	4.8	0.68

^[a]^ *in n*‐HEX.

^[b]^ Selected relevant UV − vis absorption wavelength, *c* = 1.0 × 10^−5^ M, 20 °C.

^[c]^ Molar absorption coefficient, *ε*: 10^4^ M^−1^ cm^−1^

^[d]^ Maximal fluorescence emission wavelength in nm.

^[e]^ Quantum yield of singlet oxygen, Ru(bpy)_3_[PF_6_]_2_ was used as standard compound (*Φ*_Δ_ = 57% in DCM).

^[f]^ in TOL.

^[g]^ Not observed.

^[h]^ Fluorescence lifetime in *n*‐HEX under air atmosphere at 20 °C.

^[i]^ Literature data.

^[j]^ Triplet lifetime in deaerated solution.

^[k]^ Thermally activated delayed fluorescence lifetime under N_2_ atmosphere at 20 °C.

The photophysical properties of these compounds are summarized in Table 1. As an approximation of ISC efficiency, we measured the quantum yield of singlet oxygen (*Φ*_Δ_) for the compounds (Table 1). For **DS‐NI**, the *Φ*_Δ_ value in TOL is 46%, which is close to the reported *Φ*_Δ_ value of **DS‐NI** in TOL (46%).^[^ ^77^ ^]^ For **S‐NI‐PXZ**, with *Φ*_Δ_ = 10% in *n*‐HEX, no singlet oxygen photosensitizing was observed in TOL. In the case of **DS‐NI‐PXZ**, the *Φ*_Δ_ in *n*‐HEX and TOL are 21% and 24%, respectively. A similar trend was observed for the thionated **NI‐DPA** derivatives. **S‐NI‐DPA** has a high *Φ*_Δ_ (80% *n*‐HEX, 87% in TOL), with a related triplet lifetime *τ*_T_ = 3.0 µs in *n*‐HEX and *τ*_T_ = 7.0 µs in TOL, as measured by ns‐TA spectroscopy. The singlet oxygen quantum yield of **DS‐NI‐DPA** (*Φ*_Δ_ = 50% *n*‐HEX) is lower than that of **S‐NI‐DPA** (*Φ*_Δ_ = 80% *n*‐HEX), and the triplet lifetime of **DS‐NI‐DPA** (*τ*_T_ = 2.6 µs in *n*‐HEX) is also shorter than that of **S‐NI‐DPA** (*τ*_T_ = 3.0 µs in *n*‐HEX). Similar phenomena were also observed in TOL. The singlet oxygen quantum yields of **DS‐NI‐DPA** and **S‐NI‐DPA** were 43% and 87%, respectively. The corresponding triplet state lifetimes were determined to be 3.6 µs and 7.0 µs, respectively. Based on these results, the singlet oxygen quantum yield of the compounds might be related to the triplet state lifetime.^[^ ^74^, ^77^, ^78^ ^]^

**Figure 2:**
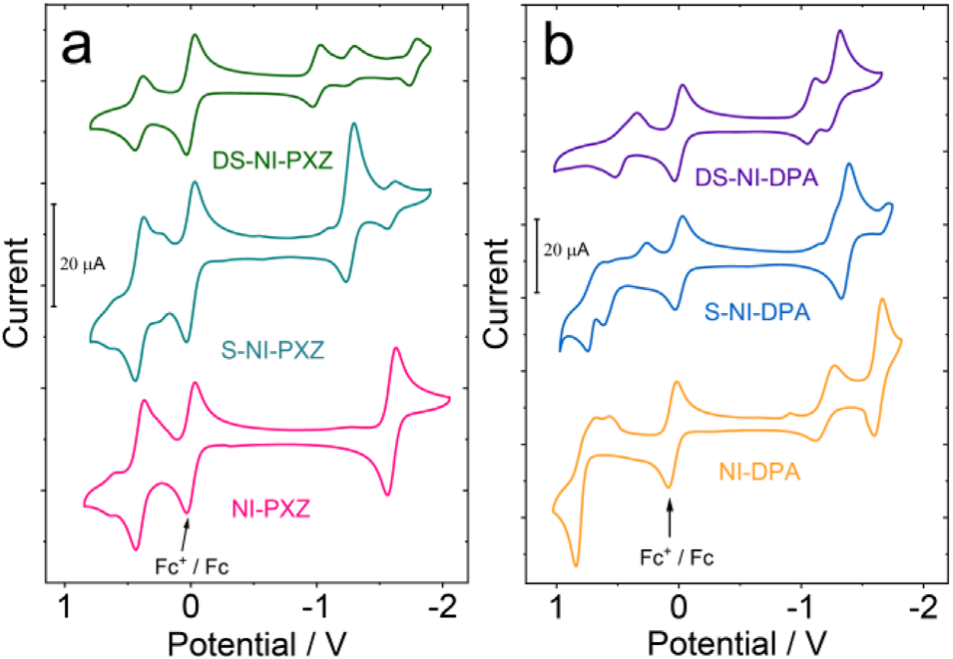
Cyclic voltammograms of the compounds a) **NI‐PXZ**, **S‐NI‐PXZ** and **DS‐NI‐PXZ**, b) **NI‐DPA**, **S‐NI‐DPA,** and **DS‐NI‐DPA** measured in deaerated ACN containing 0.10 M tetrabutylammonium hexafluorophosphate (Bu_4_N[PF_6_]) as the supporting electrolyte. Scan rate: 50 mV/s. Ferrocenium/ferrocene (Fc^+^/Fc) redox couple was used as the internal reference, 25 °C.

The possible reason for the shortening of triplet state lifetime of the thionated compounds is that thionation of the carbonyl groups modifies the frontier molecular orbitals of the T_1_ states, the SOC is enhanced which makes the T_1_ → S_0_ ISC faster. For instance, make the π−π∗ character of the T_1_ → S_0_ transition to n−π∗, which facilitates the ISC, according to the EI Sayed's rule, Figure 2 ^[^ ^91^ ^]^

### 2.3 Electrochemical and Spectroelectrochemical Properties: Cyclic Voltammogram of the Compounds

To calculate the Gibbs free energy changes ($\Delta G_{\mathrm{cs}}^{0}$) associated with the photoinduced charge separation (CS) process and to quantify the energy of the CS states, the redox properties of the compounds were investigated using cyclovoltammetry (Figure 2). The first reversible oxidation wave of **S‐NI‐PXZ** and **DS‐NI‐PXZ** were observed at + 0.41 V (Figure 2a), which is comparable to that of the **NI‐PXZ** (+0.41 V). Thus, the oxidation wave is attributed to the PXZ moiety. Upon thionation of the NI units, reversible reduction waves were observed at −1.25 V and −1.00 V (vs. Fc/Fc^+^) for **S‐NI‐PXZ** and **DS‐NI‐PXZ**, respectively. This reduction wave is attributed to the reduction of the thionated NI moiety.^[^ ^77^ ^]^ Note the reduction wave of the NI moiety is at −1.60 V (vs., Fc/Fc^+^). Thus, the reduction waves of **S‐NI‐PXZ** and **DS‐NI‐PXZ** are anodically shifted by 0.35 V and 0.60 V, respectively, as compared to that of **NI‐PXZ** (Figure 2a). This result indicates the thionated NI is a stronger electron acceptor than the NI moiety. A similar phenomenon was observed for **DS‐NI‐DPA** and **S‐NI‐DPA** dyads (Figure 2b). The reduction potentials of **DS‐NI‐DPA** and **S‐NI‐DPA** were observed at −1.09 V and −1.36 V, respectively, and the potential anodically shifted by 0.59 V and 0.32 V compared with that of **NI‐DPA** (1.68 V, vs. Fc/Fc^+^). This result indicates that thionated NI moiety becomes a stronger electron acceptor than the bare NI. After thionation, the reduction potential of the electron acceptor becomes more positive. These observations are supported by theoretical calculation, which shows that the LUMO energy level of the **DS‐NI‐PXZ** (−3.33 eV) becomes lower than **NI‐PXZ** (−2.64 eV), thus the thionated NI is a stronger electron acceptor. For **S‐NI‐DPA**, the first irreversible oxidation wave is at + 0.27 V (Figure 2b), and **DS‐NI‐DPA** observed a reversible oxidation wave (+0.43 V).

**Figure 3:**
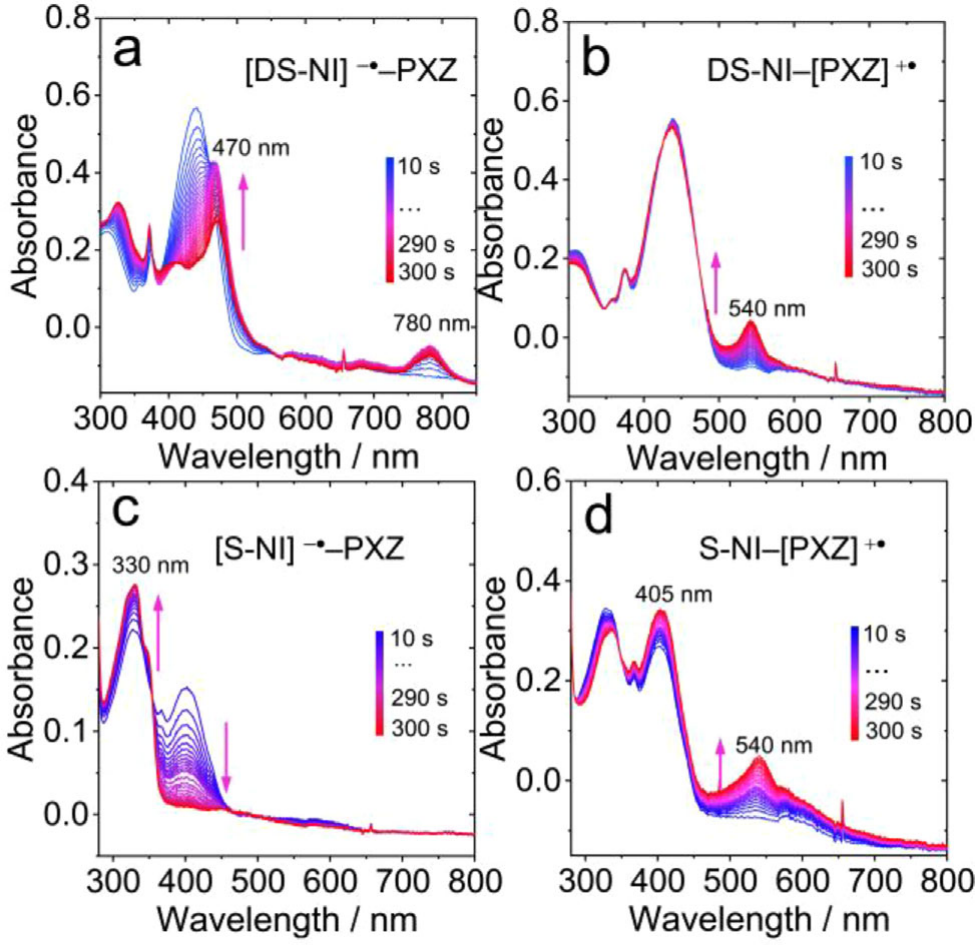
Spectroelectrochemical study of the compounds of a) **DS‐NI‐PXZ** with an applied potential of −1.06 V and b) its oxidation with an applied potential of +0.48 V on the working electrode. c) **S‐NI‐PXZ** with an applied potential of −1.33 V and d) its oxidation with an applied potential of +0.48 V on the working electrode. Ag/AgNO_3_ was used as reference electrode. *c*[**DS‐NI‐PXZ**] = 4.0 × 10^‒5^ M and *c*[**S‐NI‐PXZ**] = 6.0 × 10^‒5^ M in deaerated ACN. 25 °C.

The Rehm–Weller equation (Equations (2)–(5)) was used to determine the thermodynamics for photo‐induced CS and CR processes (Table 2).^[^ ^20^, ^92^, ^93^, ^94^ ^]^ The equation clearly shows that several parameters affect the Gibbs free energy change of electron transfer, such as the redox potentials of the electron donor and acceptor, the excited‐state energy level *E*_00_, and the Coulomb energy (Δ*G*S) which is strongly depends on the polarity of the solvent (dielectric constant and the distance between the electron donor and acceptor). Moreover, the Rehm–Weller equation also provides methods for regulating the energy levels of the CS state, such as by changing the redox potential of the electron D‐A dyads and the center‐to‐center separation distance between electron donors and acceptors.^[^ ^40^, ^42^ ^]^ Energies of the CS states (*E*_CS_) can be calculated with the (Equation 1). The data are collected in Table 2 (Equations 2 − 5. See Supporting Information for the details of the equations).

**Table 2:** Electrochemical Redox Potentials, Gibbs Free Energy of the Charge Separation (Δ*G*_CS_) and Charge Separation Energy Levels (*E*_CS_) of the Compounds in Different Solvents.

			$\Delta G_{\mathrm{cs}}^{0}$ [eV]/*E*_CS_ [eV]			
	*E*_OX_ (V)^[a]^	*E*_RED_ (V)^[a]^	*n*‐HEX	TOL	DCM	ACN
**DS‐NI‐PXZ** ^[b]^	+0.41	−1.00/−1.26	−0.59/2.11	−0.67/1.93	−1.21/1.40	−1.34/1.29
**S‐NI‐PXZ** ^[c]^	+0.41	−1.25	−0.49/2.37	−0.68/2.18	−1.18/1.65	−1.34/1.54
**DS‐NI‐DPA** ^[d]^	+0.43	−1.09/−1.26	−0.83/1.96	−0.95/1.83	−1.33/1.46	−1.41/1.38
**S‐NI‐DPA** ^[e]^	+0.27	−1.36	−0.73/2.11	−0.73/1.97	−1.17/1.58	−0.98/1.49

^[a]^ Redox potentials (vs. Fc^+^/Fc) of the compounds in N_2_‐saturated ACN.

^[b]^ *E*_00_ = 2.70 eV.

^[c]^ *E*_00_ = 2.86 eV.

^[d]^ *E*_00_ = 2.79 eV.

^[e]^ *E*_00_ = 2.84 eV. The energy levels of *E*_00_ are approximated with an average value of the maximal UV − vis absorption and fluorescence emission wavelengths of the thionated‐NI in ACN solution.

For thionated compounds, the $\Delta G_{\mathrm{cs}}^{0}$ is negative in different solvents, hence the ^1^CS process in the singlet state is thermodynamically allowed. Moreover, the relative energy of the ^3^LE state and ^1^CS state was studied. In nonpolar solvents such as *n*‐HEX (Table 2), the ^1^CS state has higher energy than the ^3^LE state (1.71 eV). In polar solvents acetonitrile (ACN), the ^1^CS state energy is lower than that of the ^3^LE state of the DS‐NI moiety (Figure S27). The presence of low‐lying ^3^CS state in polar solvent is confirmed by the ns‐TA spectral studies and the theoretical computation results (see later section). The low‐lying ^1^CS state may be responsible for shortening triplet lifetime observed for **DS‐NI‐PXZ** in polar solvents. However, observing the ^1^CS state is still challenging. The energy of the ^3^LE state of the thionated chromophore decreases, causing the lowest excited state of the molecule to exhibit strong local state properties For example, the unsubstituted **DS‐NI** has a ^3^LE state energy of ca. 1.71 eV (determined with phosphorescence in Figure S27, approximated with the 0,0 transition), which is decreased compared to the nonthionated NI chromophore (2.29 eV).^[^ ^41^, ^87^ ^]^

(2) $$E_{CS}=e\left[E_{OX}-E_{RED}\right]+\Delta G_{S}$$

(3) $$\Delta G_{cs}^{0}=e\left[E_{OX}-E_{RED}\right]-E_{00}+\Delta G_{S}$$

(4) $$\Delta G_{S}=-\frac{e^{2}}{4\pi \varepsilon_{S}\varepsilon_{0}R_{CC}}-\frac{e^{2}}{8\pi \varepsilon_{0}}\left(\frac{1}{R_{D}}+\frac{1}{R_{A}}\right)\left(\frac{1}{\varepsilon_{REF}}-\frac{1}{\varepsilon_{S}}\right)$$

(5) $$\Delta G_{CR}^{0}=-\left(\Delta G_{cs}^{0}-E_{00}\right)$$

Therefore, experimental detection of the CS state is only possible when the lowest energy state is the ^3^CS state rather than the ^3^LE state.^[^ ^77^ ^]^

In order to help the assignment of the CS states in the transient absorption spectra (see later section), we conducted spectroelectrochemical investigation to analyze the absorption features of the radical anion and cation of **S‐NI‐PXZ** and **DS‐NI‐PXZ** (Figure 3).

With a negative potential applied, the absorption band of **DS‐NI‐PXZ** centered at 440 nm gradually decreased, new absorption bands centered at 470, and 780 nm appeared (Figure 3a), which are attributed to the electrochemically generated radical anion ([DS‐NI]^−•^). Similar results were observed for the absorption of [DS‐NI]^−•^ by using a chemical reduction method (Figure S31d). When a positive potential was applied, a new absorption band centered at 544 nm was observed (Figure 3b), which is assigned to the absorption of the radical cation ([PXZ]^+•^).^[^ ^95^, ^96^ ^]^ For **S‐NI‐PXZ**, with a negative potential applied (−1.33 V, vs. Ag/AgNO_3_), the absorption band centered at 400 nm gradually decreased, new absorption peaks at 330 nm appeared (Figure 3c), which are attributed to the electrochemically generated radical anion ([S‐NI]^−•^). When positive potential was applied, new dominant absorption band centered at 405 nm and 540 nm developed, which are attributed to the absorption band of the radical cation ([PXZ]^+•^) (Figure 3d). Similar results were also observed for **S‐NI‐PXZ**, upon reduction with tetrabutylammonium fluoride (TBAF), a decrease in the absorption band at 400 nm and S‐NI^−•^ exhibited absorption bands at 320 nm and 460 nm (Figure S31b). We also calculated the UV − vis spectra of radical anions and cations of **S‐NI‐PXZ** and **DS‐NI‐PXZ**. (Figure S54) The main absorption bands obtained by TDDFT calculations are in good agreement with the experimental results. It should be noted that the UV − vis absorption spectra of the radical anions and cations generated in spectroelectrochemical study may differ from the photoexcited CS state, because in the spectroelectrochemical study, the anions and cations are not present simultaneously.

### 2.4 Femtosecond Transient Absorption Spectra

**Figure 4:**
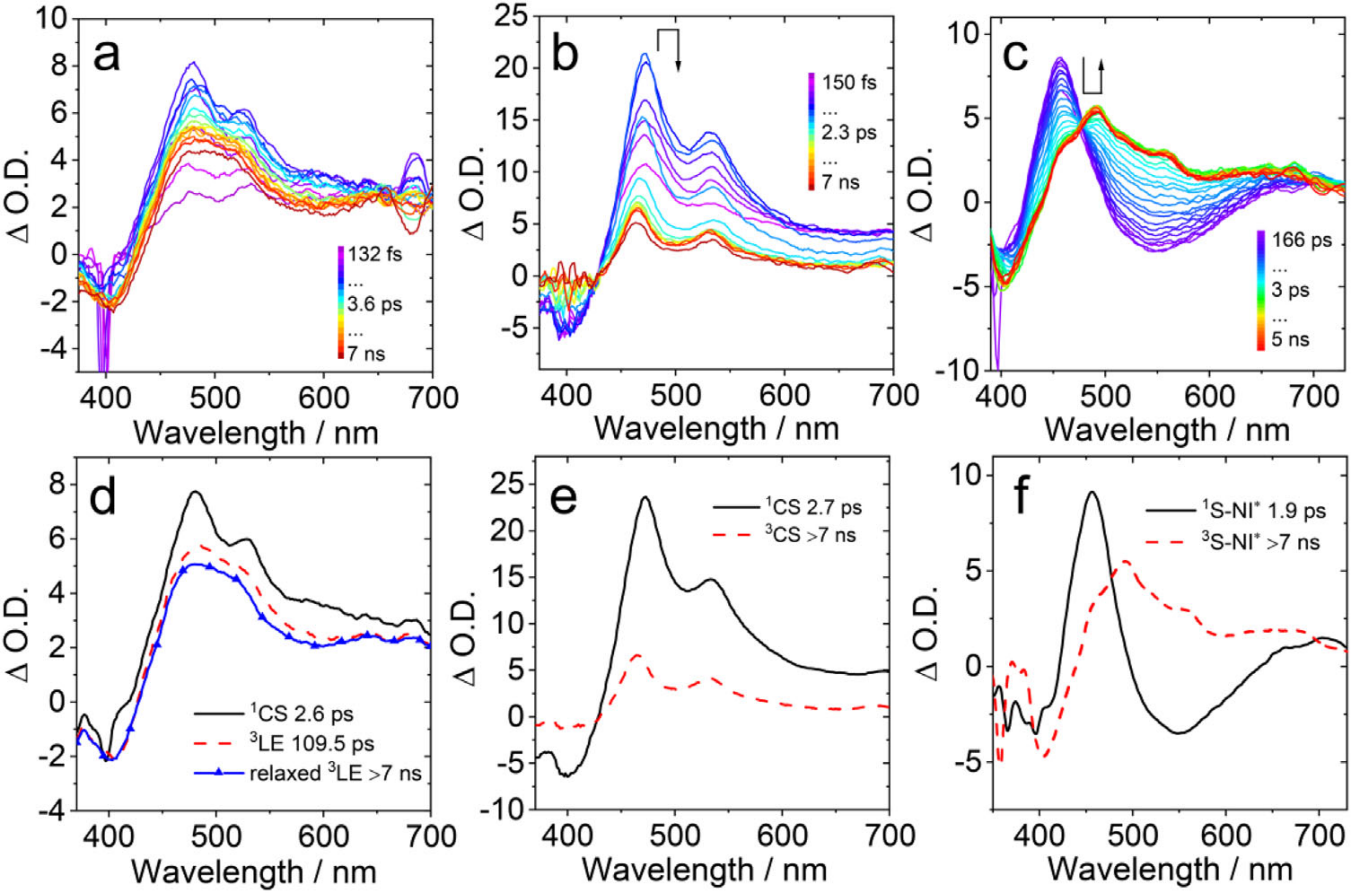
Femtosecond transient absorption spectra of **S‐NI‐PXZ** in a) *n*‐HEX, b) ACN. The related evolution is associated with difference spectra (EADS) in d) *n*‐HEX, e) ACN for **S‐NI‐PXZ**. c) The fs‐TA spectra and f) the related EADS of **S‐NI** in *n*‐HEX. EADS were obtained from target analysis with the sequential model. *λ*_ex_ = 355 nm. *c* = 3.0 × 10^−5^ M, 25 °C.

The photophysical properties of thionated compounds, in particular the ISC and the CS process, were investigated with femtosecond transient absorption (fs‐TA) spectroscopy (Figure 4).^[^ ^97^ ^]^ The data (Figure 4a–c) were analyzed with singular value decomposition (SVD), global fitting and target analysis using a linear unidirectional decay model. The evolution‐associated difference spectra (EADS) were obtained (Figure 4d–f). Both apolar and polar solvents were considered for the monosubstituted **S‐NI** and **S‐NI‐PXZ** and the disubstituted **DS‐NI** and **DS‐NI‐PXZ**.

In the case of **S‐NI**, the fs‐TA spectra in *n*‐HEX are shown in Figure 4c. The result of a target analysis of the fs‐TA data with a sequential model was obtained (Figure 4f). The S_1_ state of **S‐NI** with an ESA band was centered at 455 nm and 710 nm. The ground‐state bleaching (GSB) band was observed in the range of 350–420 nm (Figure 4f), which agrees with the steady‐state absorption spectrum (Figure S19a). Then, the ^3^LE state with a broad ESA band in the range of 470−560 nm was generated by the fast ISC process within 1.9 ps from the ^1^LE state of **S‐NI**, which was attributed to the spin orbit coupling ISC (SOC‐ISC) mechanism, enhanced by the thionation.^[^ ^98^ ^]^ Assuming that the decay of ^1^LE state of **S‐NI** was mainly attributed to the ISC, the *k*_ISC_ value was measured to be 5.26 × 10^11^ s^−1^.^[^ ^99^ ^]^ The ISC process for **S‐NI** is faster than for the unsubstituted NI, with ISC time constants of 10–20 ps.^[^ ^100^ ^]^ Similar results were observed for **S‐NI** in ACN with the ISC process of 1.8 ps (Figure S33). The ^3^LE state did not decay completely within the available time window of the fs‐TA spectrometer, which is supported by the ns‐TA spectra (Figure S47e).

The fs‐TA spectra of **S‐NI‐PXZ** in different solvents are shown in Figure 4a,b, together with the EADS obtained from global analysis. Upon pulsed laser excitation at 355 nm in *n*‐HEX, we observed two ESA peaks at 480 nm and 530 nm together with a broad absorption band in the range of 600–700 nm.

Based on spectroelectrochemical spectra (Figure 3c,d), the absorption band centered at 480 nm is assigned to radical anion [S‐NI]^−•^ and the absorption band centered at 530 nm is attributed to the PXZ radical cation ([PXZ]^+•^). By comparison with the spectra of **S‐NI** presented in Figure 4f, the first species of **S‐NI‐PXZ** cannot be assigned as the ^1^LE state of S‐NI moiety, because the sharp absorption peak at 530 nm is different from the spectrum of **S‐NI** in *n*‐HEX. We also used a fs‐TA spectrometer with a shorter time window (1 ps and 1 ns) to further analyze the time evolution of **S‐NI‐PXZ** (Figures S35a and S35b). Absorption peaks at 480 nm and 530 nm were observed within a short time window of 1 ps, which are the same as the spectra of long‐time windows of 7 ns. Therefore, the first species of **S‐NI‐PXZ** in *n*‐HEX is classified as ^1^CS state. The transition from ^1^LE to ^1^CS state is shorter than the instrument response function (IRF) of the fs‐TA spectrometer (ca. 130 fs). After 2.6 ps, the second species was formed with broad absorption bands in the range of 470–570 nm, which is attributed to the ^3^[S‐NI]* state produced by the CR of **S‐NI‐PXZ**. The ISC rate of **S‐NI‐PXZ** was calculated as *k*_ISC_ = 3.8 × 10^11^ s^−1^. Within 109.5 ps the intensity of the ESA band decreased slightly and blue shifts by about 5 nm, indicating a vibrational relaxation of the ^3^[S‐NI]* state. Thus, the process of **S‐NI‐PXZ** upon photo‐excitation in *n*‐HEX can be summarized as S_1_ → ^1^CS → ^3^S‐NI*. In ACN, initial EADS showed positive absorption bands centered at 472 nm and 530 nm (Figure 4e). This species is attributed to the ^1^CS state of **S‐NI‐PXZ**, which is similar to the ^1^CS signal of **S‐NI‐PXZ** observed in *n*‐HEX (Figure 4d). After 2.7 ps, the transient spectrum evolved significantly, with the appearance of two positive bands peaking at 464 nm and 533 nm respectively. By comparing the absorption spectra of S‐NI^−•^ and PXZ^+•^, generated through spectroelectrochemical and chemical reduction methods (Figure S31b), we assign these species attributed to the ^3^CS state. We propose a short‐living intermediate ^3^LE state may be involved, with the ^3^LE → ^3^CS faster than the ^1^CS → ^3^LE process. Thus, the photophysical process for **S‐NI‐PXZ** in ACN is S_1_ → ^1^CS → ^3^CS state. The final spectral signal lived beyond the time range explored with the fs‐TA setup (7 ns), which is consistent with the results of the subsequent ns‐TA spectroscopy measurement.

The fs‐TA spectra of **DS‐NI** and **DS‐NI‐PXZ** were also studied. For **DS‐NI** at early delay times after pulsed laser excitation, a broader positive band centered at 517 nm was observed. This band is assigned to the ESA of the S_1_ state of **DS‐NI** in *n*‐HEX (Figure S32b). The ^3^LE state with the absorption peaks at 503 nm and 730 nm was produced by the ISC process within 6.4 ps. This is in line with the previous research results. The ^1^LE state of **DS‐NI** generates the ^3^LE state through rapid ISC (10.9 ps) in TOL.^[^ ^77^ ^]^ For **DS‐NI‐PXZ**, based on the EADS obtained from global analysis, the assignment of transient species requires careful consideration. In *n*‐HEX, the first species of **DS‐NI‐PXZ** showed a broad positive band centered at 517 nm, and the GSB band peaked at 440 nm (Figure S36d), which is similar to the ^1^LE state spectrum of **DS‐NI**. The first species of **DS‐NI‐PXZ** is attributed to ^1^DS‐NI*. The subsequent vibrational relaxation of the ^1^LE state takes 0.6 ps, and the ESA peak becomes weaker, and it was blue‐shifted to 510 nm. After 4.1 ps, ESA bands centered at 495 nm and 537 nm were observed. It should be noted that the positive band at 537 nm is the absorption of the radical cation [PXZ]^+•^, which is confirmed by spectral‐electrochemistry (Figure 3b). However, we consider that the positive band at 495 nm may be distorted by the GSB band and should not be simply assigned to the radical anion [DS‐NI]^−•^. Therefore, the CS process in **DS‐NI‐PXZ** is faster than the ISC process that generates the ^3^DS‐NI* state through the SOCT‐ISC mechanism These species did not decay to the baseline in the detection time window of fs‐TA spectroscopy, which is the same as that observed in the ns‐TA spectra initially. By comparing with the ns‐TA spectra, we assigned the last contributions to a mixed state of ^3^CS and ^3^LE for **DS‐NI‐PXZ** in *n*‐HEX (see later section).

In previous fs‐TA studies on the TADF molecule NI‐PXZ, a similar ^3^CT/^3^LE hybrid state was also observed.^[^ ^41^ ^]^ In ACN, initial EADS of **DS‐NI‐PXZ** showed the appearance of ESA peaked at 517 nm (Figure S36f), which can be assigned to the ^1^DS‐NI* as previously discussed. Then, the ISC process occurs within 1 ps to produce the triplet state and the ESA band at 501 nm appeared. After 7.6 ps, the transient spectrum evolved, with the appearance of two positive bands peaking at 490 nm and 537 nm, respectively. We assign these bands to the [DS‐NI]^−•^ radical anion and [PXZ]^+•^ radical cation. Consequently, this species is attributed to the ^3^CS state and in accordance with the ns‐TA spectra result. The final spectral component lived beyond the time explored with the fs‐TA measurement (7 ns). Thus, the photophysical process for **DS‐NI‐PXZ** in ACN is ^1^LE → ^3^LE → ^3^CS state.

In comparison the **NI** shows ISC of *k*_ISC_ = 8.69 × 10^10^ s^−1^.^[^ ^41^ ^]^ Interestingly, the ISC kinetic of **DS‐NI‐PXZ** (*k*_ISC_ = 2.4 × 10^11^ s^−1^) is similar to that of the **DS‐NI** (*k*_ISC_ = 1.56 × 10^11^ s^−1^), which is slightly slower than the ISC kinetics of **S‐NI‐PXZ** (*k*_ISC_ = 3.8 × 10^11^ s^−1^) and **S‐NI** (*k*_ISC_ = 5.26 × 10^11^ s^−1^) in *n*‐HEX. Therefore, we found that increasing the number of S atom substitutions cannot continuously enhance the ISC ability of such compact D‐A dyads photosensitizers.

### 2.5 Nanosecond Transient Absorption Spectra

**Figure 5:**
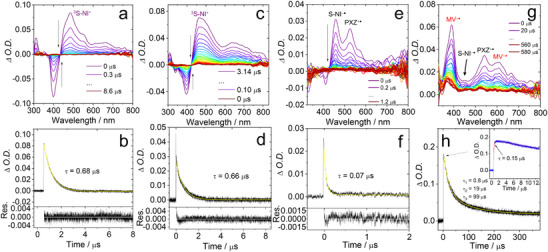
Nanosecond transient absorption spectra of a) **S‐NI**, and c) **S‐NI‐PXZ** in *n*‐HEX, e) **S‐NI‐PXZ** alone and g) **S‐NI‐PXZ** in the presence of MV^2+^ (*c* **=** 3.0 × 10^−5^ M) in deaerated ACN. Decay traces of b) **S‐NI** at 500 nm, and d) **S‐NI‐PXZ** at 460 nm in deaerated *n*‐HEX, f) **S‐NI‐PXZ** alone at 460 nm and h) **S‐NI‐PXZ** in the presence MV^+•^ at 390 nm in deaerated ACN. Excited with nanosecond pulsed laser. *λ*_ex_ = 355 nm. *c*[**S‐NI**] = 2.0 × 10^−5^ M. *c*[**S‐NI‐PXZ**] = 3.0 × 10^−5^ M. 25 °C.

To verify the transient species formed with **NI‐PXZ, S‐NI‐PXZ, DS‐NI‐PXZ,** and reference compounds upon photoexcitation, the ns‐TA spectra in *n*‐HEX were studied (Figure 5). The ns‐TA spectra recorded in solvents of different polarities are shown in the Supporting Information. The ns‐TA spectra of compounds upon pulsed laser excitation at 355 nm were recorded. For **NI‐PXZ**, a positive absorption band at 410 nm was observed, which is assigned to the NI^−•^ absorption (Figure S42a). The shoulder band at 460 nm is attributed to the ESA band of the ^3^NI* state, which is the same as that of the reference compound, that is, the unsubstituted NI and the 4‐bromoNI.^[^ ^101^ ^]^ This is in line with our previous studies, in which the triplet state signal of **NI‐PXZ** was observed as an admixture of the ^3^LE and ^3^CS states in n‐HEX.^[^ ^41^, ^68^ ^]^

For the reference compound **S‐NI**, the broad ESA bands centered at 484 nm were observed in *n*‐HEX (Figure 5a). The ^3^LE state lifetime of **S‐NI** is 0.68 µs (Figure 5b). For the dithionated compound, spectra of **DS‐NI** are presented in Figure S41. In *n*‐HEX, the broad absorption band centered at 500 nm and 720 nm is attributed to the absorption of ^3^LE state. The triplet state lifetime of the dithionated compound is further shortened: by monitoring the decay trace at 500 nm it was determined as 0.20 µs, and it is independent of the solvents. Note that this lifetime is much shorter than the triplet excited state of pristine NI (*τ*_T_ = 58.9 µs).^[^ ^41^ ^]^ We attribute this effect to the strong SOC effect imposed by the thiocarbonyl group, strong SOC improves the S_1_ → T_1_ transition (in nπ*→ππ* transition, see later section), but also the relaxation of T_1_ → S_0_.^[^ ^78^, ^79^, ^102^, ^103^ ^]^

**S‐NI‐PXZ** and **S‐NI** exhibit the same characteristic ESA bands in the range of 480–750 nm. The ns‐TA spectrum of **S‐NI‐PXZ** could not show the ^1^CS intermediate state observed by fs‐TA, but just the derived ^3^S‐NI* state with an ESA band at 470 nm (Figure 5c). The decay of ^3^S‐NI* state of **S‐NI‐PXZ** is 0.66 µs (Figure 5d) which is similar to the triplet state lifetime of **S‐NI** (0.68 µs). As the solvent polarity increases, variation of the ns‐TA spectra of **S‐NI‐PXZ** was observed. In ACN, two absorption bands centered at 460 nm and 530 nm were observed (Figure 5e), similar to the signals of the [S‐NI]^−•^ and [PXZ]^+•^ generated by spectroelectrochemical (Figure 3c, d). Therefore, the lowest triplet state of **S‐NI‐PXZ** in ACN should be the ^3^CS state, because the femtosecond transient absorption spectroscopy shows that the ^1^CS state rapidly converts to the ^3^CS state via ISC process within 2.7 ps, and it does not fully decay within the 7 ns.^[^ ^38^, ^41^, ^43^ ^]^ Therefore, the lifetime of the ^1^CS state may be too short to be detected by the ns‐TA spectrometer (IRF >10 ns). The ^3^CS state of **S‐NI‐PXZ** was also observed in tetrahydrofuran (THF) and DCM, no absorption band for the ^3^LE state was observed (Figure S49). As the solvent polarity increased, the triplet state lifetime gradually shortened (0.12 µs in THF and 0.10 µs in DCM). This may be attributed to the decreased CS state energy in polar solvent. The ^3^CS state lifetime of **S‐NI‐PXZ** in ACN was shortened to 0.07 µs (Figure 5f), compared with that of unthionated **NI‐PXZ** (0.13 µs in ACN, Figure S42f). This result indicates that the thionation of the NI moiety leads to a significant shortening of the T_1_ state lifetime.^[^ ^10^ ^]^ On the other hand, the triplet state lifetime of **S‐NI** was less dependent on the solvent polarity (Figure S47).

To further corroborate the formation of the CS state for **S‐NI‐PXZ**, quenching experiment of the radical anion was conducted. This study involved the addition of 1,1′‐dimethyl‐4,4′‐bipyridinium (MV^2+^) to the solution, then the absorption of the radical anion was monitored by the ns‐TA spectral changes.^[^ ^104^, ^105^ ^]^ When MV^2+^ was added to the solution of **S‐NI‐PXZ** in ACN (Figure 5g), electron transfer took place from [S‐NI]^−•^ to MV^2+^, and MV^+^ **^.^** radical cation should be produced. A possible rationale for this process involves the reduction potential of MV^2+^ (*E*_red_ = −0.82 V, Figure S29) compared to the oxidation potential of the anion of **S‐NI‐PXZ** (*E*_ox_ = −1.25 V). Therefore, the MV^2+^ has a stronger electron‐accepting ability. The ns‐TA spectra show that for the mixture of **S‐NI‐** **PXZ** and MV^2+^, the absorption band of the radical cation [PXZ]^+•^ of **S‐NI‐PXZ** at 540 nm still exists in the presence of MV^2+^. However, the absorption band centered at 460 nm of [S‐NI]^−•^ disappeared and the new positive absorption bands centered at 390 nm and 620 nm (assigned to MV^+•^) developed.^[^ ^104^ ^]^ Moreover, in the presence of MV^2+^, the decay of the [PXZ]^+•^ absorption band, centered at 540 nm, become much slower (*τ*_T_ = ca. 92 µs). Because with quenching of the radical anions in [S‐NI]^−•^‐[PXZ]^+•^, the [PXZ]^+•^ can only decay via intermolecular CR with MV^+•^. This is supported by the lifetime of MV^+•^ (*τ* = ca. 100 µs).

In the case of **NI‐PXZ**, under the presence of the MV^2+^ in ACN, the absorption peak of the radical anion NI^−•^ at 430 nm disappeared, while the absorption bands of the MV^+•^ at 390 nm and 620 nm appeared (Figure S44). Similar results were also observed for **DS‐NI‐PXZ** in the presence of MV^2+^ in ACN (Figure S45).

Following pulsed laser excitation, a broad positive absorption band of **DS‐NI‐PXZ** spanning from 300 to 600 nm was observed (Figure S50), overlapping with the GSB band centered at 430 nm. The GSB absorption band agrees with the UV **− **vis absorption spectrum (Figure 1a), and compared to **NI‐PXZ**, the GSB absorption band of the thionated **NI‐PXZ** exhibits a redshift of 100 nm. **DS‐NI‐PXZ** exhibited the ESA band centered at 500 nm and 700 nm in *n*‐HEX, which is the characteristic ESA band of the ^3^DS‐NI* state.^[^ ^77^ ^]^ But we noticed that the absorption band located at 763 nm does not belong to ^3^DS‐NI* but it is a characteristic absorption band of the [DS‐NI]^−•^ of **DS‐NI‐PXZ** (refer to the spectroelectrochemical and chemical reduction study, Figure 3). We also observed an absorption band of the radical cation [PXZ]^+•^ at 535 nm, however, the characteristic peak of [DS‐NI]^−•^ at 475 nm was not observed, possibly due to overlap with the GSB band. Thus, for **DS‐NI‐PXZ**, we observed the CS state and the ^3^DS‐NI* state simultaneously, which is similar to the previous observation for **NI‐PXZ** All the transient bands decay with the same kinetics, the lifetime was determined as 0.25 µs. Similar ns‐TA spectra of **DS‐NI‐PXZ** were observed in cyclohexane (CHX) (Figure S51a). In ACN, the positive absorption band (located at 500 nm) of **DS‐NI‐PXZ** was blue‐shifted to 480 nm (Figure S51e). And the ESA band of ^3^[DS‐NI]* centered at 700 nm for **DS‐NI‐PXZ** disappears. The positive absorption band at 780 nm is attributed to DS‐NI^−•^, which is similar to the absorption spectra of DS‐NI^−•^ generated by chemical reduction (Figure S31d), demonstrating the formation of a ^3^CS state. The CS state we observed in the ns‐TA spectra is most likely a ^3^CS state, because the fluorescence of **DS‐NI‐PXZ** was quenched and no fluorescence emission from the ^1^CS state was observed (Figure 1d). The lifetime of the ^3^CS state is much shorter (0.03 µs. Figure S50f) compared to that in *n*‐HEX (τ_T_ = 0.25 µs). The possible reason is that the energy level of CS state is highly dependent on the solvent polarity, whereas the ^3^LE stale energy levels are almost independent of the solvent polarity. The CS state energy becomes lower in polar solvents, making the ^3^CS state lifetime shorter.^[^ ^67^, ^90^ ^]^

**Figure 6:**
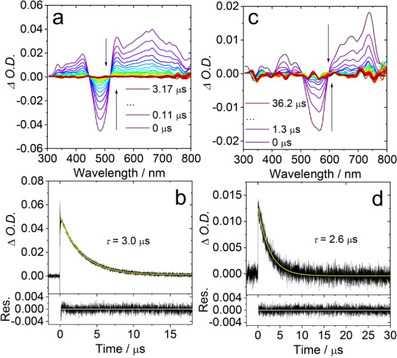
Nanosecond transient absorption spectra of a) **S‐NI‐DPA** (*λ*_ex_ = 500 nm), and c) **DS‐NI‐DPA** (*λ*_ex_ = 550 nm). Decay traces of b) **S‐NI‐DPA** at 535 nm and d) **DS‐NI‐DPA** at 685 nm. *c*[**S‐NI‐DPA**, **DS‐NI‐DPA**] = 3.0 × 10^−5^ M in deaerated *n*‐HEX, 25 °C.

For **S‐NI‐DPA** (Figure 6a), the ESA bands of triplet states in the range of 310–440 nm and 520–800 nm were observed. By combining the results of triplet state spin density and molecular frontier orbital distribution from DFT calculations, we assign the observed transient species to the intramolecular charge transfer (ICT) state. The triplet state lifetime of **S‐NI‐DPA** was determined as 3.0 µs (Figure 6b). In TOL and ACN, we observed similar broad ESA bands, and the GSB bands  in the range of 450−540 nm overlapped with the ESA peaks. The π electrons of thionated **NI‐DPA** derivatives are delocalized throughout the molecule (Figure 9c,d), leading to stronger electronic coupling between DS‐NI and DPA moieties.^[^ ^106^ ^]^ Transient spectra of **DS‐NI‐DPA** show a GSB band at around 510 − 600 nm, which is red‐shifted by 60 nm compared to the GSB peak of **S‐NI‐DPA** in *n*‐HEX. (Figure 6c) Two broad ESA bands in 350−500 nm and 600−800 nm were observed, which are attributed to the ICT state absorption, corresponding to DFT computation. The triplet state lifetime of **DS‐NI‐DPA** was determined as 2.6 µs. Similar results were found in other solvents (TOL and ACN) (Figure S53). For **NI‐DPA**, triplet state signals are observed in low‐polarity solvents (Figure S46). We observed a GSB band centered at 450 nm, an ESA band centered at 370 nm and a second positive band above 470 nm. But in high‐polarity solvents, no ns‐TA signal is observed for **NI‐DPA**, indicating no significant production of long‐lived triplet state for this compound. Notably, the triplet state lifetime of **NI‐DPA** (101.9 µs in *n*‐HEX, Figure S46b) is much longer than the triplet state lifetimes of **S‐NI‐DPA** (3.0 µs) and **DS‐NI‐DPA** (2.6 µs). Therefore, we conclude that while sulfur atoms promote ISC to generate triplet states, the presence of sulfur atoms reduces the lifetime of triplet states.^[^ ^78^, ^79^ ^]^

### 2.6 Time‐Resolved Electron Paramagnetic Resonance (TREPR) Spectroscopy

**Figure 7:**
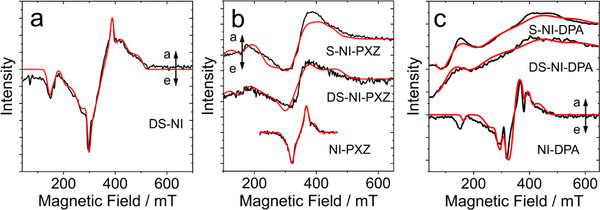
TREPR spectra of a) **DS‐NI**, b) **S‐NI‐PXZ**, **DS‐NI‐PXZ** and **NI‐PXZ** and c) **S‐NI‐DPA**, **DS‐NI‐DPA** and **NI‐DPA** recorded at 80 K (black traces) at the maximum of the transient intensity (ca 1 µs after the laser flash). The solvent was TOL/2‐MeTHF (3:1, *v/v*), with concentration of the compounds at 1.0 × 10^−4^ M. The red lines are simulations with the parameters reported in Table3.

The transient paramagnetic species of the compounds, for instance the localized triplet excited state (^3^LE) or the triplet CS state (^3^CS), or the free radical pairs, formed upon photoexcitation can be selectively detected by TREPR spectroscopy.^[^ ^107^, ^108^, ^109^, ^110^, ^111^, ^112^ ^]^ The TREPR spectra of the compounds were obtained by excitation of the samples with pulsed laser at 355 nm in a glassy low‐polarity matrix (TOL/2‐MeTHF 3:1) at 80 K. Irradiation in the visible range (excitation in the CT band) did not provide signal, likely because of the low molar absorption coefficients of the compounds. The TREPR spectra are displayed in Figure 7.

For **DS‐NI**, a broad spectrum was observed. With electron spin polarization (ESP) pattern of the triplet state spectrum is (*e*, *e*, *e*, *a*, *a*, *a*), which is common in triplet states of organic chromophores populated via the SOC‐ISC mechanism (Figure 7a). The same ESP pattern was observed for native NI (*e*, *e*, *e*, *a*, *a*, *a*),^[^ ^98^ ^]^ although the transitions at some canonical orientations are less pronounced. Interestingly, the **DS‐NI** triplet state shows a much larger ZFS *D* parameter of 4200 MHz than the triplet excited state of the native NI compound (ZFS *D* is ca. 2472 MHz).^[^ ^38^, ^41^, ^113^, ^114^ ^]^ Recently with thionated NI, coumarin and perylenebisimide chromophores, we observed the same trend.^[^ ^77^, ^78^, ^79^ ^]^ We attributed the large ZFS *D* parameters to the large SOC effect.

First, we recorded the TREPR spectrum of **NI‐PXZ** (Figure 7b); we observed a spectrum with ESP of *e*, *e*, *e*, *a*, *a*, *a* (*e*: emission; *a*: enhanced absorption), and the ZFS *D* parameter of 1544 MHz, which is close to the previous study of the dyad.^[^ ^41^ ^]^ This ZFS *D* parameter is much smaller than the triplet state of the unsubstituted NI chromophore (ZFS *D* is ca. 2500 MHz).^[^ ^41^ ^]^ We attribute the decreased ZFS *D* parameter either to the fast equilibrium of the ^3^CS and the ^3^LE state,^[^ ^41^ ^]^ or to a broader extension of the wavefunction on the larger molecule. For **NI‐DPA**, a triplet state spectrum with ESP pattern of *e*, *e*, *e*, *a*, *a*, *a* was observed (Figure 7c), simulation of the spectrum gives ZFS *D* of 2940 MHz, which is slightly larger than the ZFS *D* parameter of the triplet state of unsubstituted NI.^[^ ^38^, ^114^ ^]^ The half‐field transition suggests the presence of another triplet with large ZFS but it was not possible to characterize it.

For **S‐NI‐PXZ**, the electron ESP pattern of e,a,e,a,e,a was observed, which is common in triplet states of organic chromophores formed by the SOCT‐ISC effect. The population rates of the three sublevels of the T_1_ states are *Px: Py: Pz* = 1.0: 0.6: 0.0. The TREPR spectra of **DS‐NI‐PXZ** were studied, and a spectral shape similar to that of **S‐NI‐PXZ** was observed (Figure 7b). Interestingly, TREPR spectral study shows that the *D*‐parameter is particularly large for **S‐NI‐PXZ**, **DS‐NI‐DPA** and **S‐NI‐DPA**. The ZFS of these compounds vary by an order of magnitude in a range between |*D*| = 882 MHz and |*D*| = 9800 MHz. To analyze the TREPR spectral data, we have initially calculated the dipolar contribution to the ZFS for the triplet state in the point dipole approximation for the rigid NI and dithio naphthalimide (**DS‐NI**) systems, and we found a value of *D* = +4280 and +3140 MHz, respectively. The sign is positive because the electronic distribution has an oblate (disk‐like) prevalence.^[^ ^110^ ^]^ The value is a reference for **DS‐NI** and for the other systems, where the triplet excited state is localized in the NI and DS‐NI moieties. Indeed, for **DS‐NI**, **S‐NI‐PXZ**, **DS‐NI‐PXZ,** and **NI‐DPA** we find triplets with *D*‐values between 2940 and 4200 MHz, which are then attributed to triplets with excitation localized on the NI, S‐NI or DS‐NI moiety (^3^LE), respectively. For **NI‐DPA** the smallest *D*‐value of the series is observed, with an analogous behavior as for the excited singlet, where the excitation is extended not just to the NI moiety (see the red‐shift in the UV‐vis absorption spectra). This system also shows a second triplet state with a relatively small |*D*| value of 882 MHz, that we attribute to a ^3^CS state. This corresponds to a distance between the charges of ca. 4.4 Å, which is consistent with an unpaired electron on the NI moiety and another unpaired electron on the amino nitrogen atom. We observed another trend that for the thionated NI chromophore, the 4‐amino substituent will greatly increase the ZFS *D* parameter. For example, both **S‐NI‐DPA** and **DS‐NI‐DPA** show much larger ZFS |*D*| parameters than that of **DS‐NI**. The ZFS |*D*| parameters of **DS‐NI‐PXZ** and **S‐NI‐PXZ** (9800 MHz) are much larger than that of **DS‐NI** [Table chem70388-tbl-0003].

**Table 3 chem70388-tbl-0003:** ZFS fitting parameters *D* and *E* of the TREPR spectra whose simulations are reported in Figure 7. The uncertainty is estimated to be around 1%. *D *> 0 and *E *> 0 values were assumed for the population attribution.

	|*D*|[MHz]	|E|[MHz]	*P*x:*P*y:*P*z	Δ*P* ^[a]^
**DS‐NI**	4200	1260	0.72:1.00:0.00	0.28
	2604	28	0.60:1.00:0.00	0.40
**NI‐PXZ**	1544	30	0.56:1.00:0.00	0.56
**DS‐NI‐PXZ**	4050	750	1.00:0.63:0.00	0.59
	9800	196	1.00:1.00:0.00	0.00
**S‐NI‐PXZ**	3450	750	1.00:0.6:0.00	0.66
	9800	196	1.00:1.00:0.00	0.00
**NI‐DPA**	882	126	0.72:1.00:0.00	0.28
	2940	140	0.60:1.00:0.00	0.40
**DS‐NI‐DPA**	7965	675	1.00:0.85:0.00	0.18
**S‐NI‐DPA**	7484	800	1.00:0.91:0.00	0.10

^[a]^ Δ*P* = |*Px*−*Py*| / |*Py*−*Pz*|.

Note that for the thionated dyads, no ^3^CS state was observed under the experimental condition.^[^ ^28^, ^38^ ^]^ The discrepancy with fs‐TA and ns‐TA spectral result (in which CS state was observed) is probably due to the decreased polarity of the frozen solution as compared to the fluid solution at room temperature, and the elevated CS state energy, as a result, the ^3^LE state is the state with the lowest energy.

For the **DS‐NI** system we obtained also another triplet species with smaller ZFS values. We attribute this minor contribution to the formation of molecular clusters, in fact, the short triplet lifetime measured at RT (see Table 1) forced us to work with relatively high concentration in order to have a good S/N ratio. We noticed that most of the studied systems exhibit the presence of a second species, with *D*‐values much larger than the calculated dipolar contribution, that cannot attributed to clustering of the molecules. In this case, the hypothesis is that in these systems the observed second triplet state is characterized by a large spin‐orbit contribution because of the presence of sulfur.

According to the previous report, the ZFS parameter *D* value of thionated coumarins (8600 MHz) is larger than that of non‐thionated coumarin (|*D*| = 3000 MHz).^[79]^ A similar phenomenon has also been observed in thionated PBI derivatives, the ZFS parameter *D* values (1625−1992 MHz) of thionated PBIs are larger than those of the pristine PBI chromophore (*D* = 1166 MHz).^[78]^ The ZFS parameter *D* is the sum of the dipole‐dipole interaction (${\hat{D}}_{\mathrm{dip}}$) and the spin‐orbit interactions (${\hat{D}}_{so}$) terms. ${\hat{D}}_{so}$ is closely associated with the SOC effect in triplet states. ^[78,79]^ This causes large |*D*| values (4200−9800 MHz) of the thionated compounds, whereas the |*D*| value of the nonthionated NI‐PXZ is smaller (1544 MHz). Therefore, the larger *D* values in thionated chromophores are essentially a manifestation of thionation‐induced SOC enhancement.

We performed the calculation of the spin orbit coupling (SOC) matrix elements 〈*T*_m_|*H*_SO_|*S*_n_〉 (m, n = 1, 2, …), which are connected to the ISC population/depopulation rates of the triplet levels. Since it is expected that low‐energy excited singlet states contribute more significantly than the upper singlet excited states to the ISC, we considered mostly transitions from S_1_ and S_2_ levels. Moreover, we selected elements between states with matched energy (normally ± 2000 cm^−1^, compatible with band linewidths). The results are shown in Tables S4 and S5.

In Table S4, the sum of the squared SOC matrix elements (SOC‐MEs) of the ISC (decay to the ground state: T_1_→S_0_) of **DS‐NI‐PXZ** (*k*_tot_ = ca. 18 336 cm^−2^) and **DS‐NI** (*k*_tot_ = ca. 18 580 cm^−2^) are larger than that of **NI** (9.6 cm^−2^).

Table S5 shows that the larger SOC effect between *T*_m_ and *S*_n_ states (population of the triplets) are observed for thionated compounds, which justifies larger ISC rate constants (*k*_ISC_). The *k*_tot_ describes direct SOC (Further details are provided in Section 1.7 in the Supporting Information). For **NI**, **S‐NI**, and **DS‐NI‐PXZ**, the *k*_tot_ values are 229 cm^−2^, 2500 cm^−2^, and 1628 cm^−2^, respectively, which is in line with the *k*_ISC_ of 0.8 × 10^11^ s^−1^, 5.26 × 10^11^ s^−1^, and 2.4 × 10^11^ s^−1^ for **NI**, **S‐NI**, and **DS‐NI‐PXZ**, respectively.

### 2.7 DFT Computations

The ground state (S_0_) geometry of the compound was optimized (Figure S56). The dihedral angles between the thionated NI and the PXZ moieties of **DS‐NI‐PXZ** and **S‐NI‐PXZ** are 84.1° and 84.6°, respectively, which are similar to the dihedral angle of **NI‐PXZ** (84.4°, Figure S55a). For **NI‐DPA, S‐NI‐DPA** and **DS‐NI‐DPA**, the dihedral angles are 58.8°, 56.9°, and 58.8°, respectively, thus, we expect the strong electronic coupling between the two moieties, which is supported by the intense CT absorption band in the UV − vis spectrum (Figure 1b). Conversely, **DS‐NI‐PXZ** and **S‐NI‐PXZ** have an approximately orthogonal dihedral angle, indicating a weak electron coupling between the NI and PXZ moieties. This is supported by the observation of a weak CS absorption band in the UV − vis absorption spectrum (Figure 1a).

**Figure 8:**
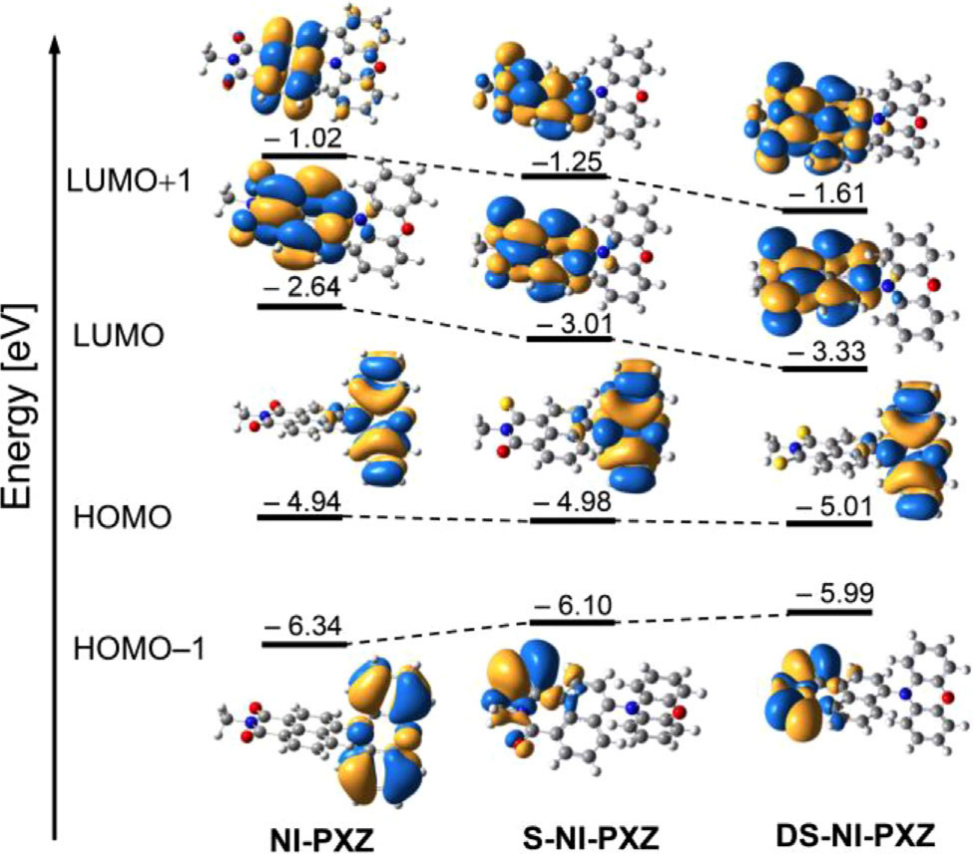
Selected molecular orbitals (isovalues = 0.02) and the corresponding energies (in eV) of **NI‐PXZ**, **S‐NI‐PXZ,** and **DS‐NI‐PXZ**, based on the optimized ground state geometries, respectively. In gas phase, calculated at the CAM‐B3LYP/6–31G(d) level with Gaussian 16.

The molecular orbitals (MOs) of **NI‐PXZ**, **S‐NI‐PXZ** and **DS‐NI‐PXZ** are presented in Figure 8. The HOMO is exclusively localized on the PXZ moiety since the PXZ acts as the donor unit. The LUMO and LUMO + 1 are confined on the electron acceptor moiety (NI, S‐NI and DS‐NI). The occupation of these frontier MOs indicates weak electronic coupling between the electron donor and acceptor. The electron cloud distributions of the HOMO and LUMO show almost no spatial overlap, which facilitates the electron transfer after the compounds are photoexcited. This is also helpful to achieving efficient SOCT‐ISC.^[^ ^115^ ^]^ Interestingly, compared to the dyad containing a native NI moiety of **NI‐PXZ** (*E*_LUMO_ = −2.64 eV), the LUMO energy of **S‐NI‐PXZ** and **DS‐NI‐PXZ** is decreased to −3.01 eV and −3.33 eV, respectively. This can be attributed to the electron‐withdrawing effect of the sulfur atom. The HOMO energy level remains almost unchanged due to the minimal distribution of the HOMO on the NI moiety. The narrowing of the HOMO/LUMO energy gap affect the photophysical properties of **S‐NI‐PXZ** and **DS‐NI‐PXZ**, such as the red shift of the UV − vis absorption bands (Figure 1a). The lower LUMO energy contributes to the anodic shift of reduction potentials in the thionated NI‐PXZ derivatives (refer to Tables 2 and S2).

**Figure 9:**
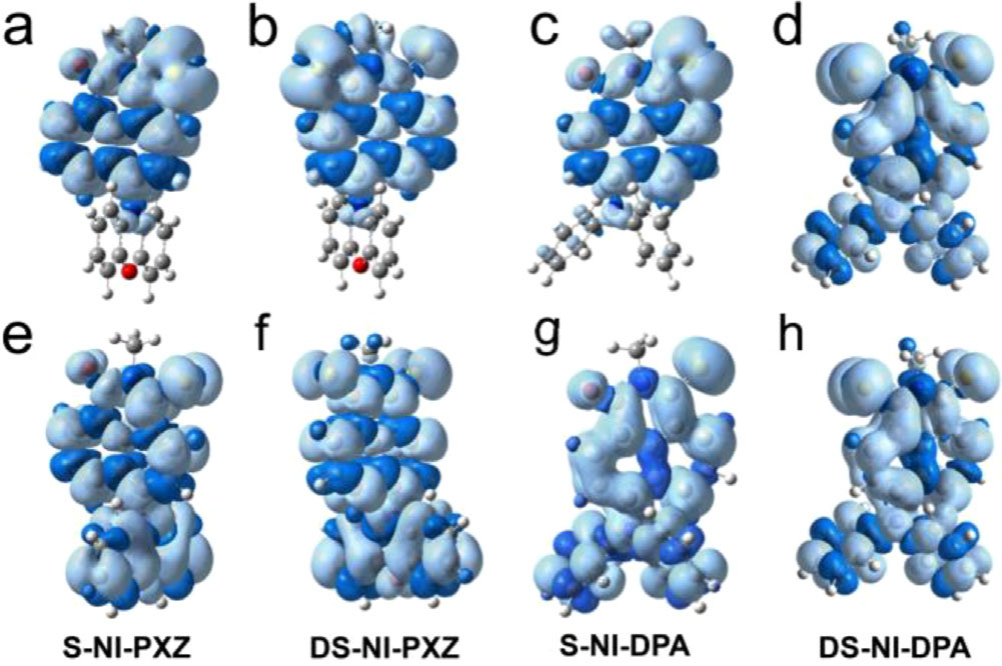
Spin density surfaces of the triplet state of a) **S‐NI‐PXZ**, b) **DS‐NI‐PXZ**, c) **S‐NI‐DPA** and d) **DS‐NI‐DPA** in CHX and e) **S‐NI‐PXZ**, f) **DS‐NI‐PXZ**, g) **S‐NI‐DPA** and h) **DS‐NI‐DPA** in ACN. Calculated at the CAM‐B3LYP/6–31G(d) level with Gaussian 16 (isovalue = 0.0004).

Based on the electrochemical and spectral data, the calculated CS state energy (Table 2), the CS state energy levels of thionated NI‐PXZ are lower than those of unthionated NI‐PXZ in polar solvents. For example, the CS state energy level of **NI‐PXZ** is 1.83 eV in ACN, while those of **S‐NI‐PXZ** and **DS‐NI‐PXZ** are 1.54 eV and 1.35 eV, respectively. The ^3^LE state energy of the native NI chromophore is 2.23 eV.^[^ ^41^, ^87^ ^]^ For **DS‐NI**, this energy decreases to 1.71 eV, which is close to the previously reported phosphorescence energy of the DS‐NI analogue.^[^ ^77^ ^]^ Although the energy level of the ^3^CS state decreases after thionation, the energy level of the ^3^LE state also decreases. This is the reason why the ^3^LE state rather than the CS state was observed in *n*‐HEX via ns‐TA spectra.

The spin density of all four compounds exhibit delocalized T_1_ states (Figure 9). This is in agreement with the ns‐TA spectral results. In polar solvent (ACN), the triplet states of both **S‐NI‐PXZ** and **DS‐NI‐PXZ** are delocalized over the entire molecule, meaning that the lowest‐energy triplet state (T_1_) is the CS state, which is consistent with the ns‐TA spectral results (Figures 6 and S47). For thionated NI‐PXZ derivatives, the contribution of the sulfur atom to the spin density is by an in‐plane p‐orbital, indicating that the n→π* transition is involved in the lowest triplet excited state of thionated chromophores. The sulfur atoms make a significant contribution. This is also consistent with the large |*D*| values of the triplet state of thionated chromophores.

To reveal the effect of thionation of the electron D‐A dyads on their photophysical property, we used steady‐state/transient spectroscopy, TREPR spectroscopy to conduct a detailed investigation into the CS state and ISC process. Electrochemical measurements and DFT calculations were employed to assist in the analysis of spectra results. Through detailed analysis and discussion, it was found that the results from various parts mutually validate each other. This further clarifies the photophysical processes of thionated electron D‐A dyads.

**Scheme 2:**
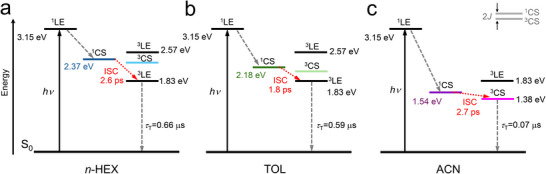
Simplified Jablonski diagram illustrating the photophysical processes involved in **S‐NI‐PXZ** in solvents of (a) n‐HEX, (b) TOL and (c) ACN. The energy of ^1^CS state is approximated from electrochemical data. The energy of the singlet state and the triplet state are from calculation at TDDFT CAM‐B3LYP/6–31G(d) level with Gaussian 16. *J* is the electronic exchange energy, and the energy level difference between ^1^CS and ^3^CS states is 2 *J*. The number of the superscript designates spin multiplicity.

The energy diagrams of **S‐NI‐PXZ** are presented in Scheme 2. Thionation of the carbonyl groups of **S‐NI‐PXZ** enhances the electron‐withdrawing capability of the NI acceptor, thereby increasing the charge separation kinetics. In *n*‐HEX, **S‐NI‐PXZ** demonstrates an ultrafast formation of the ^1^CS state in the fs‐TA spectroscopy (ca. 0.15 ps) compared to **NI‐PXZ** (ca. 0.8 ps).^[^ ^41^ ^]^ Then, the strong SOC of **S‐NI‐PXZ** promotes the ISC process (2.6 ps) for triplet state generation. Therefore, the ^3^LE state was observed via ns‐TA spectroscopy (0.66 µs). Notably, the ^3^LE state energy level (1.83 eV) in **S‐NI‐PXZ** is lower than the native ^3^NI state (2.23 eV) in *n*‐HEX and TOL. In TOL, analogous to the photophysical processes in *n*‐HEX, **S‐NI‐PXZ** generates the ^3^LE state via ISC (1.8 ps). In ACN, enhanced solvent polarity stabilizes the CS state, lowering its energy level to 1.38 eV, which facilitates the generation of ^3^CS via ISC process (2.7 ps).

For **DS‐NI‐PXZ** (Scheme S1), taking the ^1^LE state (2.86 eV) as the precursor, it reaches the T_n_ state through rapid ISC (4.6 ps) in *n*‐HEX. Then, the T_n_ state undergoes internal conversion to the T_1_ state (^3^LE, 1.71 eV). A mixture of the ^3^LE and ^3^CS states is observed in the ns‐TA spectrum, but the TADF of **DS‐NI‐PXZ** is not detected, possibly due to the large Δ*E*_ST_ values (ca. 0.4 eV) between the ^1^CS and ^3^LE states. In ACN, the energy of the CS state is further reduced, leading to the observation of the ISC process from ^1^CS to ^3^CS (1.0 ps). The low T_1_ state energy and the strong spin–orbit coupling interaction may contribute to the shorter triplet state lifetime.

## 3 Conclusion

In summary, the carbonyl group in electron donor–acceptor dyads was thionated, monothionated and dithionated NI‐PXZ and NI‐DPA derivatives were prepared to understand the effect of thionation on the charge separation and the ISC process and the detailed triplet state properties. We found that thionation of the carbonyl groups in **NI‐PXZ** and **NI‐DPA** lead to red‐shift of the UV − vis absorption (ca. 50−100 nm), and strong quenching of the fluorescence as compared to the oxo analogous. Sulfur atoms significantly enhance the spin‐orbit coupling (SOC) of D‐A dyads, leading to the observation of efficient ISC, indicated by the increased singlet oxygen quantum yield (*Φ*_Δ_) from 7% to 24%. The femtosecond transient absorption spectra of **S‐NI‐PXZ** and **DS‐NI‐PXZ** showed fast ISC (*k*_ISC_ = 3.8 × 10^11^ s^−1^ and *k*_ISC_ = 2.4 × 10^11^ s^−1^, respectively) compared with the ISC rate of oxo counterpart **NI‐PXZ** ((*k*_ISC_ = 6.5 × 10^9^ s^−1^). The ISC rate constants of **S‐NI** (*k*_ISC_ = 5.3 × 10^11^ s^−1^) and **DS‐NI** (*k*_ISC_ = 1.6 × 10^11^ s^−1^) were also observed. In comparison the **NI** shows ISC of *k*_ISC_ = 8.69 × 10^10^. Interestingly, the increase in ISC rate is independent of the number of sulfur atoms. Compared with native NI, the energy of the ^3^LE state in thionated NI is reduced by approximately 0.52 eV. In nanosecond transient absorption spectra, the ^3^LE state (*τ*_T_ = 0.66 µs) of **S‐NI‐PXZ** was observed in *n*‐HEX. No TADF was observed in *n*‐HEX, due to the large Δ*E*_ST_ between the ^1^CS and ^3^LE state (ca. 0.4 eV). In ACN, the ^3^CS state of **S‐NI‐PXZ** and **DS‐NI‐PXZ** was observed, which have lifetimes of 0.07 µs and 0.03 µs, respectively. The existence of the CS state was confirmed by photoinduced intermolecular electron transfer experiments performed using electron acceptor methylviologen (MV^2+^) with thionated D‐A dyads, and the radical anion of the CS states of the dyads was selectively quenched, and the electron transfer product MV^+•^ was observed. Pulsed laser excited time‐resolved electron paramagnetic resonance (TREPR) spectra show unusually large zero field splitting (ZFS) *D* parameter for dyads with the thionated NI‐PXZ (|*D*| = 9800 MHz). This is consistent with the calculation results of SOC matrix elements (SOCMEs). The SOCMEs of the T_1_→S_0_ ISC **DS‐NI‐PXZ** (ca. 135 cm^−1^) and **DS‐NI** (ca. 136 cm^−1^) are larger than **NI** (ca. 3.1 cm^−1^), which corresponds to the large |*D*| value (4200−9800 MHz) of the thionated compounds. In comparison the T_1_ state of the NI chromophore gives much smaller ZFS |*D*| parameter (ca. 1544 MHz). Another trend is the introduction of 4‐amino substituent on the thionated NI chromophore will greatly increase the magnitude of the ZFS *D* parameter. No ^3^CS state was observed for the thionated dyads in the TREPR spectral studies. This information is useful for the understanding of photophysics, especially the charge separation, CR and ISC of the compact electron donor–acceptor dyads.

## Supporting Information

General experimental methods, synthesis of compounds, molecular structure characterization, computational details, and additional spectra are provided in the Supporting Information. The additional references have been cited within the Supporting Information.

## Conflicts of Interest

The authors declare no conflict of interest.

## Supporting information



Supporting Information

## Data Availability

The data that support the findings of this study are available in the Supporting Information of this article.
