# Supplementary material for: Effect of Thionation of the Carbonyl Groups in Naphthalimide‐Phenoxazine Electron Donor–Acceptor Dyads on the Excited‐ State Dynamics: Transient Optical and Electron Paramagnetic Resonance Spectral Studies
Source: Chemistry. 2025 Nov 10;31(71):e02885. doi: 10.1002/chem.202502885 (PMC12734687; doi:10.1002/chem.202502885)
Supplement: Supplementary file 1 — Supporting Information [file CHEM-31-e02885-s001.pdf]

Contents

1. General Experimental Information and Synthesis ······ S2
2. NMR and HRMS Spectra of compounds. ······ S6
3. Steady State UV–vis Absorption ······ S16
4. Fluorescence Emission and Fluorescence lifetime Spectra ······ S16
5. Low-temperature Luminescence and Lifetime ······ S20
6. Singlet oxygen quantum (Φ_{Δ}) yields ······ S21
7. Electrochemical study, Spectro-electrochemistry and the Chemical Reduction Study ······ S21
8. Femtosecond Transient Absorption Spectra ······ S24
9. Nanosecond Transient Absorption Spectra ······ S30
10. Theoretical Calculations ······ S40
11. References ······ S47

1. General Experimental Information and Synthesis

1.1. General Methods and Material. All the chemicals used in synthesis are analytically pure and used without purification. The solvents were dried and distilled before synthesis. For measuring samples under deaerated condition, the sample solution was purged with N_2 for 15 minutes prior to measurement. ^{1}H and ^{13}C NMR spectra were recorded on the Bruker Avance spectrometers (400 MHz, 500 MHz, or 600 MHz). High-resolution mass spectrometry (HRMS) was measured with a LTQ Orbitrap XL MS spectrometer or matrix-assisted laser desorption ionization time-of-flight mass spectrometer (MALDI–TOF–MS). UV-2550 spectrophotometer (Shimadzu Ltd., Japan) was used to collect UV–vis absorption spectra. The fluorescence emission spectra of the compounds were recorded on a FS5 spectrofluorometer (Edinburgh Instruments, U.K.). The luminescence lifetimes were measured with an OB920 luminescence lifetime spectrometer (Edinburgh Instruments, U.K.).

1.2. Synthesis of Starting Materials. NI-Br, **DS-NI**, **NI-PXZ**, and **NI-DPA**, were synthesized following the literature methods.

Compound S-NI-PXZ and DS-NI-PXZ. Compounds **NI-PXZ** (263 mg, 0.6 mmol) was dissolved in dry toluene (25 mL), then Lawesson's reagent (889 mg, 2.2 mmol) was added under N_2 atmosphere. The mixture was refluxed at 110 °C for 18 h. After the reaction mixture was cooled to room temperature, the solvent was evaporated under reduced pressure to give a crude mixture of **DS-NI-PXZ** and **S-NI-PXZ**. The crude product was purified by column chromatography (silica gel, DCM/PE = 1:3, v/v). **DS-NI-PXZ** was obtained as a black powder (R_f = 0.7, 67 mg, yield: 24%). M.p.: 186.8–187.7 °C. ^{1}H NMR (400 MHz, $CDCl_3$) δ 9.06 (d, *J* = 8.1 Hz, 1H), 8.94 (d, *J* = 8.6 Hz, 1H), 8.37 (d, *J* = 9.3 Hz, 1H), 7.70 (d, *J* = 8.0 Hz, 1H), 7.61 (t, *J* = 8.0 Hz, 1H), 6.82–6.75 (m, 2H), 6.71–6.67 (m, 2H), 6.54–6.50 (m, 2H), 5.72 (d, *J* = 7.9 Hz, 2H), 5.40–5.32 (m, 2H), 1.93 (t, *J* = 7.9 Hz, 2H), 1.50 (d, *J* = 7.5 Hz, 2H), 1.02 (t, *J* = 7.4 Hz, 3H). ^{13}C NMR (151 MHz, $CDCl_3$) δ = 190.5, 190.0, 143.8, 141.0, 138.9, 138.6, 133.5, 130.5, 130.3, 129.5, 128.8, 128.5, 125.5, 123.5, 122.2, 115.9, 113.4, 77.2, 77.0, 76.8, 54.9, 29.7, 27.4, 20.0, 13.8. MALDI–TOF–HRMS ($C_{28}H_{22}N_2OS_2^-$): calcd *m/z* 466.1180; found *m/z* 466.1169. **S-NI-PXZ** was obtained as a dark brown powder (R_f = 0.4, 55 mg, yield: 20%). M.p.: 172.8–173.4 °C. ^{1}H NMR (400 MHz, $CDCl_3$) δ 9.19 (d, *J* = 8.0 Hz, 1 H), 8.66 (d, *J* = 7.1 Hz, 1H), 8.46–8.38 (m, 1H), 7.83–7.74 (m, 1H), 7.73–7.63 (m, 1H), 6.78 (d, *J* = 7.1 Hz, 2H), 6.72–6.68 (m, 2H), 6.54–6.52 (m, 2H), 5.75–5.65 (m, 2H), 4.82–4.71 (m, 2H), 1.83 (t, *J* = 7.7 Hz, 2H), 1.54–1.48 (m, 2H), 1.02 (t, *J* = 7.4 Hz, 3H). ^{13}C NMR (151 MHz, $CDCl_3$) δ = 193.8, 161.0, 143.8, 141.1,

137.9, 137.5, 133.6, 133.1, 132.8, 130.4, 130.1, 129.7, 129.1, 128.2, 123.5, 122.2, 115.9, 113.4, 77.0, 76.8, 47.3, 31.9, 29.7, 20.4, 13.8. MALDI–TOF–HRMS ($C_{28}H_{22}N_2O_2S^-$): calcd *m/z* 450.1408; found *m/z* 450.1418.

Compound S-NI-DPA and DS-NI-DPA. [1] **NI-DPA** (150 mg, 0.36 mmol) was dissolved in dry toluene (15 mL), then Lawesson's reagent (525 mg, 1.3 mmol) was added under N_2 atmosphere. The reaction mixture was allowed to reflux at 110 °C under N_2 atmosphere for 18 h. The reaction mixture was cooled to room temperature, and the solvent was evaporated under reduced pressure to give a mixture of **DS-NI-DPA** and **S-NI-DPA**. The crude product was purified by column chromatography (silica gel, DCM/PE = 1:2, v/v.). **DS-NI-DPA** was obtained as a purple viscous liquid (R_f = 0.6, 59 mg, yield: 37%). ^{1}H NMR (400 MHz, DMSO–d_6) δ 8.81–8.73 (m, 2H), 8.15 (d, *J* = 9.9 Hz, 1H), 7.57–7.53 (m, *J* = 7.8 Hz, 1H), 7.35 (d. *J* = 8.0 Hz, 1H), 7.34–7.29 (m, 4H), 7.15–7.12 (m, 2H), 7.04 (d, *J* = 7.1 Hz, 4H), 5.31–5.20 (m, 2H), 1.88–1.78 (m, 2H), 1.45–1.37 (m, 2H), 0.96 (t, *J* = 6.6 Hz, 3H). MALDI–TOF–HRMS ($C_{28}H_{24}N_2S_2^+$): calcd *m/z* 452.1375; found *m/z* 452.1374. **S-NI-DPA** obtained a dark red powder (R_f = 0.4, 47 mg, yield: 31%). M.p.: 56.0–56.8 °C. ^{1}H NMR (400 MHz, DMSO–d_6) δ 8.89 (d, 1H), 8.46 (d, 1H), 8.16 (d, 1H), 7.69–7.61 (m, 1H), 7.39 (d, *J* = 4.6 Hz, 1H), 7.37–7.29 (m, 4H), 7.16–7.07 (m, 2H), 7.02 (d, 4H), 4.67–4.61 (m, 2H), 1.75–1.69 (m, 2H), 1.42–1.36 (m, 2H), 0.95 (t, *J* = 7.2 Hz, 3H). MALDI–TOF–HRMS ($C_{28}H_{24}N_2OS^+$): calcd *m/z* 436.1604; found *m/z* 436.1602.

Compound NI-Br. 4-Bromo-1,8-naphthalic anhydride (3.00 g, 10.83 mmol) and n-butylamine (2.50 mL) were mixed in ethanol (90.0 mL). The resulting mixture was stirred for 10 h at 80 °C under N_2 atmosphere. After cooling to room temperature, the solvent was removed under reduced pressure to give a faint yellow solid. The crude product was purified by column chromatography (silica gel, DCM/HEX = 1:1, v/v). Compound **NI-Br** was obtained as a white solid. Yield: 3.2 g (88%). ^{1}H NMR (400MHz , $CDCl_3$) δ 8.66 (d, *J* = 7.3 Hz, 1H), 8.57 (d, *J* = 8.5 Hz, 1H), 8.42 (d, *J* = 7.9 Hz, 1H), 8.04 (d, *J* = 7.9 Hz, 1H), 7.89–7.81 (m, 1H), 4.22–4.13 (m, 2H), 1.78–1.66 (m, 2H), 1.51–1.39 (m, 2H), 0.98 (t, J = 7.3Hz, 3H).

Compound NI-DPA. NI-Br (331 mg, 1 mmol), diphenylamine (204 mg, 1.200 mmol), $Pd(OAc)_2$ (22.4 mg, 0.1mmol) and sodium *tert*-butoxide (386 mg, 4.00 mmol) were mixed in dry toluene (10 mL). Then tri-*tert*-butylphosphine tetrafluoroborate (58.0 mg, 0.200 mmol) was added under N_2 atmosphere. The mixture was stirred for 8 h at 120 °C. After cooling, saturated NaCl solutions was added, and the mixture was extracted three times with dichloromethane (DCM). The organic layers were combined and dried over anhydrous Na_2SO_4. The solvent was evaporated under reduced pressure. The crude product was

purified by column chromatography (silica gel, DCM/PE = 1:1, v/v). Compound **NI-DPA** was obtained as an orange solid. Yield: 243 mg (57 %). ^{1}H NMR (400MHz, DMSO–d_6) δ 8.47 (d, J = 8.0 Hz, 1H), 8.44 (d, J = 7.3 Hz, 1H), 8.16 (d, J = 8.5 Hz, 1 H), 7.69–7.65 (m, 1H), 7.43 (d, J = 8.0 Hz, 1H), 7.33–7.29 (m, 4H), 7.10–7.07 (m, 2H), 6.99 (d, J = 8.0 Hz, 4H), 4.03-4.07 (m, 2H), 1.67–1.59 (m, 2H), 1.40–1.33 (m, 2H), 0.93 (t, J = 7.3 Hz, 3H).

Compound NI-PXZ. NI-Br (794.4 mg, 2.4 mmol), phenoxazine (528.0 mg, 2.89 mmol), $Pd(OAc)_2$ (54.0 mg, 0.24mmol) and sodium tert-butoxide (632.2 mg, 4.40 mmol) were mixed in dry toluene (60 mL). In the nitrogen atmosphere, tri-*tert*-butylphosphine tetrafluoroborate (139.2 mg，0.29 mmol) was added. The mixture was stirred for 10 h at 120 °C. After cooling, saturated NaCl solutions was added, and the mixture was extracted three times with dichloromethane (DCM). The organic layer was dried over anhydrous Na_2SO_4, and the solvent was evaporated under reduced pressure. The crude product was purified by column chromatography (silica gel, DCM/PE = 1:1, v/v). Compound **NI-PXZ** was obtained as a dark red solid. Yield: 447.1 mg (48%). ^{1}H NMR (400 MHz , CDCl3) δ 8.79 (d, J = 7.6 Hz, 1H), 8.66 (d, J = 7.3 Hz, 1H), 8.43 (d, J = 9.3 Hz, 1H), 7.84 (d, J = 7.6 Hz, 1H), 7.78–7.70 (m, 1H), 6.79 (d, J = 7.9 Hz, 2H), 6.68–6.71 (m, 2H), 6.54–6.50 (m, 2H), 5.69 (d, J = 7.9 Hz, 2H), 4.26–4.20 (m, 2H), 1.76 (t, J = 7.6 Hz, 2H), 1.51–1.45 (m, 2H), 1.00 (t, J = 7.4 Hz, 3H).

1.3. Electrochemical Studies. Record the cyclic voltammetry curve using a CHI610D Electrochemical Workstation by Shanghai CHI Instruments Co., Ltd., China. A platinum electrode was used as the counter electrode and a glassy carbon electrode as the working electrode. Ferrocene (Fc) was used as an internal reference (set as 0 V in the cyclic voltammograms). Spectroelectrochemical measurements were conducted using a quartz electrochemical cell with a 0.1 cm optical path length. Use a platinum mesh as the working electrode and a platinum wire as the counter electrode. CHI610D electrochemical workstation was used to regulate the potential and the spectra was recorded by an Agilent 8453 UV-vis spectroscopy system (Agilent Technologies Inc., USA). The spectra were recorded in situ with a spectroelectrochemical cuvette (1 mm optical path). Conduct electrochemical measurements with $Bu_4N[PF_6]$ as the supporting electrolyte. $Ag/AgNO_3$ (0.1 M in acetonitrile, ACN) couple as the reference electrode. ACN was used as the solvent and the solution was purged with N_2 for 15 min before measurement. During the measurement process, N_2 atmosphere was maintained.

1.4. Nanosecond Transient Absorption Spectroscopy. The nanosecond time-resolved transient absorption spectroscopy (ns TA) of the compounds were studied by LP980 laser flash photolysis

spectrometer (Edinburg Instruments, UK) equipped with Tektronix TDS 3012B oscilloscope. Solutions of the compounds were excited with a nanosecond pulsed laser (Surelite I-10, USA; the wavelength being tunable in the range of 210 – 2400 nm), typical laser power is ca. 10 mJ per pulse. The data (kinetic decay traces and spectra) were analyzed with the L900 software. To improve the signal-to-noise ratio, a collinear configuration of the pump and probe beams was adopted for the measurements. All samples in flash photolysis experiments were used with N_2 for 15 min before measurement and the cuvettes were sealed during the measurement.

1.5. Femtosecond Transient Absorption Spectroscopy. Femtosecond transient absorption spectra (fs-TA) data were obtained on an ultrafast transient absorption (TA) spectrometer (Harpia-TA, Light Conversion). The output of an amplified Ti: sapphire laser (800 nm, 40 fs, 1 kHz, Coherent Astrella) was split into two beams. The temporal resolution of the instrument is 80 fs. Excitation pulses at 340 nm were produced by a commercial parametric amplifier (TOPAS, Light Conversion) using two BBO crystals. The probe pulses were obtained by focusing the remaining part of the laser output on a CaF_2 (2 mm) plate. The polarization angle between the pump beam and the probe beam has been set to the magic angle. The sample is placed in a 1 mm quartz cuvette, and the sample is mounted on a movable platform to avoid photo-decomposition. All data were corrected for chirping before the global fitting. The data were fitted with the Glotaran-Application 1.5.1 and applied to a linear sequential model.[2]

1.6. Time-Resolved Electron Paramagnetic Resonance (TREPR) Spectra. The TREPR spectra were recorded using quartz tubes (3 mm i.d.) containing solutions in toluene/2-methylTHF (3:1, v/v) with the concentrations that ensured an absorbance (A ~ 0.3). The solutions of the compound to be analyzed were degassed under vacuum to remove dissolved oxygen by freeze-pump-thaw cycle. The sample tubes were quickly frozen in liquid nitrogen and quickly transferred into a pre-cooled spectrometer probe at 80 K, followed by temperature equilibration. Using the Elexsys Burker EPR spectrometer with a dielectric cavity in an Oxford CF935 cryostat, having optical access to irradiate the sample with a pulsed laser (Quantel Nd:YAG with 2ω and 3ω modules; 5 ns duration) to analyze the compounds. Matlab based EasySpin software were used to fit TREPR spectra.[3,4]

1.7. Density Functional Theory (DFT) Calculations. The geometries of the compounds were optimized using density functional theory (DFT) with the CAM-B3LYP functional and 6–31G(d) basis set. There are no imaginary frequencies for all optimized structures. The triplet excited state energy of the compounds of the molecules were computed by TDDFT method based on optimized ground state geometries. On the basis of the optimization of the ground state configurations by CAM-B3LYP/6-31G

(d) level, the spin density surfaces of the compounds were computed (Model: IEFPCM). The spin density surfaces at the optimized triplet state geometries were visualized. All these calculations were executed with Gaussian 16.[5]

The ISC rate contributions for the transitions: $S_n \rightarrow T_m$ are given by [6,7]

$$k_{ISC} = \frac{2\pi}{\hbar}\rho(FC)\cdot k_{tot}$$

where $k_{tot} = \sum_{i=X,Y,Z} |\langle T|H_{SO}|S\rangle|_i^2$ and $\rho(FC)$ is the Franck-Condon density of states which contains a factor $\delta(E_T - E_S)$.

1.8 Singlet Oxygen Quantum Yields. 1,3-Diphenylisobenzofuran (DPBF) is a chemical probe that can undergo a specific addition reaction with $^1O_2$, resulting in a decrease in the absorbance of DPBF at 414 nm. To determine the singlet oxygen quantum yield (Φ_Δ), a relative method was used according to (Eq. S1):

$$\Phi_{\text{sam}} = \Phi_{\text{std}}\left(\frac{1-10^{-A}\text{std}}{1-10^{-A}\text{sam}}\right)\left(\frac{m_{\text{sam}}}{m_{\text{std}}}\right)\left(\frac{\eta_{\text{sam}}}{\eta_{\text{std}}}\right)^2$$

where 'sam' and 'std' represent the sample and the standard, Φ and A represent the singlet oxygen quantum yield, excitation wavelength in the absorbance spectrum, m and η represent the slope of the absorbance of DPBF changing over time, and the refractive index of the solvent used for measurement, respectively.

2. NMR and HRMS Spectra of compounds

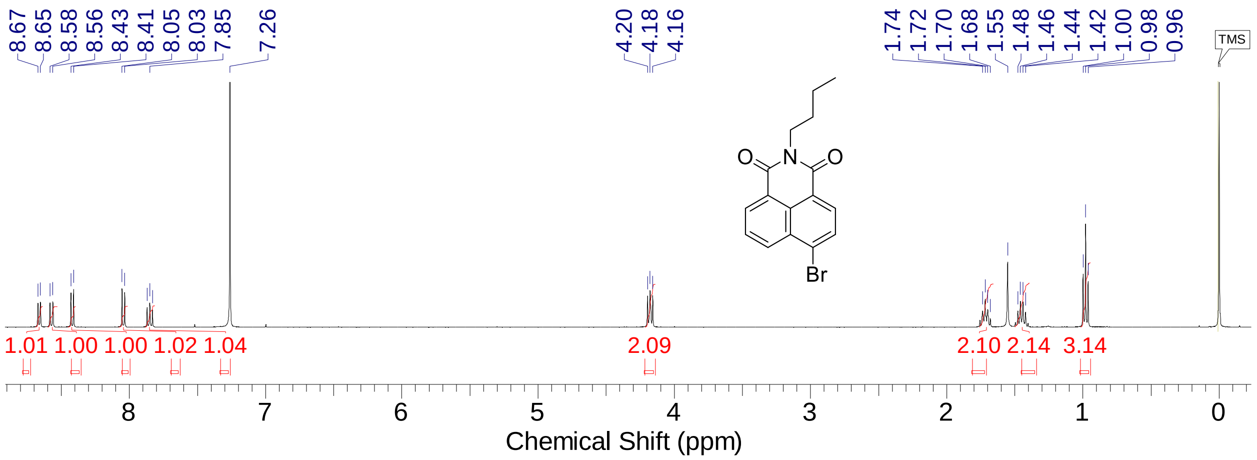

Figure S1. ^{1}H NMR spectra of **NI-Br** (400 MHz, $CDCl_3$). 25 °C.

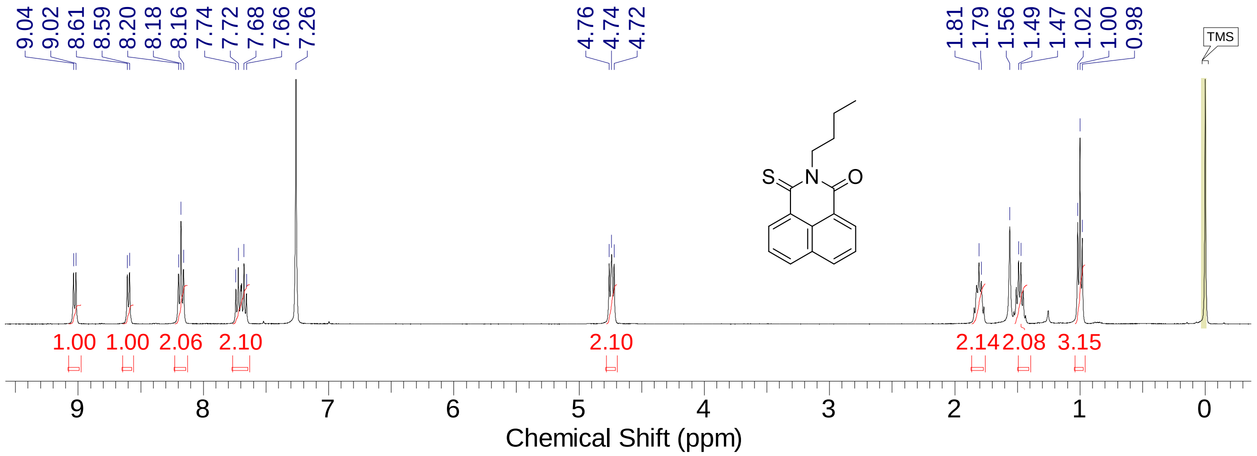

Figure S2. ^{1}H NMR spectra of **S-NI** (400 MHz, $CDCl_3$). 25 °C.

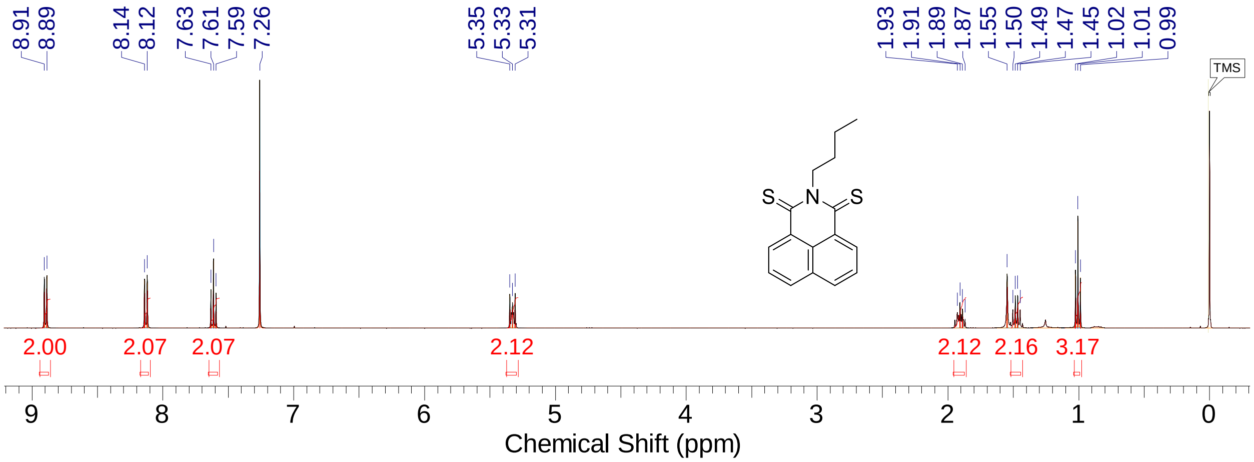

Figure S3. ^{1}H NMR spectra of **DS-NI** (400 MHz, $CDCl_3$). 25 °C.

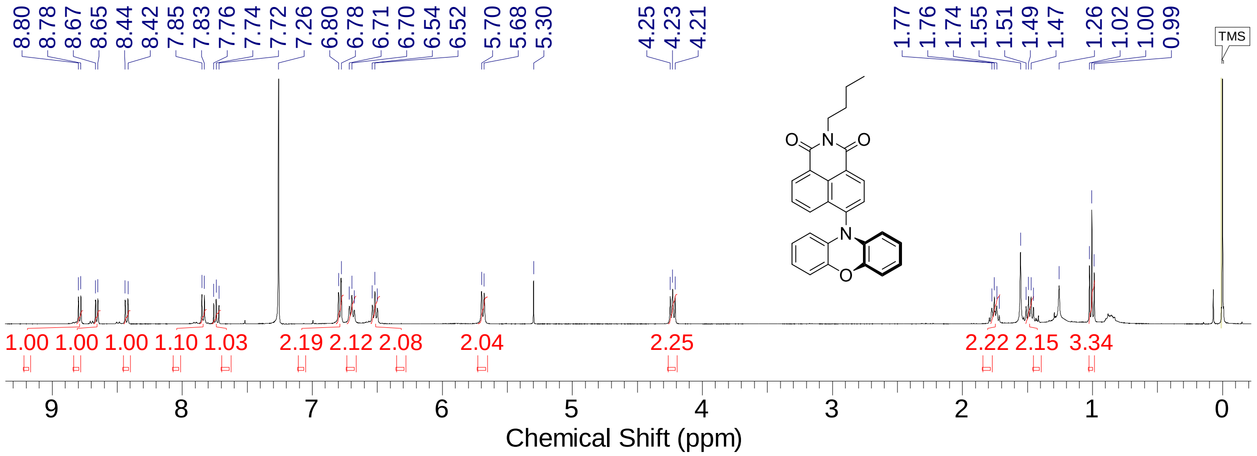

Figure S4. ^{1}H NMR spectra of **NI-PXZ** (400 MHz, $CDCl_3$). 25 °C.

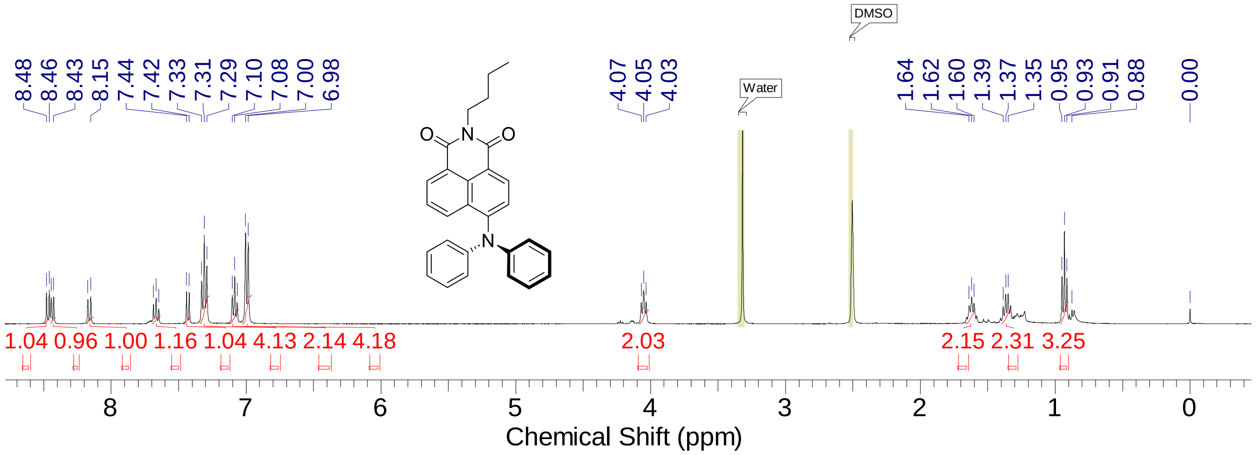

Figure S5. ^{1}H NMR spectra of **NI-DPA** (400 MHz, $CDCl_3$). 25 °C.

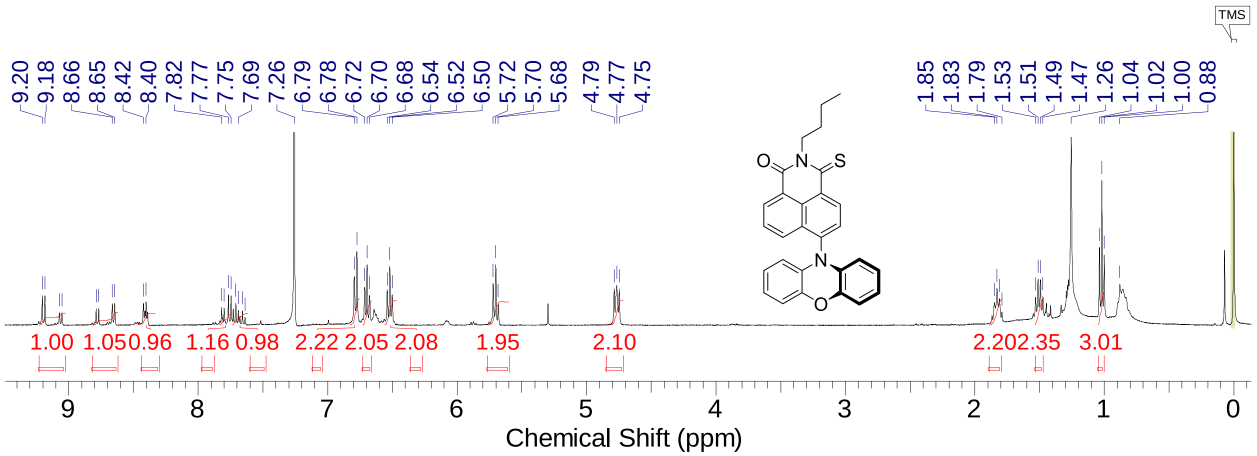

Figure S6. ^{1}H NMR spectra of **S-NI-PXZ** (400 MHz, $CDCl_3$). 25 °C.

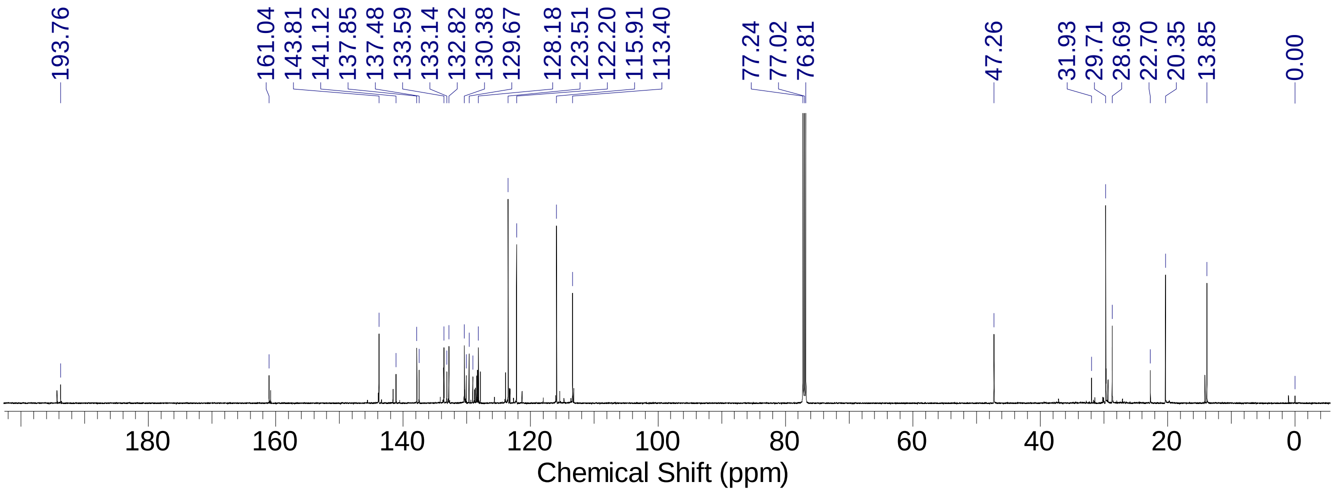

Figure S7. ^{13}C NMR spectra of **S-NI-PXZ** (125 MHz, $CDCl_3$). 25 °C.

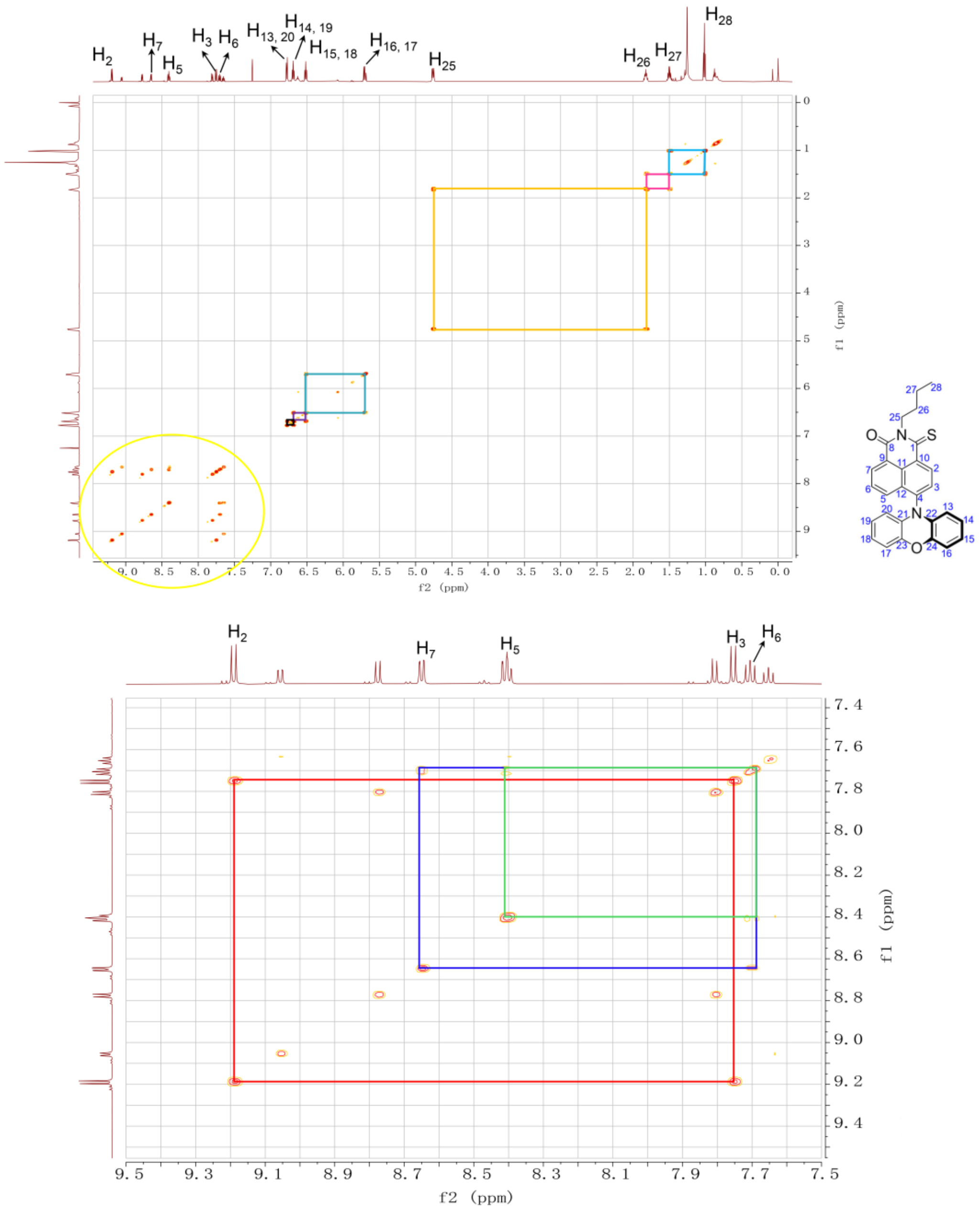

Figure S8. ^{1}H - ^{1}H COSY spectra of compound **S-NI-PXZ**, the data circled in yellow is magnified, 25°C.

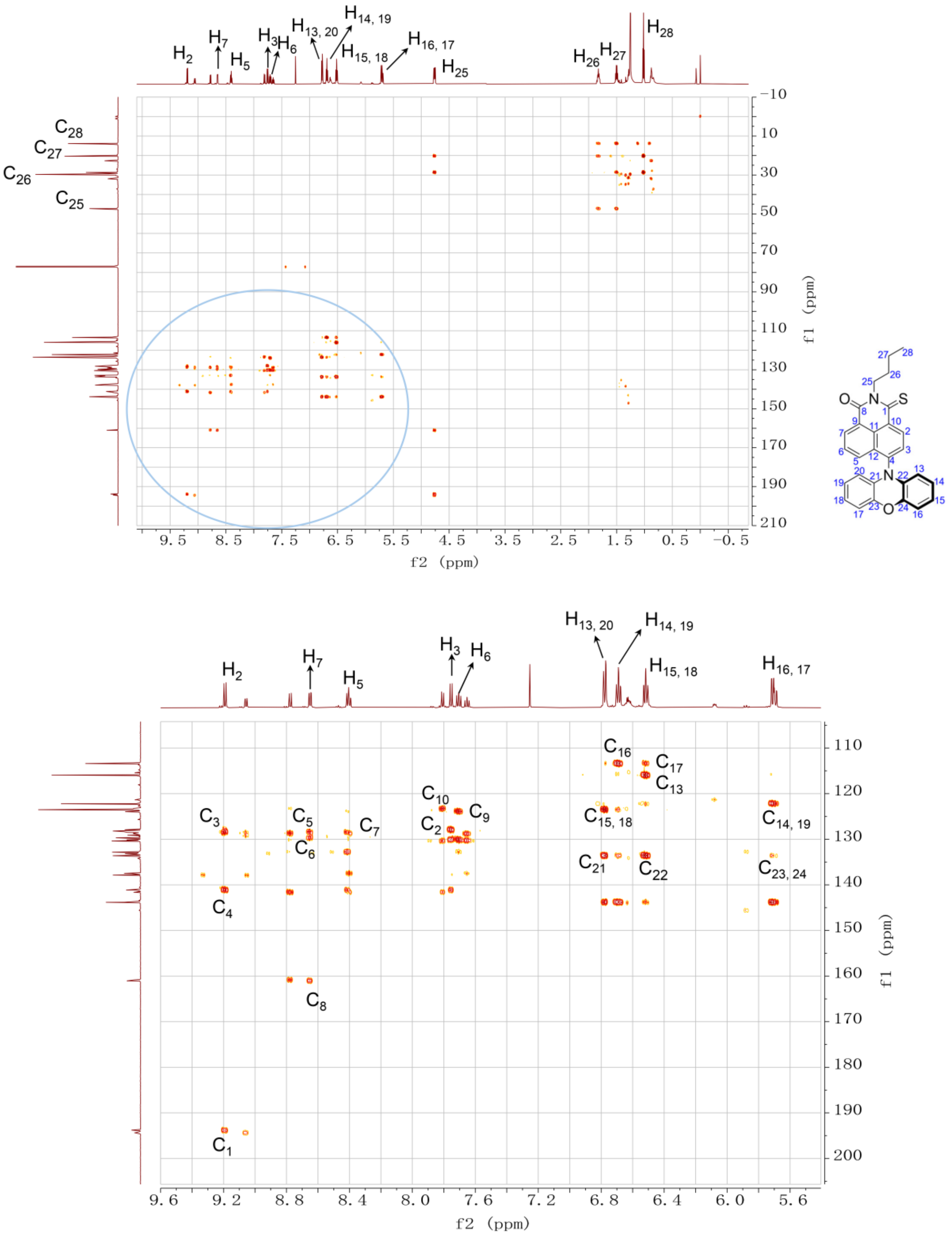

Figure S9. ^{1}H - ^{13}C HMBC spectra of compound **S-NI-PXZ**, the data in the blue circle is magnified, 25°C.

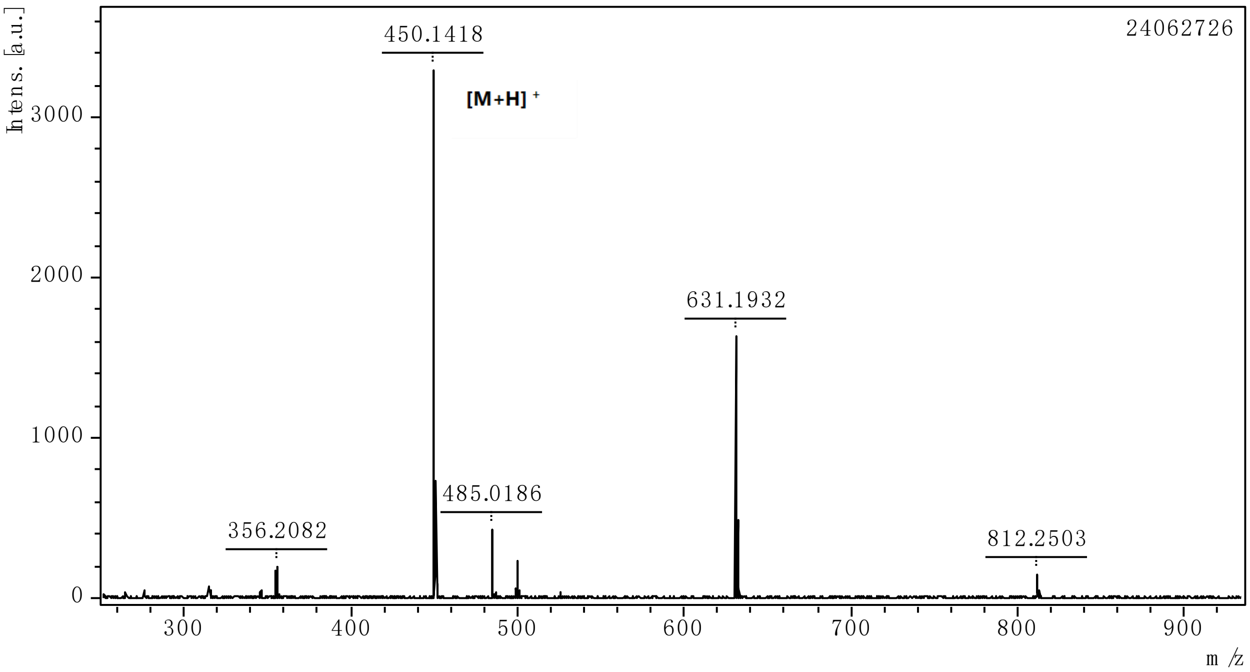

Figure S10. MALDI–TOF–HRMS of compound **S-NI-PXZ**, 25°C.

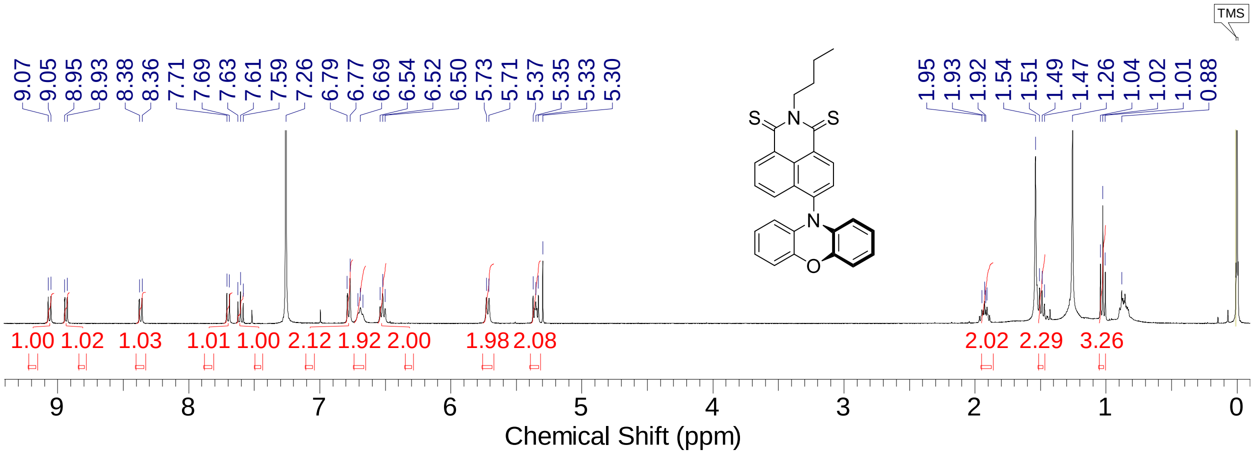

Figure S11. ^{1}H NMR spectra of **DS-NI-PXZ** (400 MHz, $CDCl_3$). 25 °C.

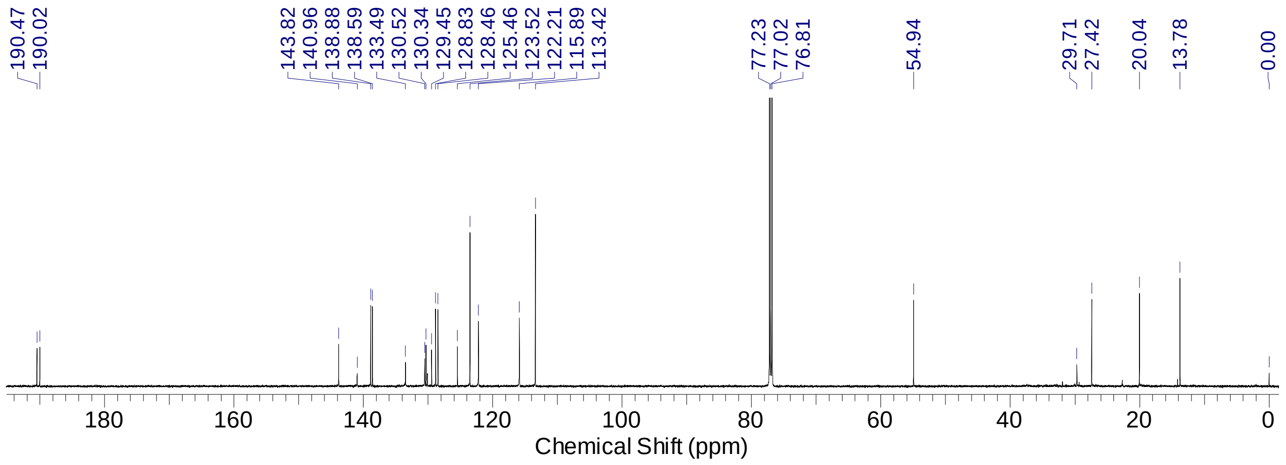

Figure S12. ^{13}C NMR spectra of **DS-NI-PXZ** (125 MHz, $CDCl_3$). 25 °C.

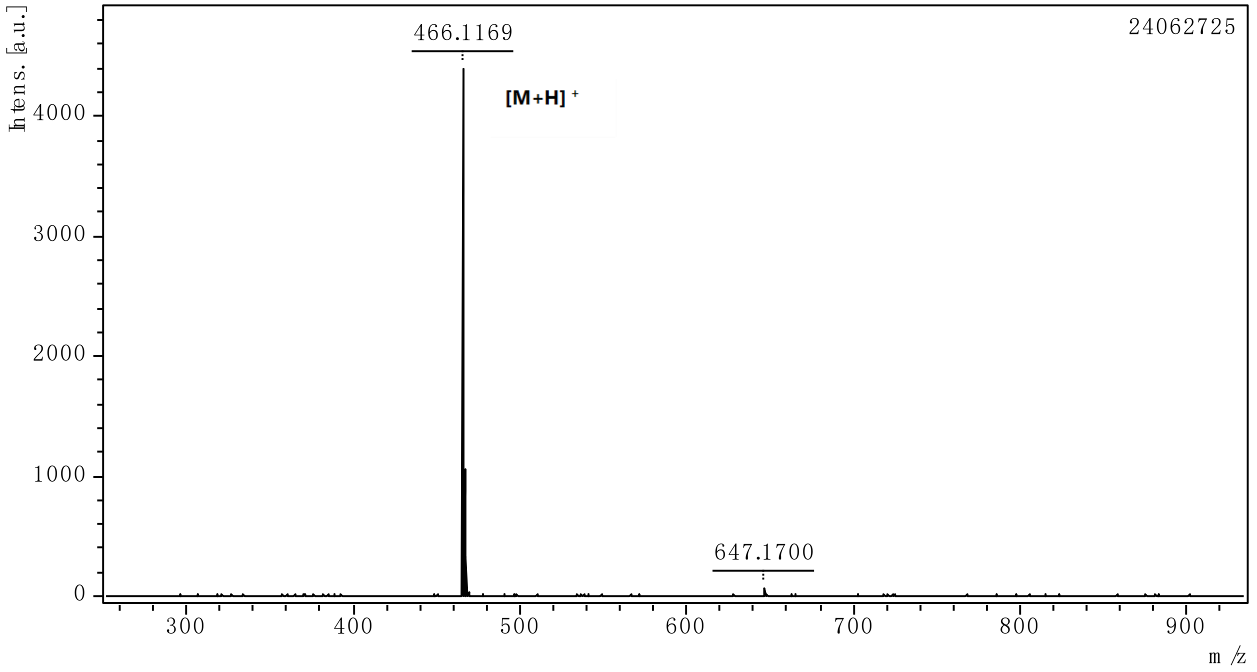

Figure S13. MALDI–TOF–HRMS of compound **DS-NI-PXZ**, 25°C.

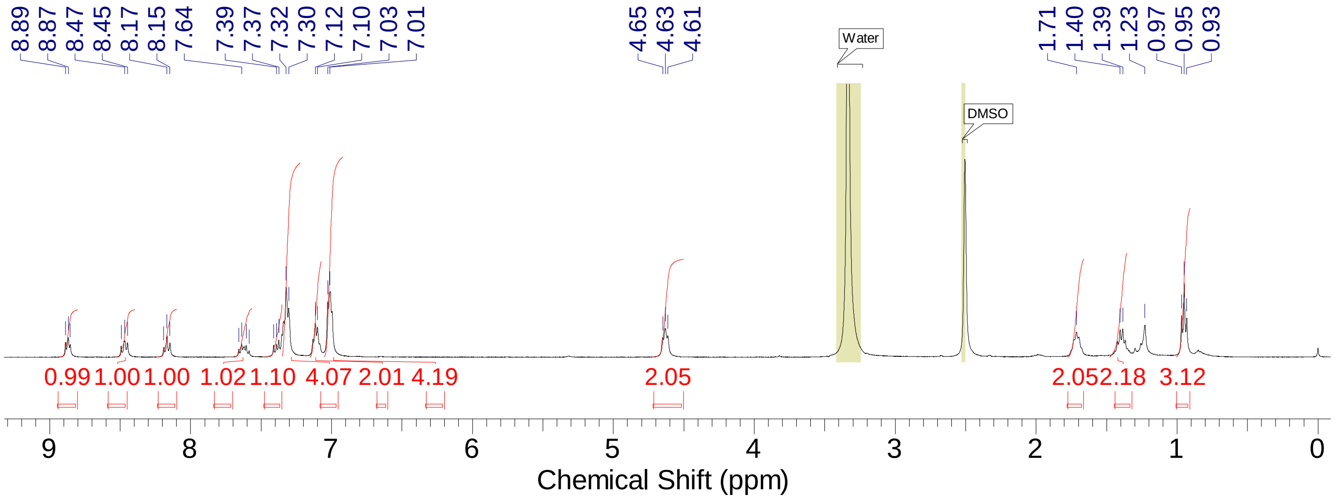

Figure S14. ^{1}H NMR spectra of **S-NI-DPA** (400 MHz, DMSO-d_6). 25 °C.

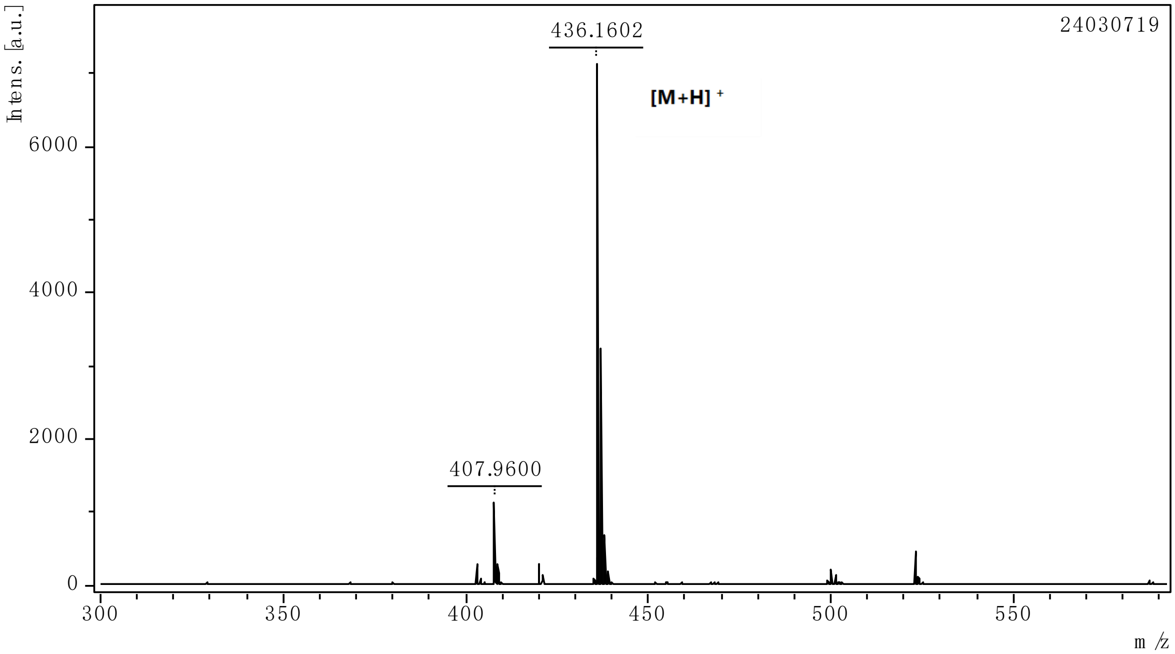

Figure S15. MALDI–TOF–HRMS of compound **S-NI-DPA**, 25°C.

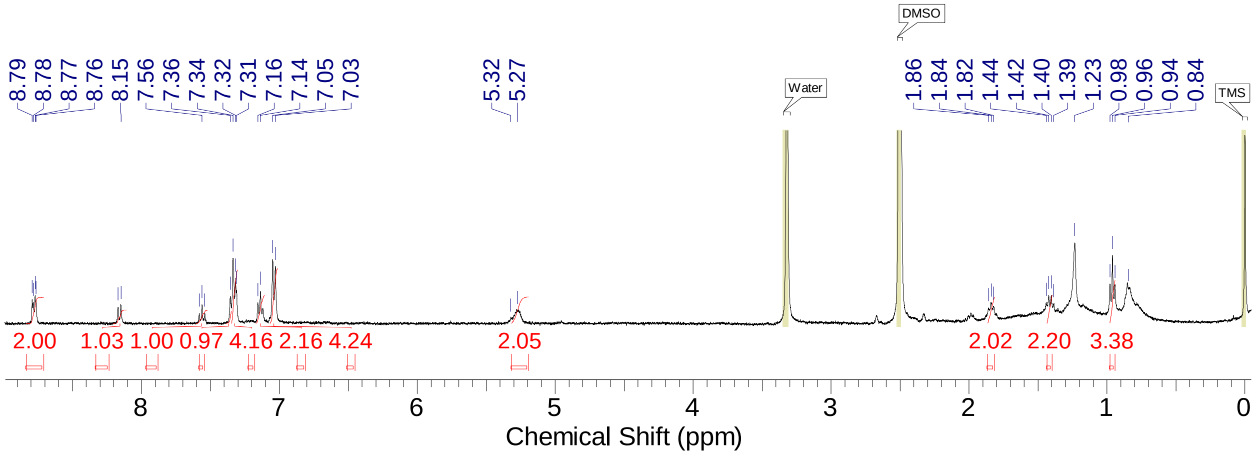

Figure S16. ^{1}H NMR spectra of **DS-NI-DPA** (400 MHz, DMSO-d_6). 25 °C.

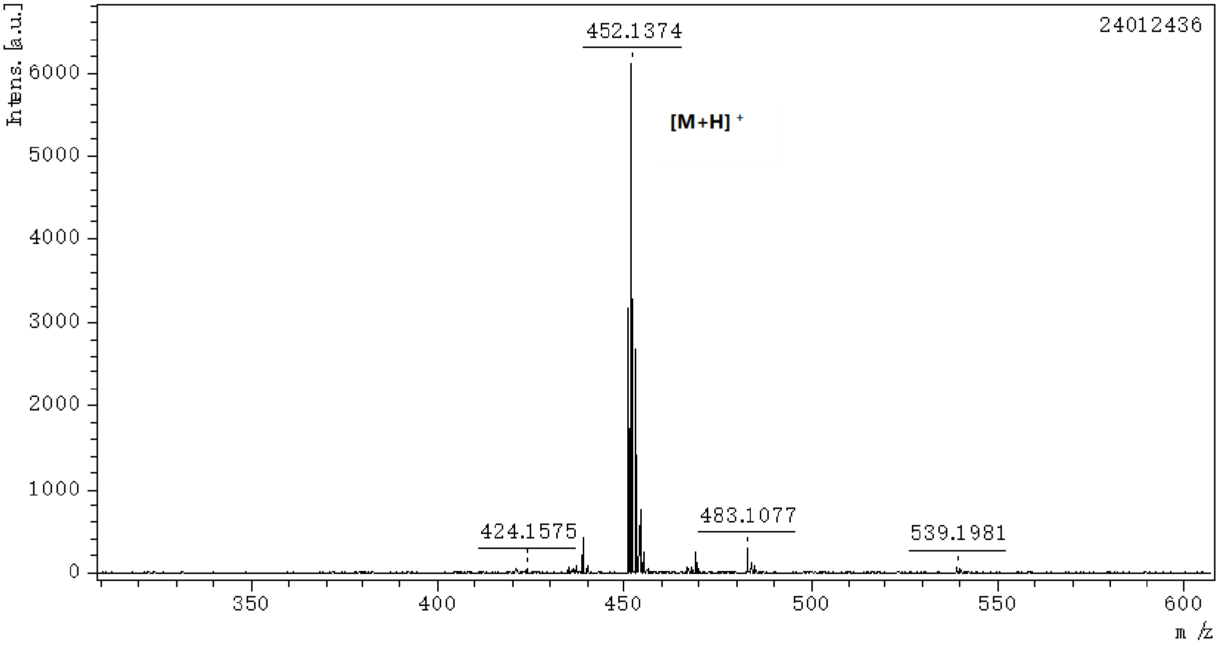

Figure S17. MALDI–TOF–HRMS of compound **DS-NI-DPA**, 25°C.

3. Steady State UV-vis Absorption

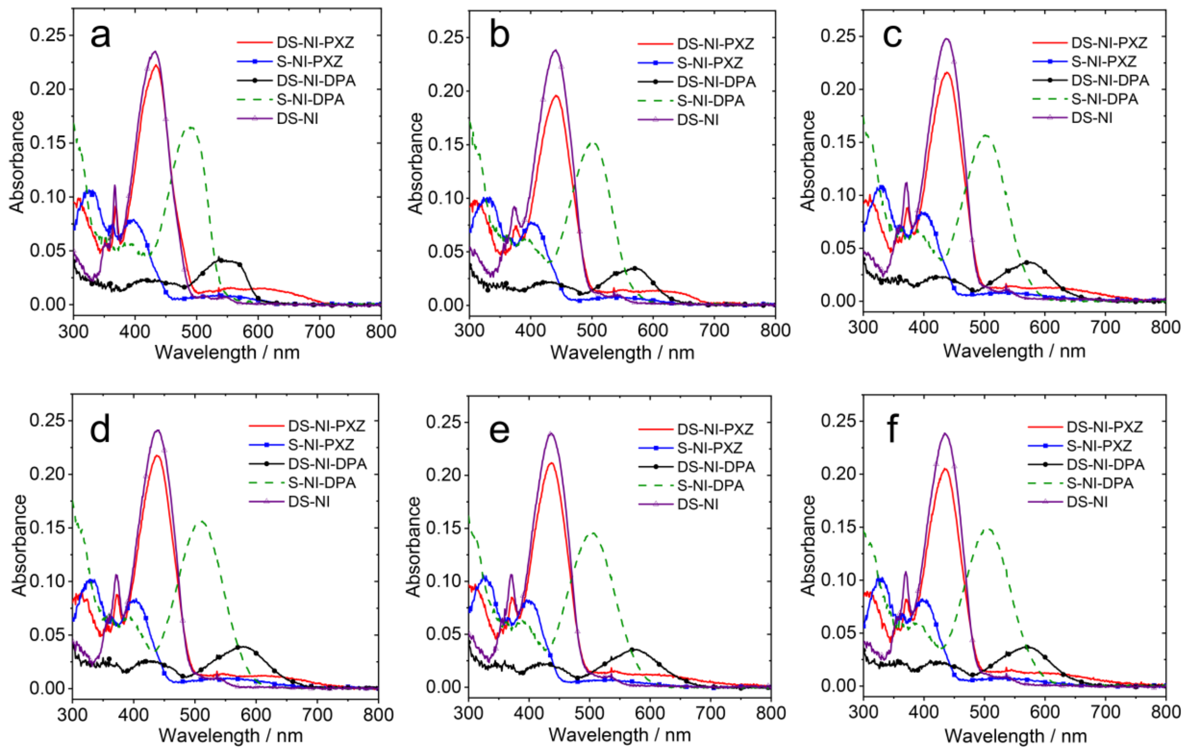

Figure S18. UV–vis absorption spectra of the compounds in (a) HEX, (b) TOL, (c) THF, (d) DCM, (e) ACN, (f) MeOH, $c = 1.0 \times 10^{-5}$ M, 20 °C.

4. Fluorescence Emission and Fluorescence Lifetime Spectra

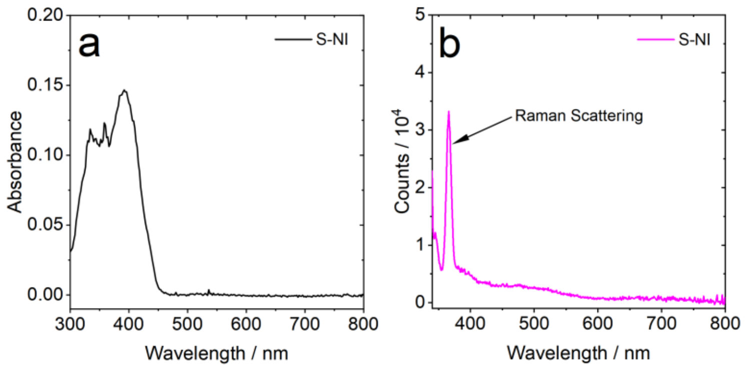

Figure S19. (a) UV–vis absorption spectra of **S-NI** in *n*-HEX. (b) Fluorescence emission spectra of **S-NI** in *n*-HEX. Optically matched solutions were used (A = 0.100 at λ_{ex} = 330 nm), 20 °C.

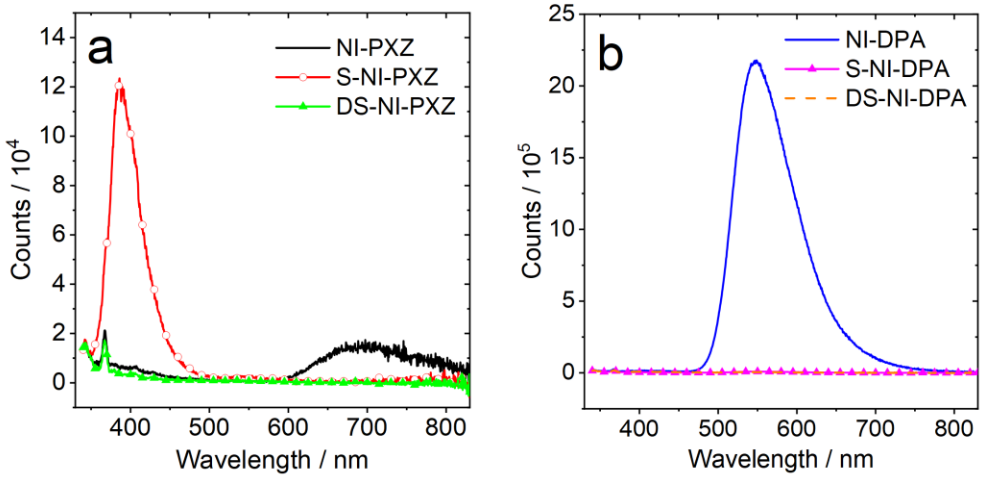

Figure S20. Comparison of the fluorescence spectra of (a) **NI-PXZ, S-NI-PXZ** and **DS-NI-PXZ** (b) **NI-DPA, S-NI-DPA** and **DS-NI-DPA** in TOL. Optically matched solutions were used (A = 0.100 at λ_{ex} = 330 nm), 20 °C.

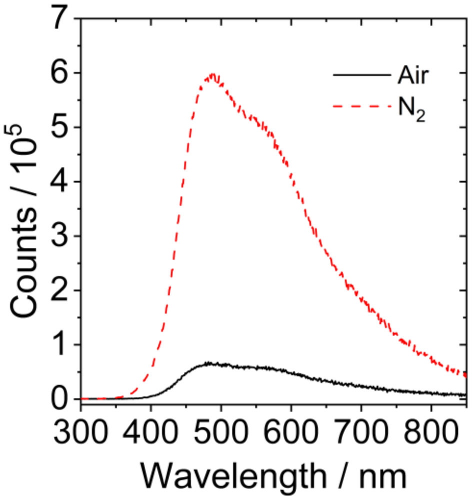

Figure S21. Fluorescence spectra of **NI-PXZ** in *n*-HEX, $c = 1.0 \times 10^{-5}$ M, λ_{ex} = 340 nm, 25 °C.

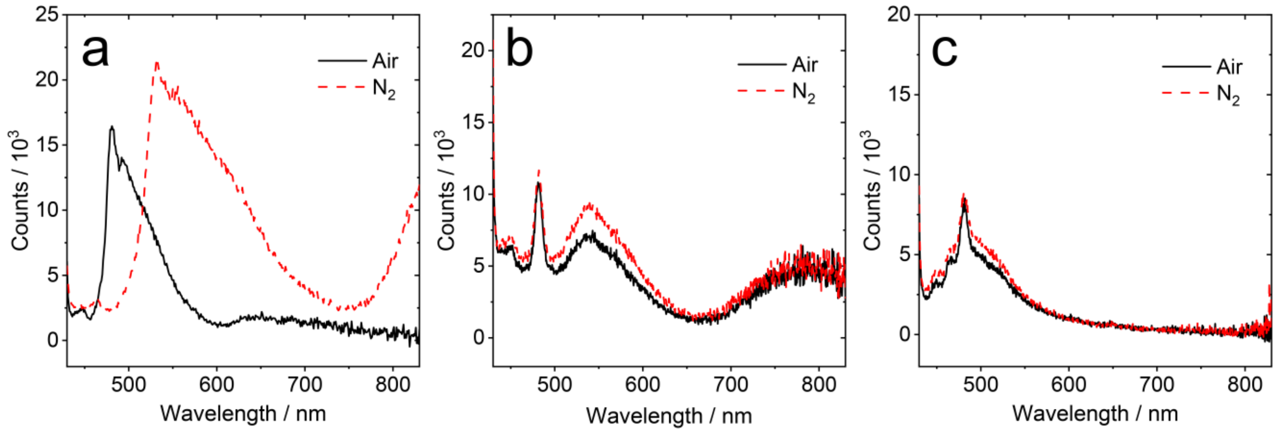

Figure S22. Fluorescence spectra of **S-NI-PXZ** under different atmospheres (N_2, Air), in (a) HEX, (b) TOL and (c) ACN, c = 1.0 × 10^{-5} M, λ_{ex} = 420 nm, 20 °C.

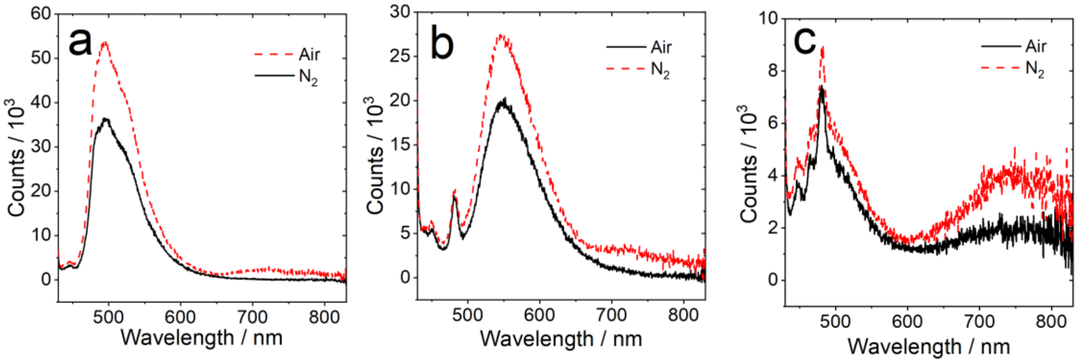

Figure S23. Fluorescence spectra of **S-NI-DPA** under different atmospheres (N_2, Air), in (a) HEX, (b) TOL and (c) ACN, c = 1.0 × 10^{-5} M, λ_{ex} = 420 nm, 20 °C.

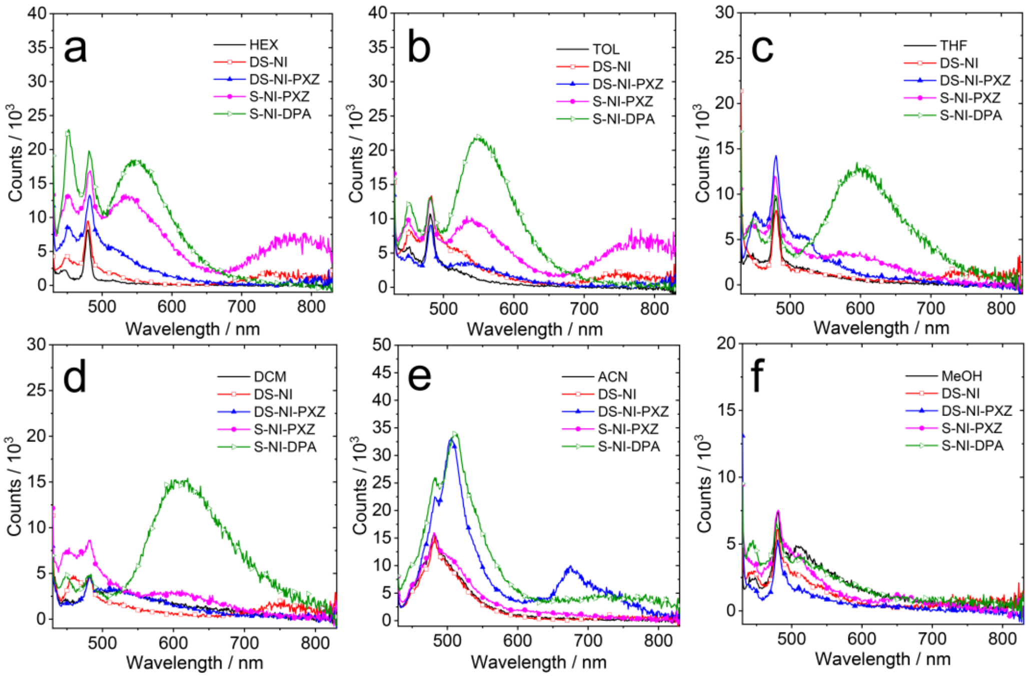

Figure S24. Fluorescence emission spectra of **DS-NI**, **DS-NI-PXZ**, **S-NI-PXZ** and **S-NI-DPA** in (a) HEX, (b) TOL, (c) THF, (d) DCM, (e) ACN, (f) MeOH. Optically matched solutions were used in each panel (each of the solutions gives the same absorbance at the excitation wavelength), λ_{ex} = 420 nm, A = 0.10, 20 °C.

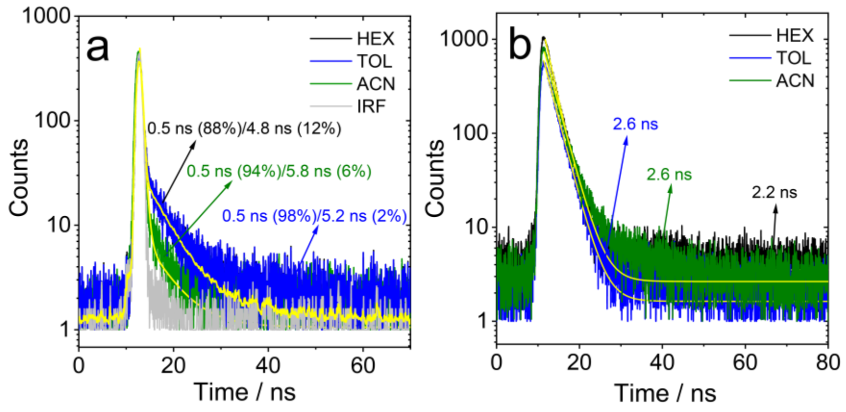

Figure S25. Fluorescence decay traces of (a) **S-NI** under different solvents were monitored at 366 nm and (b) **S-NI-PXZ** under different solvents were monitored at 410 nm. λ_{ex} = 340 nm, $c = 1.0 \times 10^{-5}$ M 25 °C.

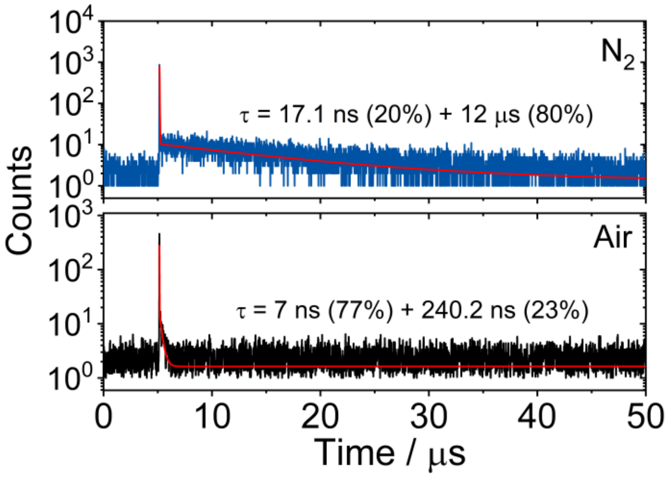

Figure S26. Fluorescence decay traces of **NI-PXZ** under different atmospheres (N_2, Air). λ_{ex} = 340 nm, decay trace at 580 nm, excited with nanosecond EPLED (340 nm), $c = 1.0 \times 10^{-5}$ M in *n*-HEX, 25 °C.

5. Low-temperature Luminescence Spectra and Lifetime Studies

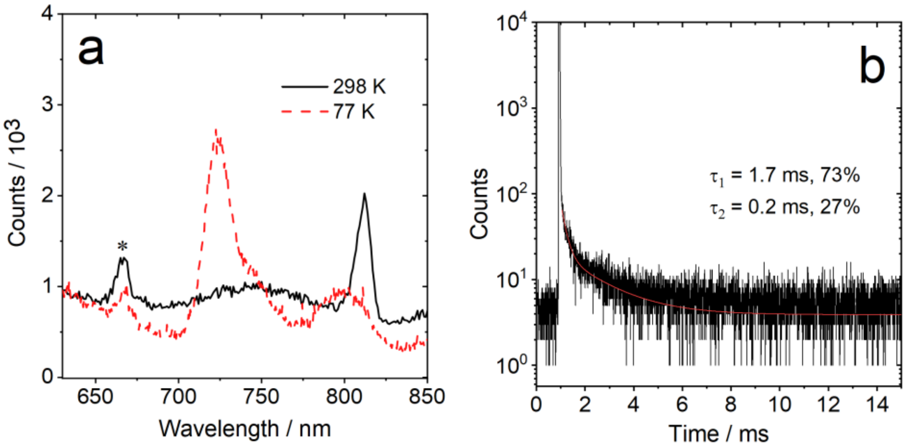

Figure S27. (a) Phosphorescence spectra of **DS-NI** at room temperature and low-temperature. (b) Phosphorescence lifetime of **DS-NI** monitor at 725 nm at 77 K. (λ_{ex} = 445 nm, $c = 1.0 \times 10^{-4}$ M in deaerated 2-MeTHF). The band marked with asterisk stands for the background photoluminescence of the solvent of 2-MeTHF at 77 K.

6. Singlet Oxygen Quantum (Φ_Δ) Yields

Table S1. Singlet oxygen quantum (Φ_Δ) yields of thionated derivatives in different solvents.

	HEX	TOL	DCM	ACN
**NI-DPA** [a]	– [d]	45	26	– [d]
**NI-PXZ** [c]	7	1	– [d]	– [d]
**DS-NI** [a]	– [d]	46	7	– [d]
**DS-NI-PXZ** [a]	21	24	– [d]	– [d]
**DS-NI-DPA** [b]	50	43	70	48
**S-NI-DPA** [b]	80	87	100	100
**S-NI-PXZ** [b]	10	– [d]	– [d]	– [d]

[a] λ_{ex} = 430 nm. $Ru(bpy)_3[PF_6]_2$ was used as standard compound (Φ_Δ = 57% in DCM). [b] λ_{ex} = 400 nm. $Ru(bpy)_3[PF_6]_2$ was used as standard compound (Φ_Δ = 57% in DCM). [c] Literature data. [d] Not observed.

7. Electrochemical Study, Spectroelectrochemistry and the Chemical Reduction Study

$$E_{CS} = e[E_{OX} - E_{RED}] + \Delta G_S$$

$$\Delta G_S = -\frac{e^2}{4\pi\varepsilon_S\varepsilon_0 R_{CC}} - \frac{e^2}{8\pi\varepsilon_0}\left(\frac{1}{R_D} + \frac{1}{R_A}\right)\left(\frac{1}{\varepsilon_{REF}} - \frac{1}{\varepsilon_S}\right)$$

$$\Delta G^0{}_{CS} = e[E_{OX} - E_{RED}] - E_{00} + \Delta G_S$$

where ΔG_S is the static Coulombic energy. Among the Weller equation, e stands for electronic charge. E_{OX} stands for half-wave potential for one-electron oxidation of the electron-donor unit and E_{RED} is half-wave potential for one-electron reduction of the electron-acceptor unit. ε_S = static dielectric constant of the solvent, R_{CC} = center-to-center separation distance between the electron donor and acceptor, determined by results from theoretical calculations for structure optimization. R_D and R_A are the radius

of the electron donor and acceptor, respectively. ε_{REF} is the static dielectric constant of the solvent used for the electrochemical studies, ε_0 is permittivity of vacuum. The solvents used in the calculations for free energy changes of the electron transfer process are *n*-HEX (ε_S = 1.88), toluene (ε_S = 2.38), dichloromethane (ε_S = 8.93) and acetonitrile (ε_S = 20.7).

Table S2. Electrochemical Redox Potentials of the Compounds [a]

Compounds	E_{OX} (V)	E_{RED} (V)
**S-NI**	–[b]	–1.40
**DS-NI**	– [b]	–1.10
**NI-PXZ**	+0.41	–1.60
**NI-DPA**	+0.52	–1.68

[a] Redox potentials (vs. Fc^+/ Fc) of the compounds in N_2–saturated ACN. [b] Not applicable.

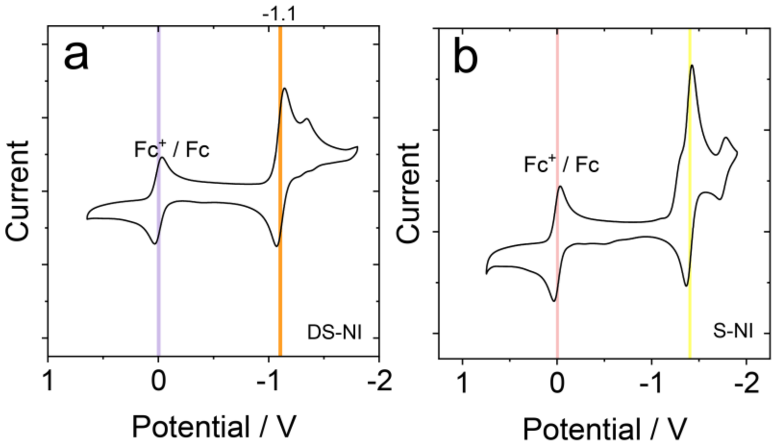

Figure S28. Cyclic voltammograms of the compounds (a) **DS-NI** and (b) **S-NI** in deaerated ACN containing 0.10 M tetrabutylammonium hexafluorophosphate ($Bu_4N[PF_6]$) as the supporting electrolyte and Ag/$AgNO_3$ as the reference electrode. Scan rate: 50 mV/s. Ferrocenium/ferrocene (Fc^+/Fc) redox couple was used as the internal reference, 25 °C.

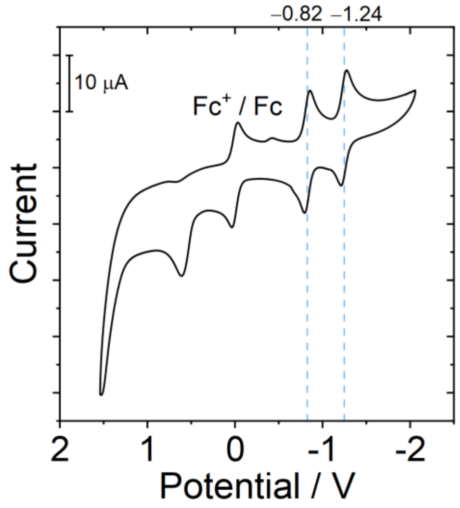

Figure S29. Cyclic voltammograms of the 1,1′-dimethyl-4,4′-bipyridinium (MV^{2+}) in deaerated ACN containing 0.10 M tetrabutylammonium hexafluorophosphate ($Bu_4N[PF_6]$) as the supporting electrolyte and Ag/$AgNO_3$ as the reference electrode. Scan rate: 50 mV/s. Ferrocenium/ferrocene (Fc^+/Fc) redox couple was used as the internal reference, 20 °C.

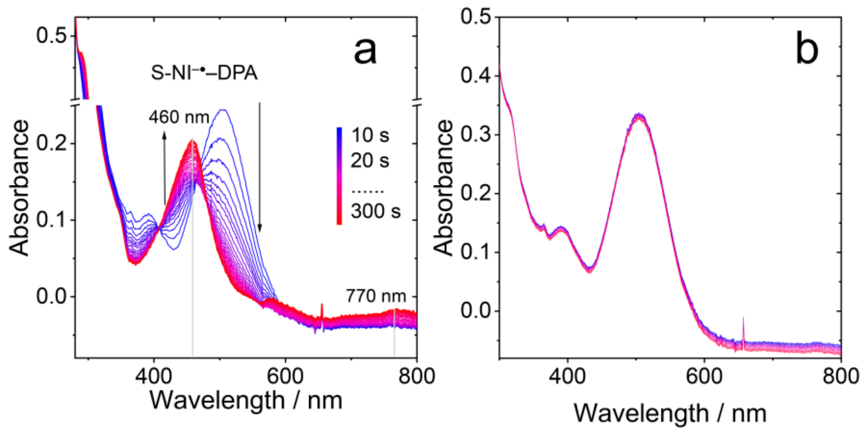

Figure S30. S-NI-DPA (a) reduction with an applied potential of −1.43 V and (b) oxidation with an applied potential of +0.30 V on the working electrode. Ag/$AgNO_3$ was used as reference electrode. The spectra were recorded in situ with a spectroelectrochemical cuvette (1 mm optical path). *c*[**S-NI-DPA**] = 5.0×10^{-5} M in deaerated ACN. 25 °C.

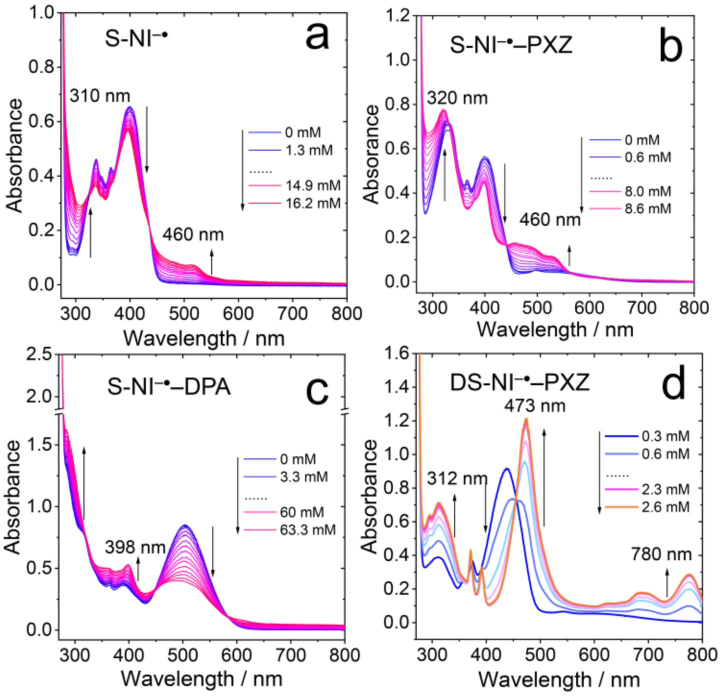

Figure S31. (a) **S-NI**, (b) **S-NI-PXZ**, (c) **S-NI-DPA,** and (d) **DS-NI-PXZ** chemically reduced with tetrabutylammonium fluoride (TBAF) to generate [S-NI]$^{-•}$ and [DS-NI]$^{-•}$ in deaerated ACN. *c*[**S-NI-PXZ**] = 6.0 × 10^{-5} M, *c*[**DS-NI-PXZ, S-NI**] = 4.0 × 10^{-5} M, *c*[**S-NI-DPA**] = 5.0 × 10^{-5} M, *c*[**TBAF**] = 0.2 M, 25 °C.

8. Femtosecond Transient Absorption Spectra

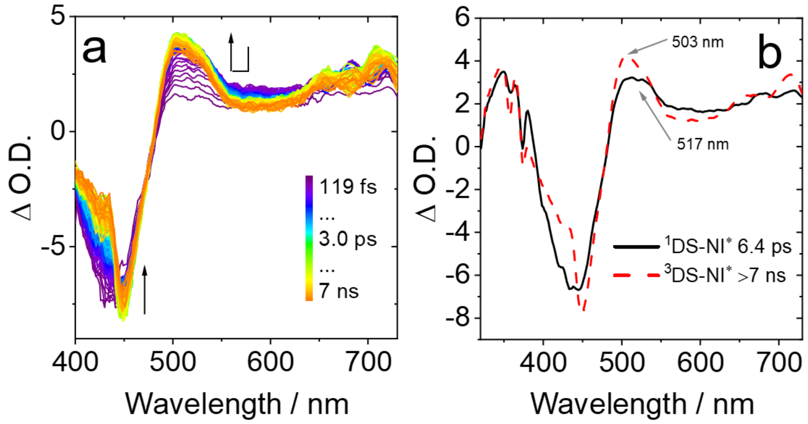

Figure S32. Femtosecond transient absorption (fs-TA) spectra of **DS-NI** in (a) *n*-HEX. The related evolution associated difference spectra (EADS) in (b) *n*-HEX for **DS-NI**. EADS were obtained from target analysis with the sequential model. λ_{ex} = 355 nm. c = 3.0 × 10^{-5} M, 25 °C.

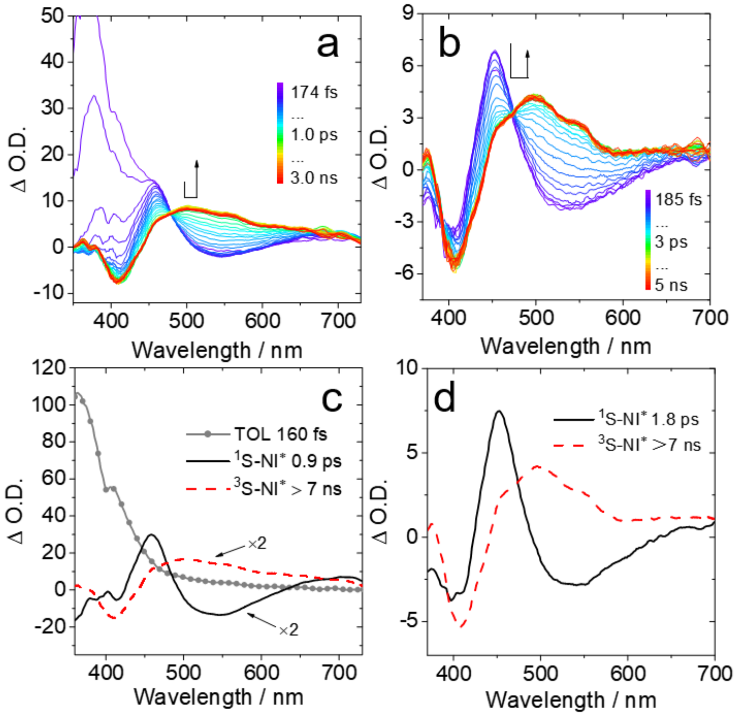

Figure S33. Femtosecond transient absorption (fs-TA) spectra of **S-NI** in (a) TOL, (b) ACN. The related evolution associated difference spectra (EADS) in (c) TOL, (d) ACN for **S-NI**. EADS were obtained from target analysis with the sequential model. λ_{ex} = 355 nm. c = 3.0 × 10^{-5} M, 25 °C.

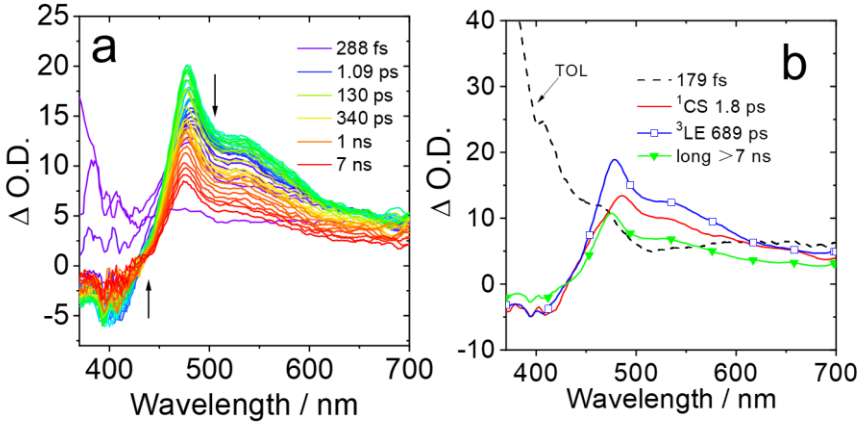

Figure S34. Femtosecond transient absorption (fs-TA) spectra of **S-NI-PXZ** in (a) TOL. The related evolution associated difference spectra (EADS) in (b) TOL for **S-NI-PXZ**. EADS were obtained from target analysis with the sequential model. λ_{ex} = 355 nm. c = 3.0 × 10^{-5} M, 25 °C.

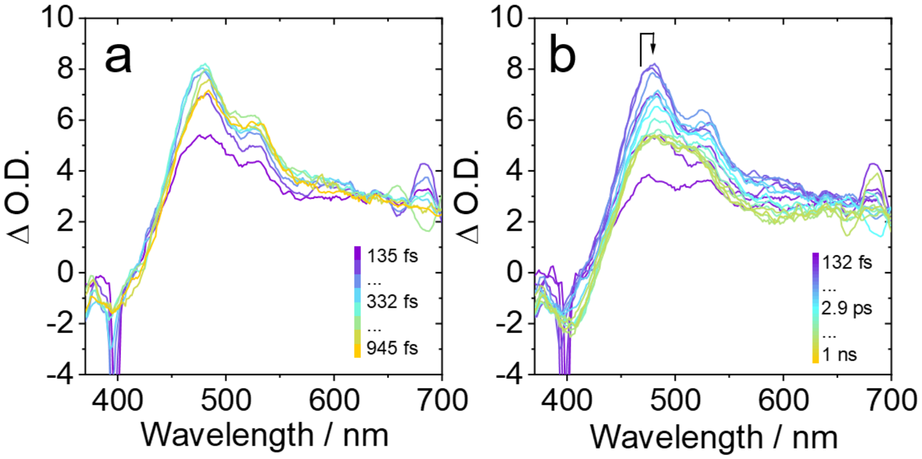

Figure S35. Femtosecond transient absorption (fs-TA) spectra of **S-NI-PXZ** in *n*-HEX. Transient absorption spectra of **S-NI-PXZ** with shorter time windows, (c) 1 ps and (b) 1 ns. Excited at 355 nm.

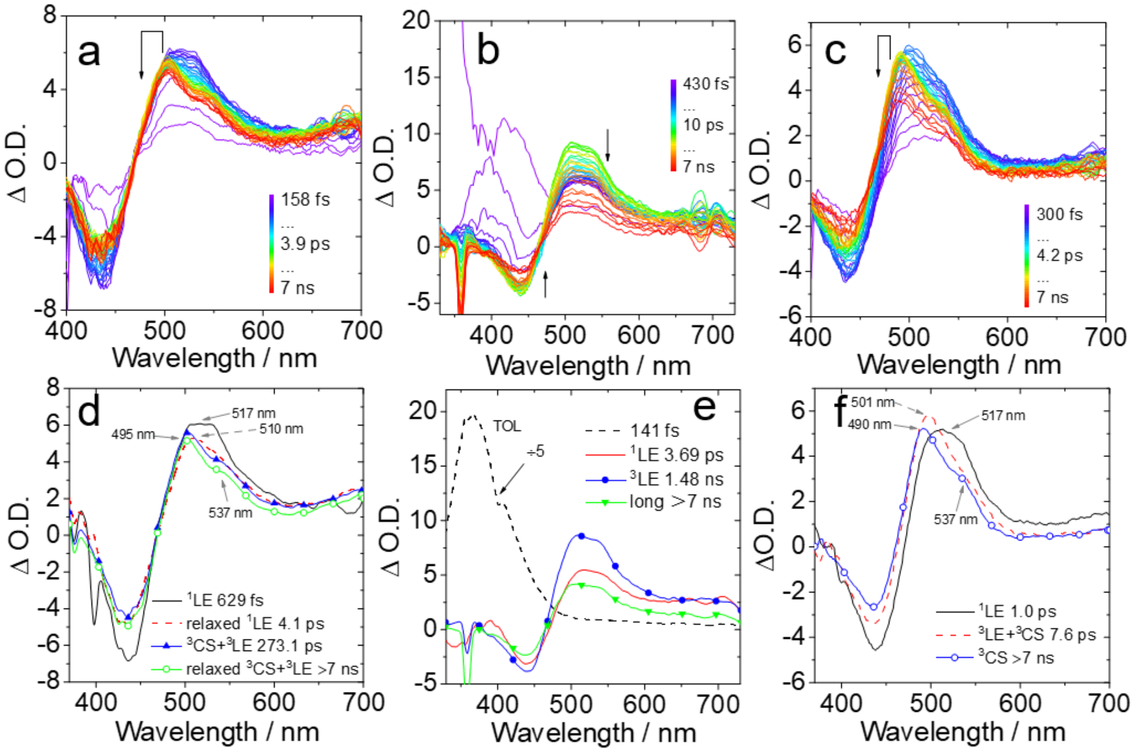

Figure S36. Femtosecond transient absorption (fs-TA) spectra of **DS-NI-PXZ** in (a) HEX, (b) TOL and (c) ACN. The related evolution associated difference spectra (EADS) in (d) HEX, (e) TOL and (f) ACN for **DS-NI-PXZ**. EADS were obtained from target analysis with the sequential model. λ_{ex} = 355 nm, c = 3.0 × 10^{-5} M, 25 °C.

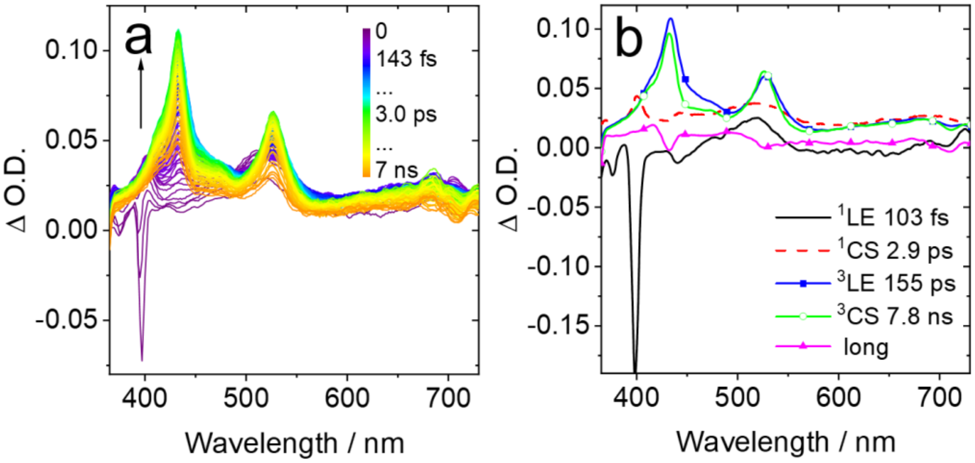

Figure S37. Femtosecond transient absorption (fs-TA) spectra of **NI-PXZ** in (a) *n*-HEX. The related evolution associated difference spectra (EADS) in (b) *n*-HEX for **NI-PXZ**. EADS were obtained from target analysis with the sequential model. λ_{ex} = 355 nm. c = 3.0 × 10^{-5} M, 25 °C.

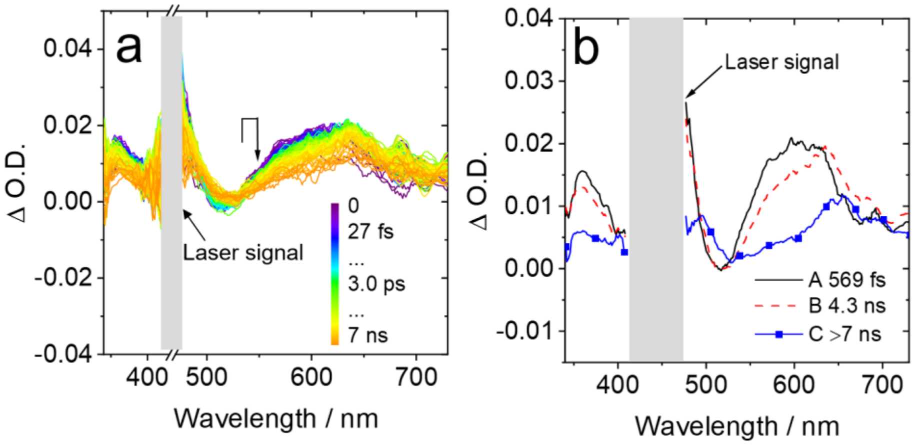

Figure S38. Femtosecond transient absorption (fs-TA) spectra of **NI-DPA** in (a) *n*-HEX. The related evolution associated difference spectra (EADS) in (b) *n*-HEX for **NI-DPA**. EADS were obtained from target analysis with the sequential model. λ_{ex} = 450 nm. c = 3.0 × 10^{-5} M, 25 °C.

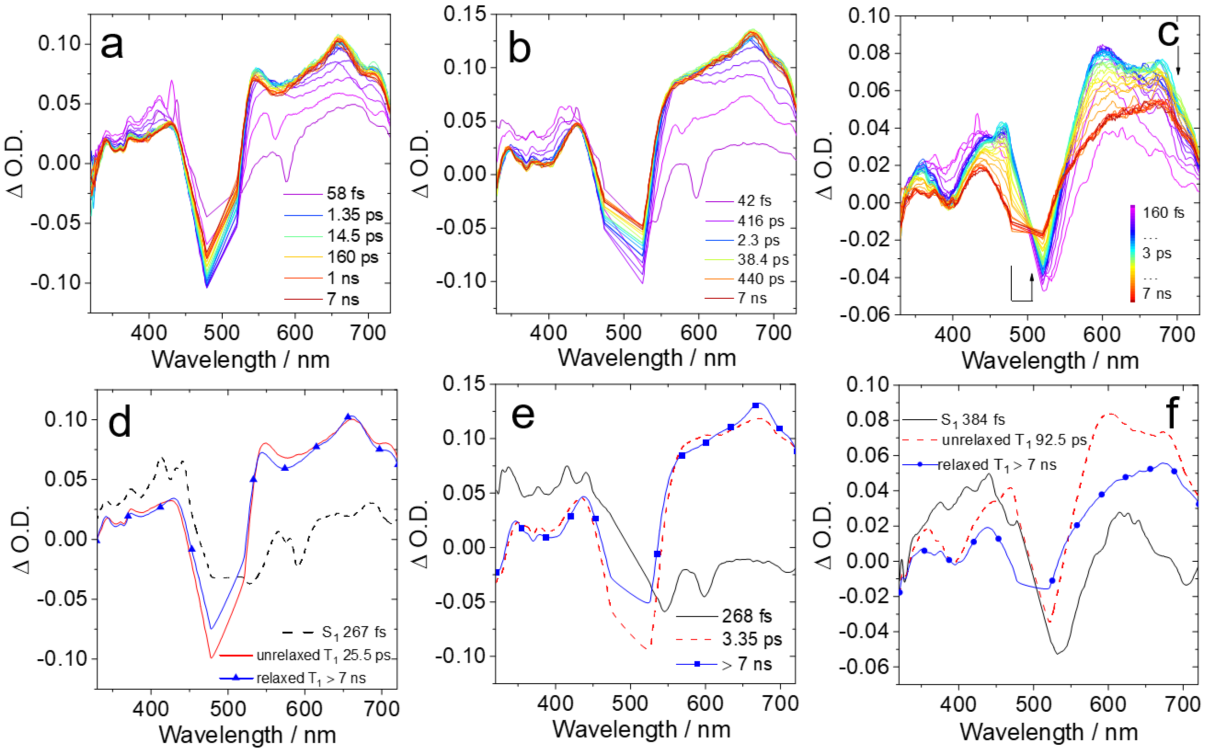

Figure S39. Femtosecond transient absorption (fs-TA) spectra of **S-NI-DPA** in (a) *n*-HEX, (b) TOL and (c) ACN. The related evolution associated difference spectra (EADS) in (d) *n*-HEX, (e) TOL and (f) ACN for **S-NI-DPA**. EADS were obtained from target analysis with the sequential model. λ_{ex} = 500 nm. *c* = 3.0×10^{-5} M, 25 °C.

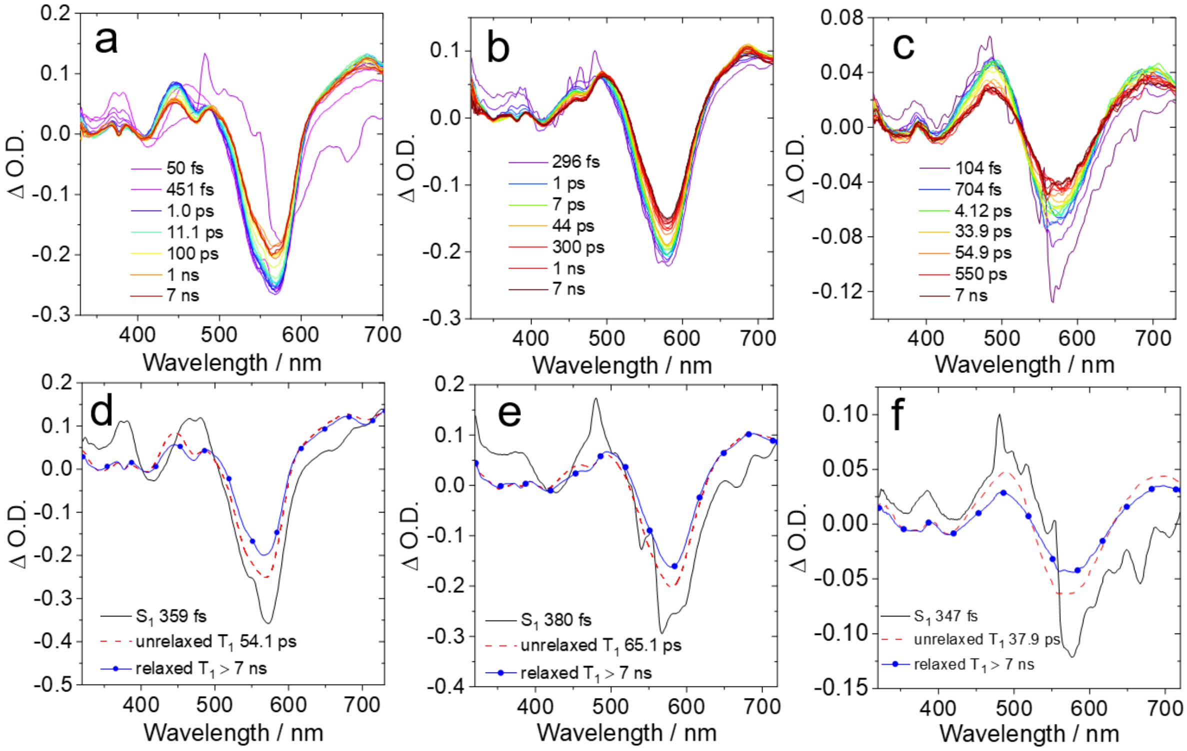

Figure S40. Femtosecond transient absorption (fs-TA) spectra of **DS-NI-DPA** in (a) *n*-HEX, (b) TOL and (c) ACN. The related evolution associated difference spectra (EADS) in (d) *n*-HEX, (e) TOL and (f) ACN for **DS-NI-DPA**. EADS were obtained from target analysis with the sequential model. λ_{ex} = 550 nm. c = 3.0×10^{-5} M, 25 °C.

9. Nanosecond Transient Absorption Spectra

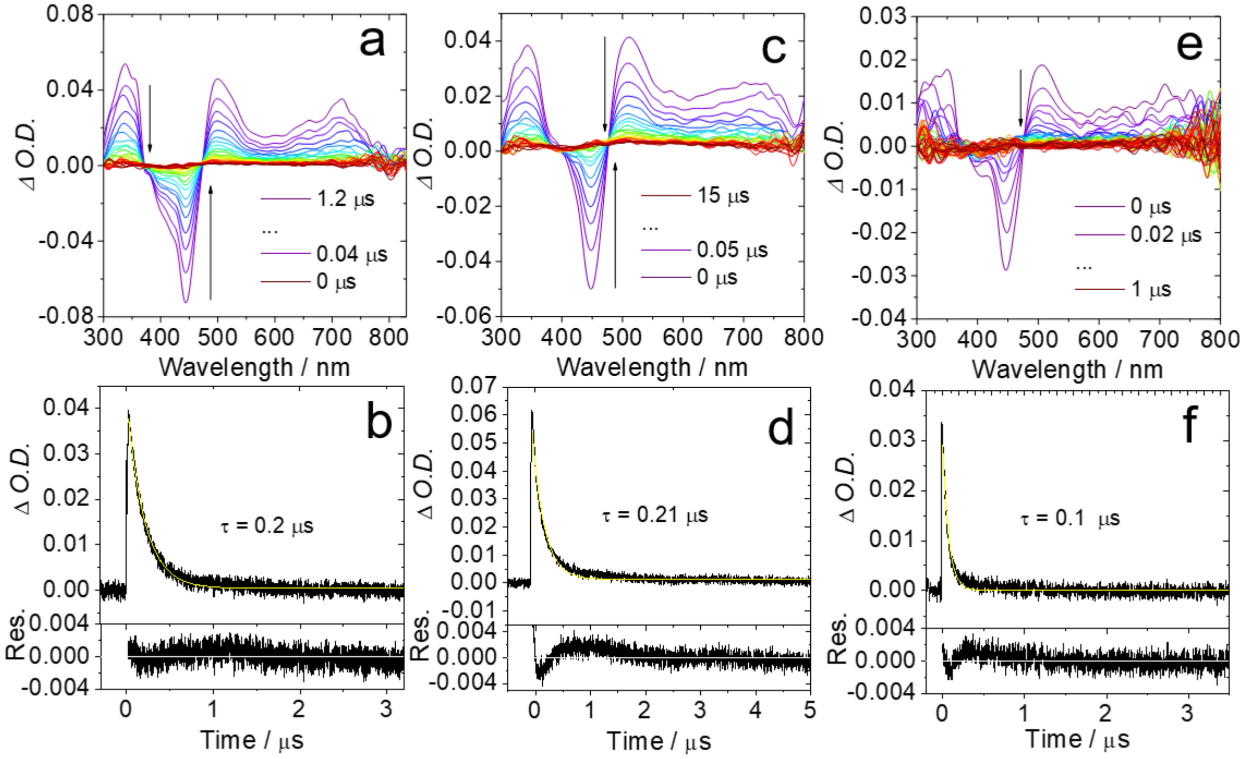

Figure S41. Nanosecond transient absorption spectra of **DS-NI** in deaerated (a) *n*-HEX, (c) TOL and (e) ACN. The decay curves in deaerated (b) *n*-HEX at 510 nm (d) TOL at 510 nm and (f) ACN at 520 nm after pulsed laser excitation at 355 nm, c = 2.0×10^{-5} M. 25 °C.

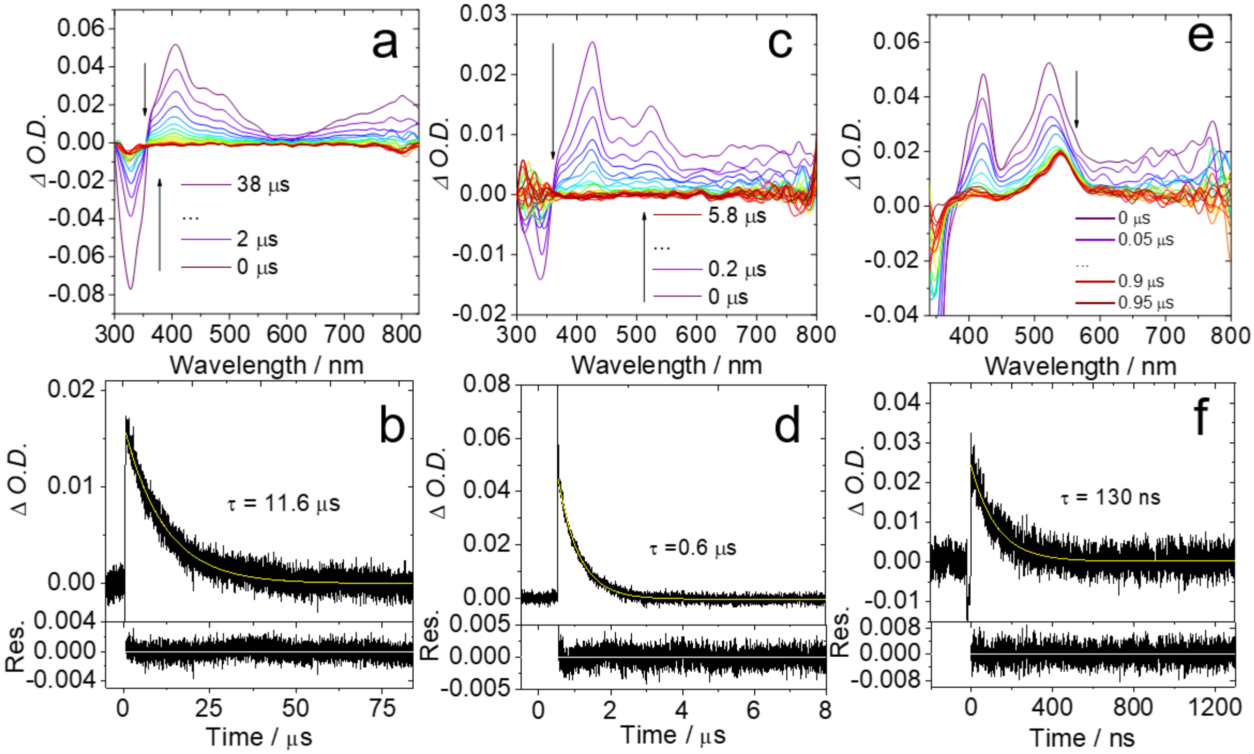

Figure S42. Nanosecond transient absorption spectra of **NI-PXZ** in deaerated (a) *n*-HEX, (c) TOL and (e) ACN. The decay curves in deaerated (b) *n*-HEX at 410 nm (d) TOL at 420 nm and (f) ACN at 430 nm after pulsed laser excitation at 355 nm, c = 3.0 × 10^{-5} M. 25 °C.

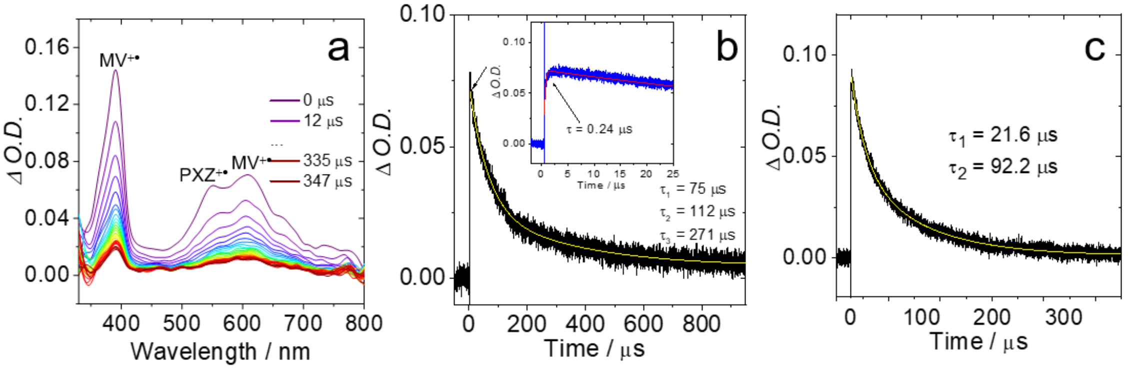

Figure S43. Nanosecond transient absorption spectra of (a) **S-NI-PXZ** with the addition of MV^{2+} (c = 3.0 × 10^{-4} M) in deaerated ACN. Decay traces of (b) $MV^{+\bullet}$ at 390 nm in deaerated ACN, (c) $PXZ^{+\bullet}$ at 390 nm in deaerated ACN. Excited with nanosecond pulsed laser. λ_{ex} = 355 nm. *c*[**S-NI-PXZ**] = 3.0 × 10^{-5} M. 25 °C.

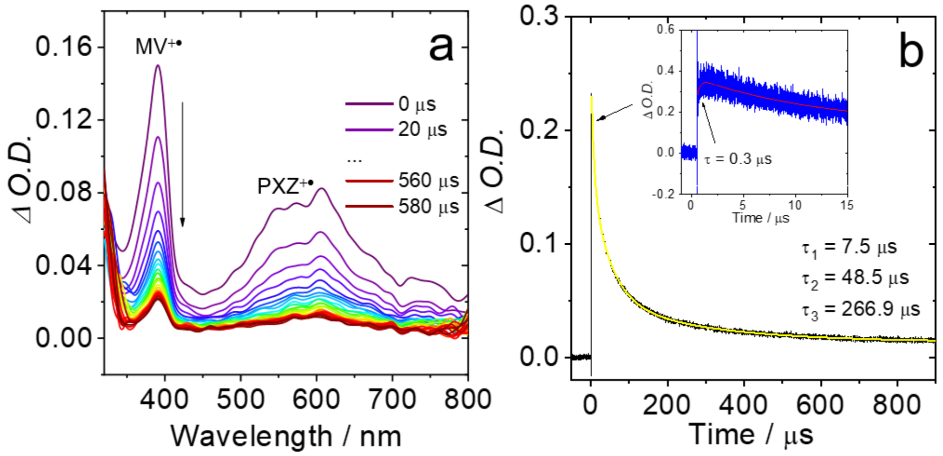

Figure S44. Nanosecond transient absorption spectra of (a) **NI-PXZ** with the addition of MV^{2+} (c = 3.0 × 10^{-4} M) in deaerated ACN. Decay traces of (b) $MV^{+\bullet}$ at 390 nm in deaerated ACN. Excited with nanosecond pulsed laser. λ_{ex} = 355 nm. *c*[**NI-PXZ**] = 3.0 × 10^{-5} M. 25 °C.

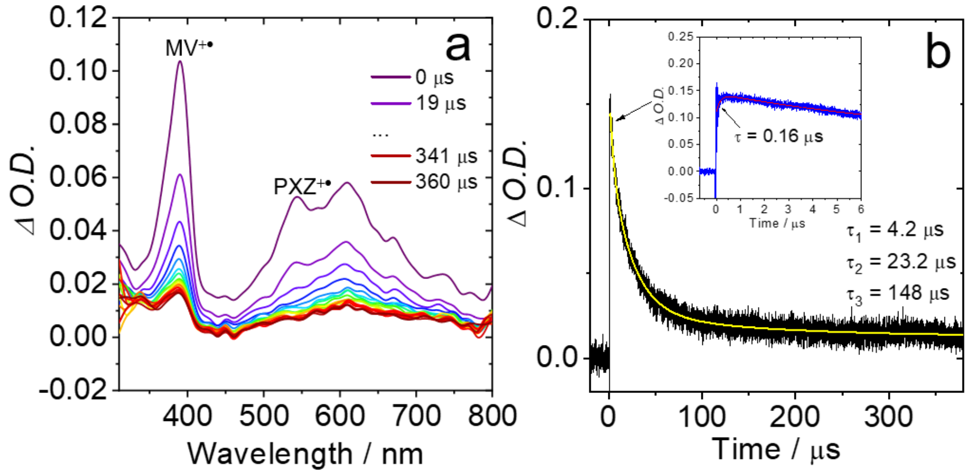

Figure S45. Nanosecond transient absorption spectra of (a) **DS-NI-PXZ** with the addition of MV^{2+} (c = 3.0 × 10^{-4} M) in deaerated ACN. Decay traces of (b) $MV^{+\bullet}$ at 390 nm in deaerated ACN. Excited with nanosecond pulsed laser. λ_{ex} = 355 nm. *c*[**DS-NI-PXZ**] = 3.0 × 10^{-5} M. 25 °C.

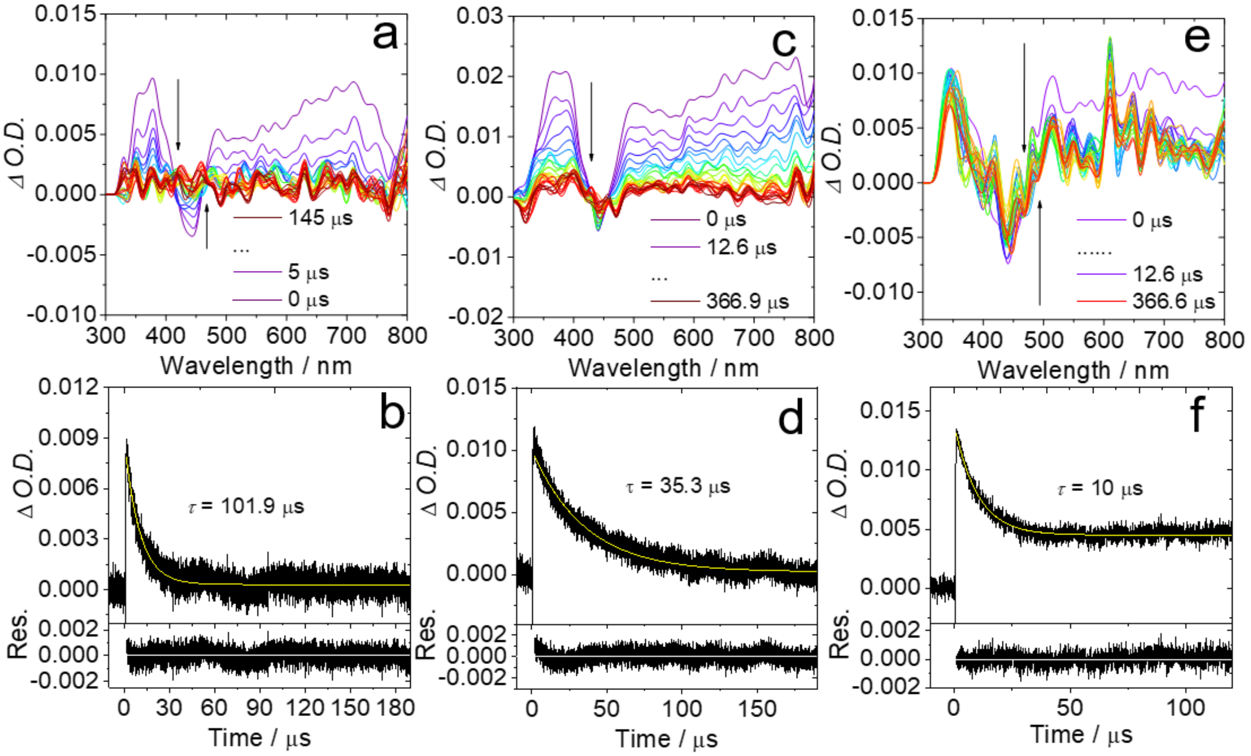

Figure S46. Nanosecond transient absorption spectra of **NI-DPA** in deaerated (a) *n*-HEX, (c) TOL and (e) ACN. The decay curves in deaerated (b) *n*-HEX, (d) TOL and (f) ACN at 500 nm after pulsed laser excitation at 355 nm, c = 3.0 × 10^{-5} M. 25 °C.

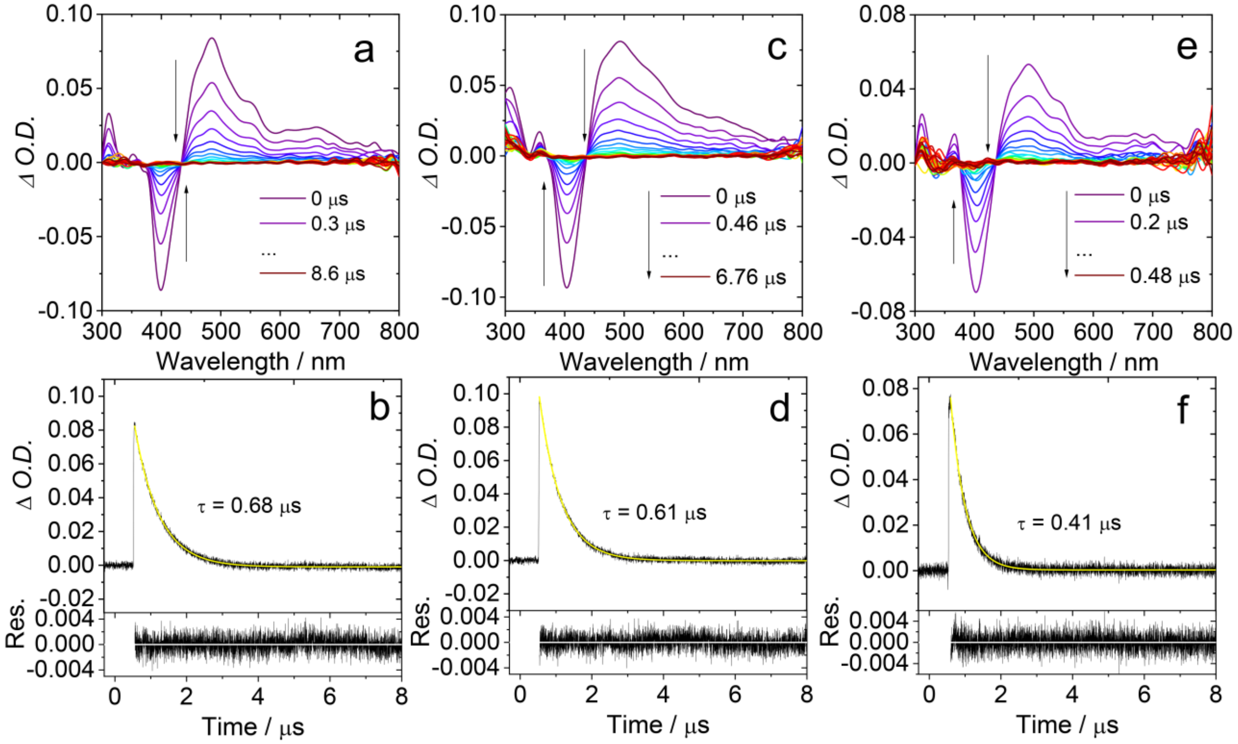

Figure S47. Nanosecond transient absorption spectra of **S-NI** in deaerated (a) HEX, (c) TOL and (e) ACN. The decay curves in deaerated (b) HEX, (d) TOL and (f) ACN at 500 nm after pulsed laser excitation at 355 nm, c = 2.0 × 10^{-5} M. 25 °C.

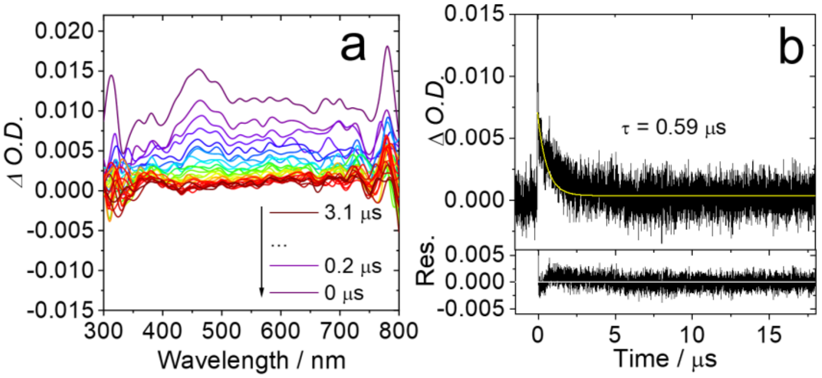

Figure S48. Nanosecond transient absorption spectra of **S-NI-PXZ** in deaerated (a) TOL. The decay curves in deaerated (b) TOL at 460 nm after pulsed laser excitation at 355 nm, c = 3.0 × 10^{-5} M. 25 °C.

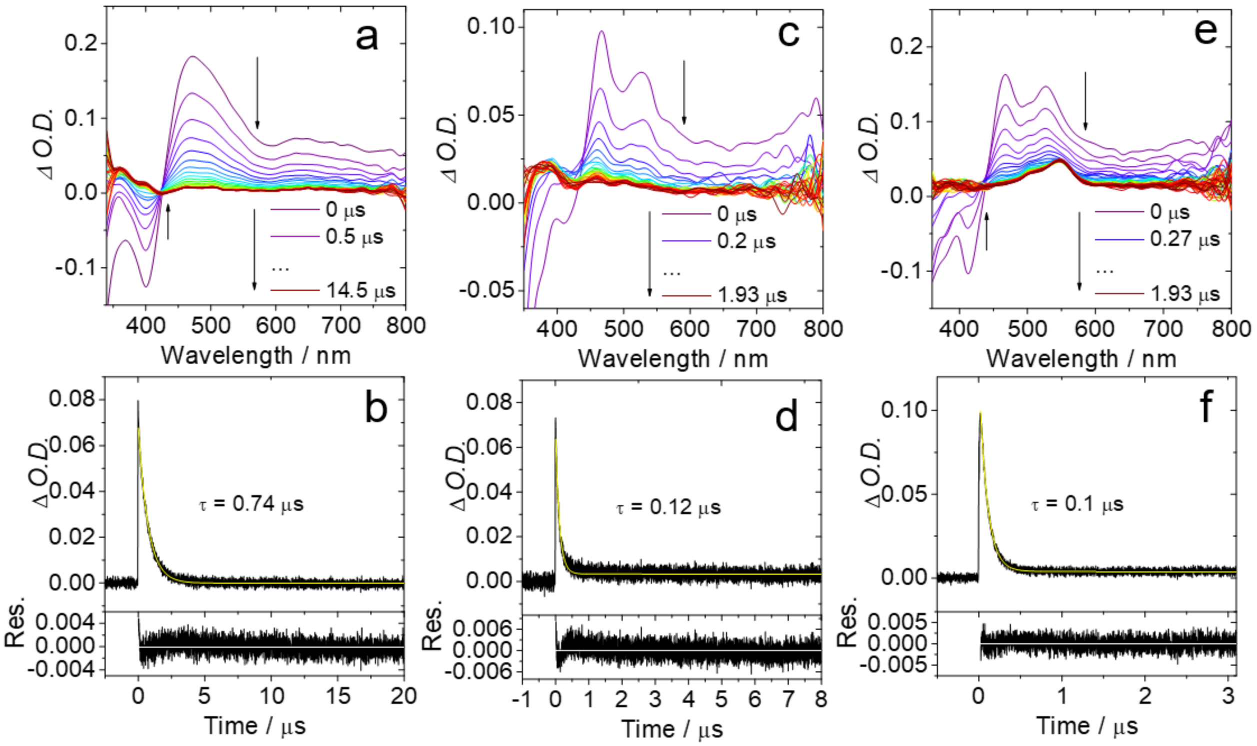

Figure S49. Nanosecond transient absorption spectra of **S-NI-PXZ** in deaerated (a) CHX (c) THF and (e) DCM. The decay curves in deaerated (b) CHX, (d) THF and (f) DCM at 460 nm after pulsed laser excitation at 355 nm, c = 3.0×10^{-5} M. 25 °C.

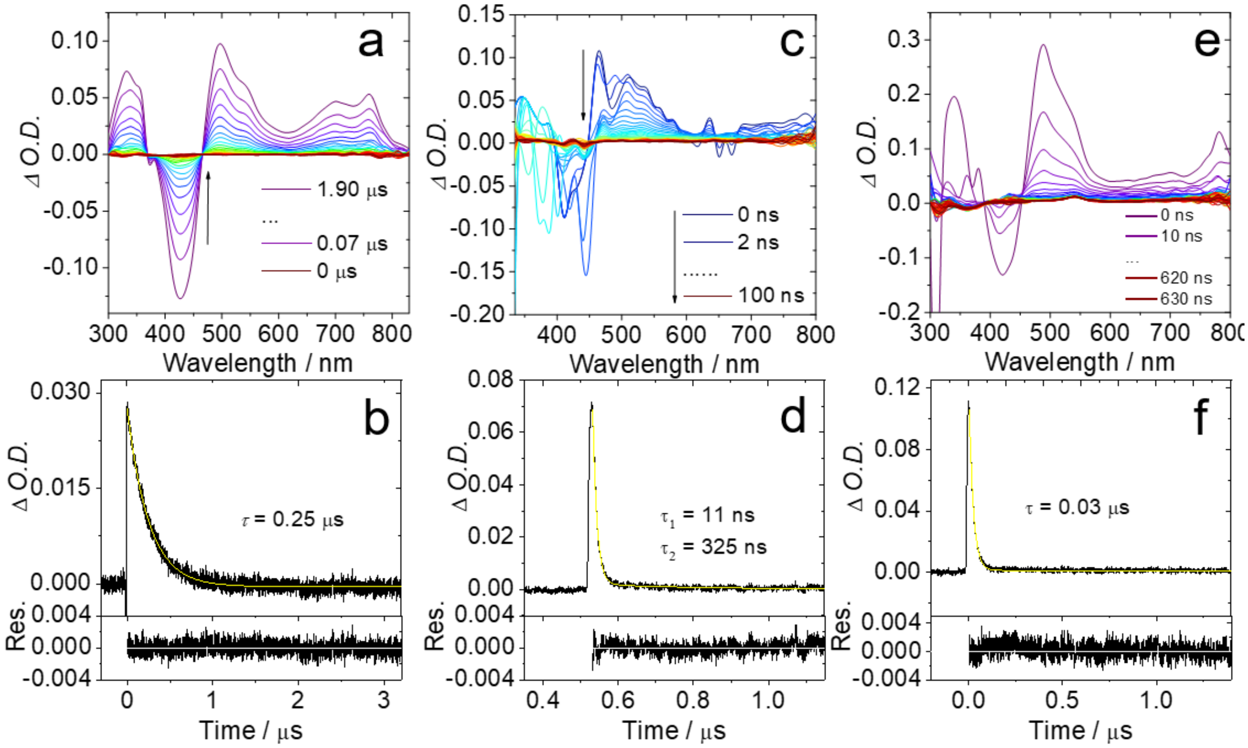

Figure S50. Nanosecond transient absorption spectra of **DS-NI-PXZ** in deaerated (a) *n*-HEX, (c) TOL and (e) ACN. The decay curves in deaerated (b) *n*-HEX, (d) TOL and (f) ACN at 530 nm after pulsed laser excitation at 355 nm, c = 3.0 × 10^{-5} M. 25 ℃.

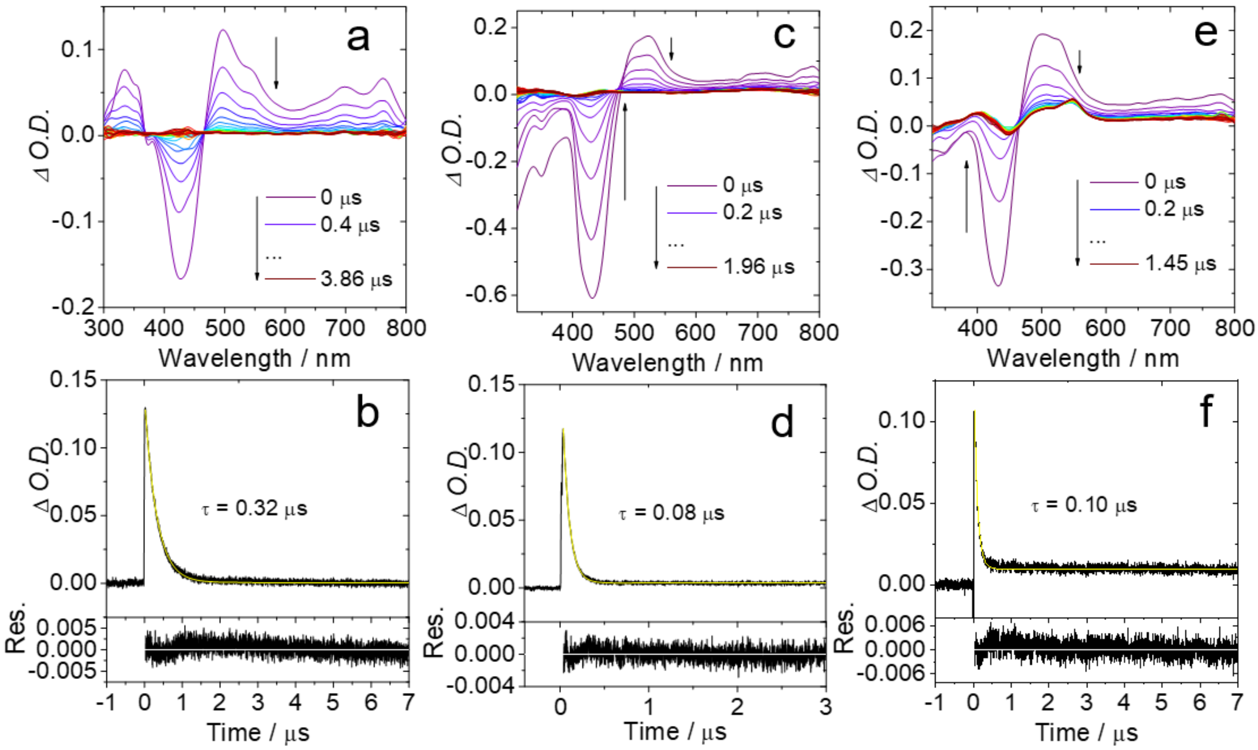

Figure S51. Nanosecond transient absorption spectra of **DS-NI-PXZ** in deaerated (a) CHX, (c) THF and (e) DCM. The decay curves in deaerated (b) CHX, (d) THF and (f) DCM at 530 nm after pulsed laser excitation at 355 nm, c = 3.0 × 10^{-5} M. 25 ℃.

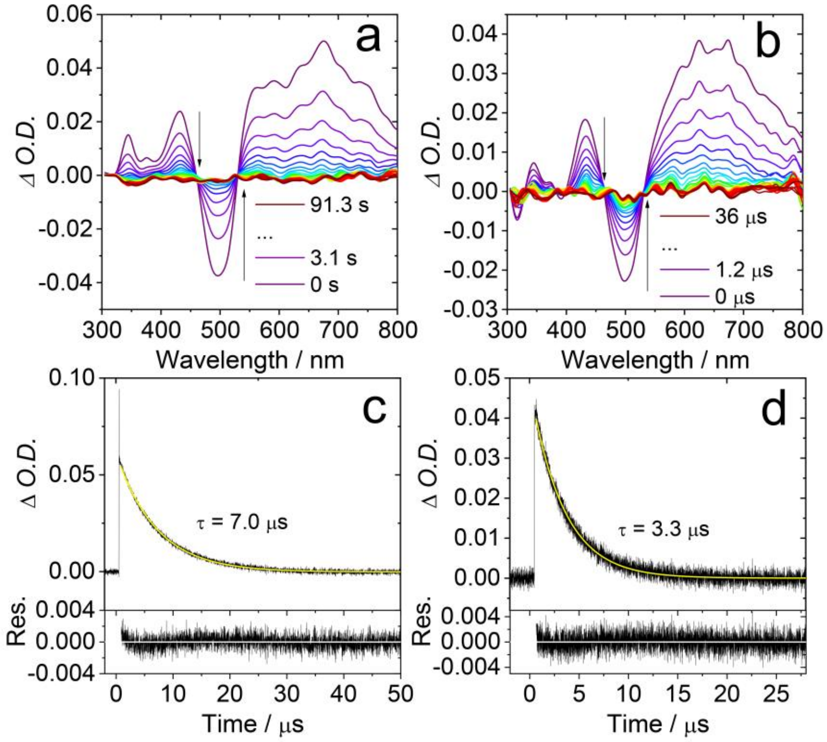

Figure S52. Nanosecond transient absorption spectra of **S-NI-DPA** in deaerated (a) TOL and (b) ACN. The decay curves in deaerated (c) TOL and (d) ACN at 675 nm after pulsed laser excitation at 500 nm, c = 3.0 × 10^{-5} M. 25 °C.

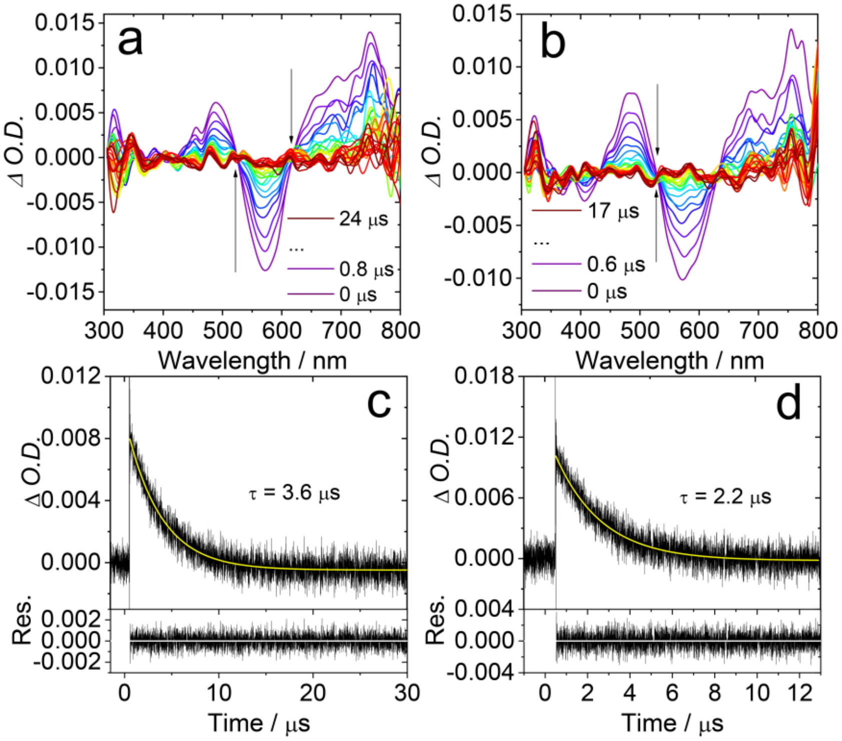

Figure S53. Nanosecond transient absorption spectra of **DS-NI-DPA** in deaerated (a) TOL and (b) ACN. The decay curves in deaerated (c) TOL and (d) ACN at 495 nm after pulsed laser excitation at 550 nm, c = 3.0×10^{-5} M. 25 °C.

10. Theoretical Calculations

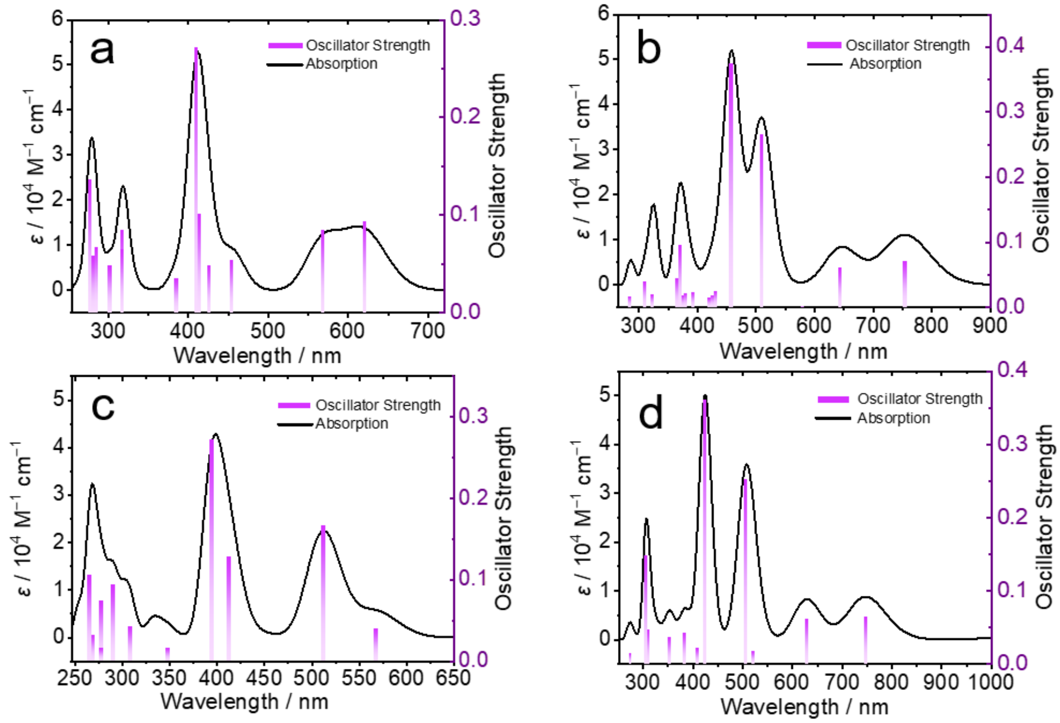

Figure S54. The calculated transient absorption spectra of (a) $[DS\text{-}NI]^{-\bullet}$, (b) $[PXZ]^{+\bullet}$ of **DS-NI-PXZ** and (c) $[S\text{-}NI]^{-\bullet}$, (d) $[PXZ]^{+\bullet}$ of **S-NI-PXZ**, i.e. the $S_0 \rightarrow S_n$ transitions. Calculated by DFT [CAM-B3LYP/6-31G(d)] in vacuum using Gaussian 16, based on the optimized ground state geometries.

Table S3. Main Transition Orbitals; Electronic Excitation Energies (eV) and Oscillator Strengths *f* of Excited States of **DS-NI-PXZ**, **S-NI-PXZ**, **DS-NI-DPA** and **S-NI-DPA**; Calculated at the CAM-B3LYP/6-31G(d) Level with Gaussian 16. No solvent was used in calculations.

	State	Energy [eV/nm][a]	Composition[b]	*f*[c]	Character
**DS-NI-PXZ**	S_1	1.15/1080	H → L	0.0000	CT
	S_4	2.60/476	H–2 → L	0.0001	CT
	S_5	2.86/432	H–3 → L	0.3333	LE
	T_1	1.35/919	H–2 → L	0.0000	CT
	T_2	1.71/725	H–1 → L	0.0000	LE
	T_3	2.27/546	H → L	0.0000	CT
**S-NI-PXZ**	S_1	1.39/889	H → L	0.0000	CT
	S_4	3.15/392	H–3 → L	0.3033	LE
	T_1	1.38/894	H → L	0.0000	CT
	T_2	1.83/676	H–3 → L	0.0000	LE
	T_3	1. 90/653	H–1 → L	0.0000	LE
**DS-NI-DPA**	S_1	1.85/668	H–1 → L	0.0055	LE
	S_2	2.16/572	H → L	0.2068	CT
	S_3	2.29/540	H–2 → L	0.0753	LE
	T_1	1.44/857	H → L	0.0000	CT
	T_2	1.64/753	H–1 → L	0.0000	LE
	T_3	1.90/649	H–2 → L	0.0000	LE
**S-NI-DPA**	S_1	2.19/563	H–1 → L	0.0013	LE
	S_2	2.38/519	H → L	0.2131	CT
	S_3	3.27/378	H–2 → L	0.1763	LE
	T_1	1.74/709	H → L	0.0000	CT
	T_2	1.94/637	H–1 → L	0.0000	LE
	T_3	2.28/543	H–2 →L	0.0000	LE

[a] Only the selected low-lying excited states are presented. [b] Only the main configurations are presented. [c] Oscillator strengths.

Table S4. Relevant matrix elements for the spin-orbit coupling, SOC, $\langle T|H_{SO}|S\rangle_i$ (Re, Im and with *i=X,Y,Z*), between the triplet T_1 and the ground state S_0 (triplet depopulation). The sum of the squares of the SOC values are also displayed as k_{tot}.[6,7]

	$\langle T\|H_{SO}\|S\rangle_X$[a]	$\langle T\|H_{SO}\|S\rangle_Y$[a]	$\langle T\|H_{SO}\|S\rangle_Z$[a]	k_{tot}[b]
**NI**	0, 1.42	0, –2.74	0, –0.28	9.60
**S-NI**	0, –54.29	0, –117.81	0, –64.15	20941.82
**DS-NI**	0, –71.6	0, –115.99	0, –0.05	18580.24
**S-NI-PXZ**	0, –24.58	0, –6.97	0, –0.03	652.76
**DS-NI-PXZ**	0, –83.77	0, 103.60	0, –24.21	18336.50

[a] cm^{-1}; [b] cm^{-2};

Table S5. Relevant matrix elements for the triplet population via spin-orbit coupling, SOC, $\langle T|H_{SO}|S\rangle_i$ (Re, Im and with *i=X,Y,Z*) for low-energy excited singlets S_n (the energy difference S_n – S_1 is reported in parenthesis) ; because of the $\delta(E_T - E_S)$ factor, only triplets with a small $|E_T - E_S|$ energy difference were considered. The sum of the square of the SOC values are displayed as k_{tot}.[6,7]

	S_n ($\Delta E(S_n - S_1)$[a])	T_m	$\|E_T - E_S\|$[a]	$\langle T\|H_{SO}\|S\rangle_X$[a]	$\langle T\|H_{SO}\|S\rangle_Y$[a]	$\langle T\|H_{SO}\|S\rangle_Z$[a]	k_{tot}[b]
**NI**	S_2 (+1485)	T_4	1432	0, 6.85	0, –13.42	0, –1.50	229
**S-NI**	S_1 (0)	T_2	1408	0, 30.72	0, 55.24	0, 28.36	4793
**DS-NI**	S_1 (0)	T_3	870	0, 0	0, 0	0, –49.8	2500
**S-NI-PXZ**	S_1 (0)	T_1	127	0, 0	0, 0	0, 0.02	2×10^{-4}
	S_2 (+3642)	T_3	628	0, 89.29	0, 20.02	0, 0.10	8321
**DS-NI-PXZ**	S_2 (+921)	T_5	1555	0, 24.58	0, –31.99	0, –1.25	1628

[a] cm^{-1}; [b] cm^{-2}

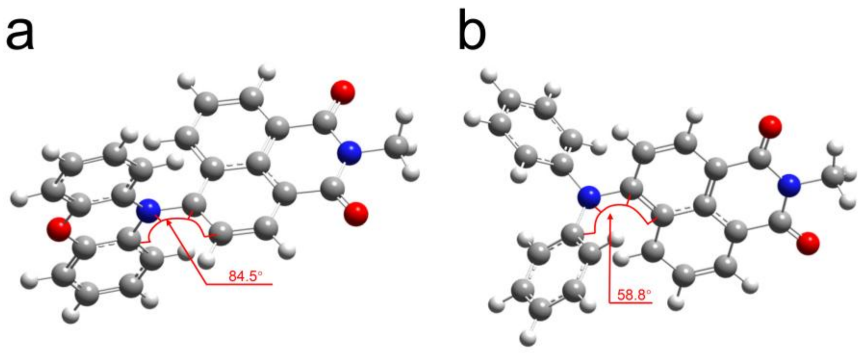

Figure S55. Dihedral angle optimized planes of (a) **NI-PXZ** and (b) **NI-DPA**, based on the optimized ground-state geometries. Calculated with the CAM-B3LYP/6-31G(d) level of Gaussian 16.

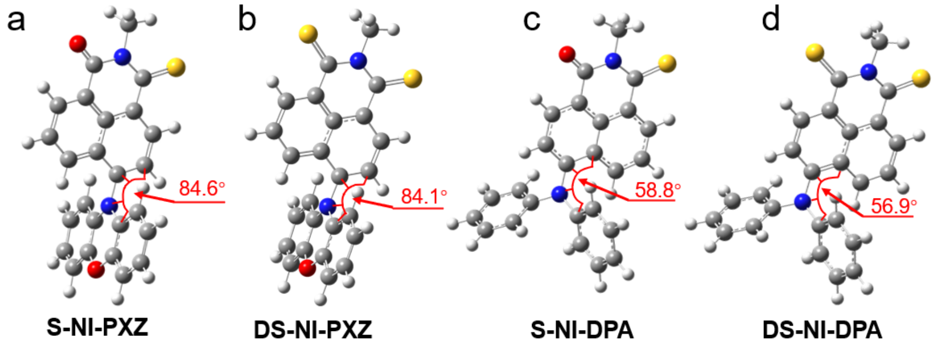

Figure S56. Dihedral angles in the ground state optimized geometry of (a) **S-NI-PXZ**, (b) **DS-NI-PXZ**, (c) **S-NI-DPA** and (d) **DS-NI-DPA**, of the ground-state. Calculated at the CAM-B3LYP/6-31G(d) level using theory of Gaussian 16. Alkyl chains are simplified as methyl groups to reduce computation time.

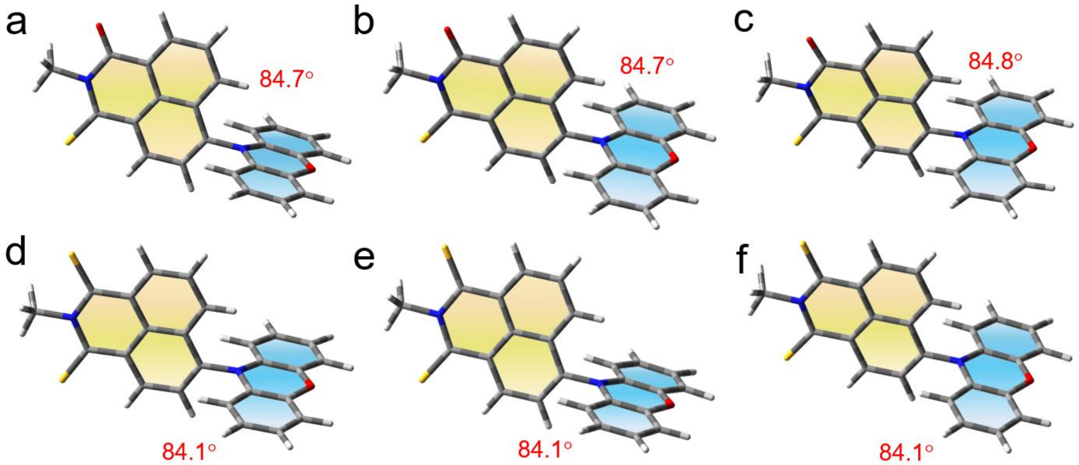

Figure S57.The optimized ground state geometry of the molecular structures of **S-NI-PXZ** in (a) CHX (b) TOL, (c) ACN and **DS-NI-PXZ** in (d) CHX (e) TOL, (f) ACN (Model: IEFPCM). The dihedral angle between the thionated NI and PXZ moieties is marked in red. Calculated at CAM-B3LYP/6-31G (d) level with Gaussian 16.

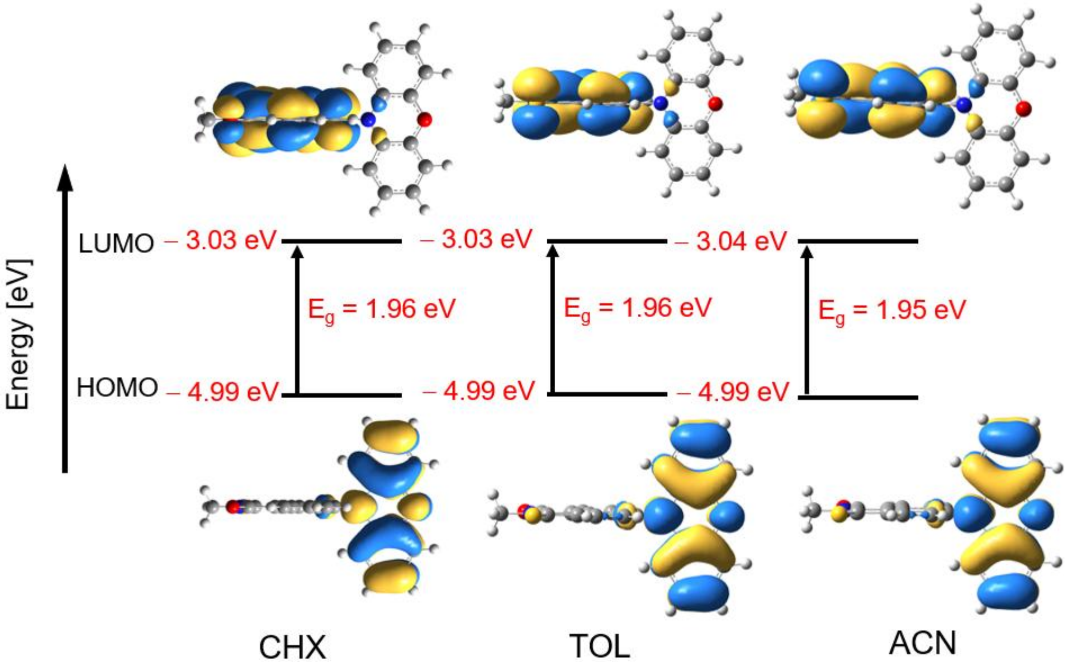

Figure S58. The Molecular orbitals and energies of the selected frontier orbitals (isovalue = 0.02) of **S-NI-PXZ**, based on the optimized ground state and the excited state geometries in CHX, TOL and ACN (Model: IEFPCM). Calculation was performed by DFT method at the CAM-B3LYP/6-31G(d) level with Gaussian 16. Alkyl chains are simplified as methyl group to reduce computation time.

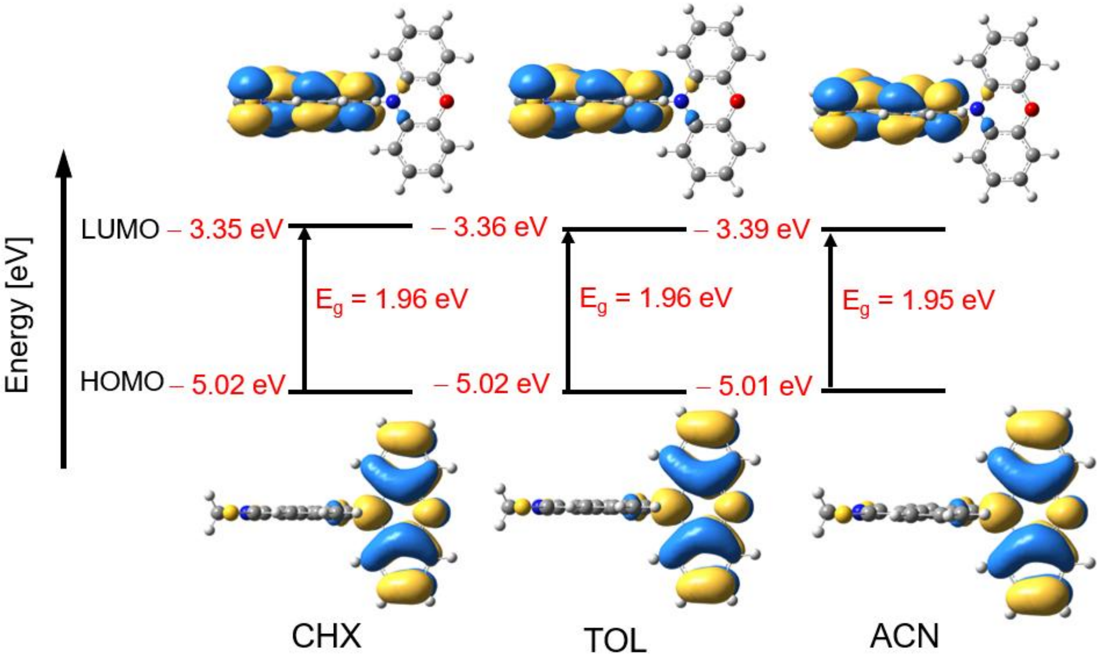

Figure S59. The Molecular orbitals and energies of the selected frontier orbitals (isovalue = 0.02) of **DS-NI-PXZ**, based on the optimized ground state and the excited state geometries in CHX, TOL and ACN (Model: IEFPCM). Calculation was performed by DFT method at the CAM-B3LYP/6-31G(d) level with Gaussian 16. Alkyl chains are simplified as methyl group to reduce computation time.

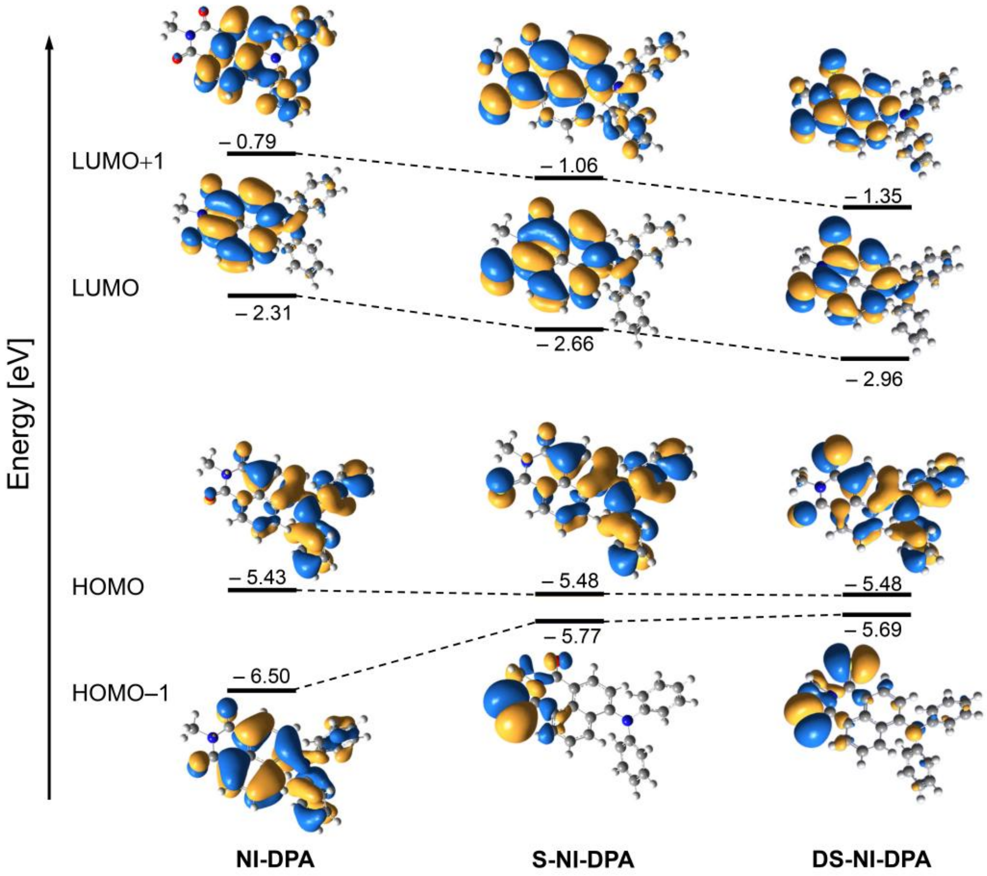

Figure S60. The Molecular orbitals and energies of the selected frontier orbitals (isovalue = 0.02) of **NI-DPA**, **S-NI-DPA** and **DS-NI-DPA**, based on the optimized ground state and the excited state geometries, unused solvent, respectively. Calculation was performed by DFT method at the CAM-B3LYP/6-31G(d) level with Gaussian 16. Alkyl chains are simplified as methyl group to reduce computation time.

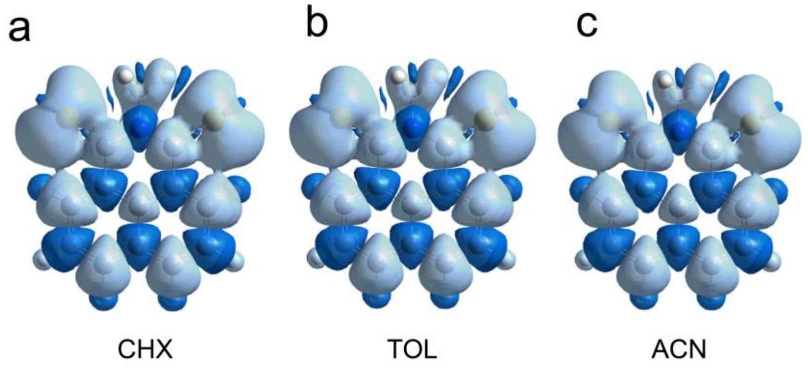

Figure S61. Spin density surfaces and spin density distributions (isovalue = 0.0004) of the triplet state of **DS-NI** at the optimized triplet state geometries in (a) CHX, (b) TOL and (c) ACN. Calculated at the CAM-B3LYP/6-31G(d) level with Gaussian 16.

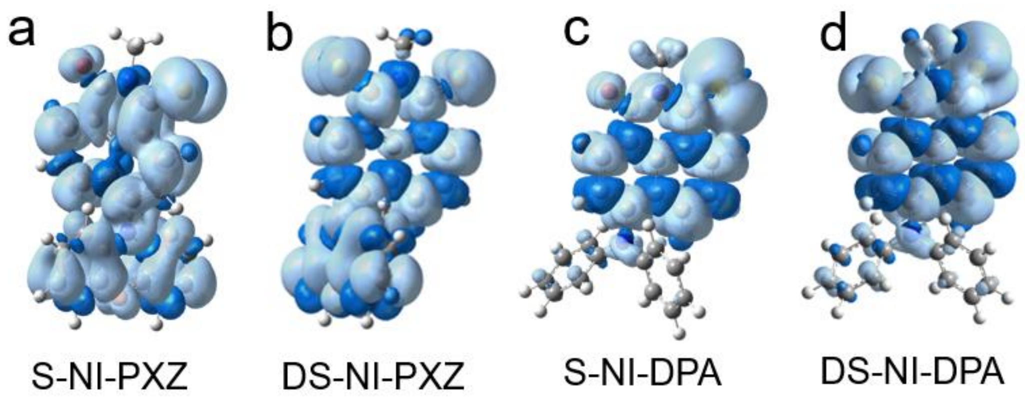

Figure S62. Spin density surfaces of the triplet state of (a) **S-NI-PXZ**, (b) **DS-NI-PXZ**, (c) **S-NI-DPA** and (d) **DS-NI-DPA** in TOL. Calculated at the CAM-B3LYP/6-31G(d) level with Gaussian 16 (isovalue = 0.0004).

Scheme S1. Photophysical processes of **DS-NI-PXZ** in different solvents.

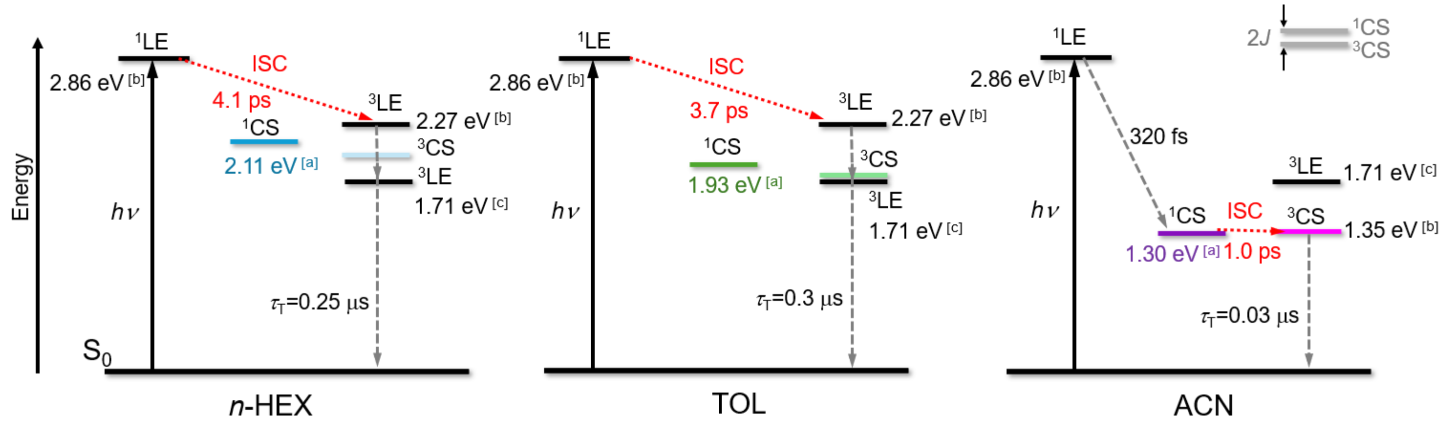

[a] The energy of CS states are approximated from electrochemical data. [b]The energy of the singlet states and the triplet states are from calculation at TDDFT CAM-B3LYP/6-31G(d) level with Gaussian 16. [c]The triplet localized excited state energy is estimated by the vibrational 00 transition of $T_1 \rightarrow S_0$ based on phosphorescence spectra. *J* is the electronic exchange energy, and the energy level difference between ^{1}CS and ^{3}CS is 2*J*. The number of the superscript designates spin multiplicity.

11. References

[1] X. Xiao, T. Mu, A. A. Sukhanov, Y. Zhou, P. Yu, F. Yu, A. Elmali, J. Zhao, A. Karatay, V. K. Voronkova, *Phys. Chem. Chem. Phys.* **2023**, *25*, 31667–31682.

[2] J. J. Snellenburg, S. Laptenok, R. Seger, K. M. Mullen, I. H. M. van Stokkum, *J. Stat. Softw.* **2012**, *49*, 1–22.

[3] S. Stoll, A. Schweiger, *J. Magn. Reson.* **2006**, *178*, 42–55.

[4] R. Strzelczyk, S. Ciuti, A. Carella, M. Bortolus, L. Franco, A. Zoleo, M. Ruzzi, A. Toffoletti, M. Di Valentin, D. Carbonera, A. Barbon, *Appl. Magn. Reson.* **2024**, *55*, 1515–1537.

[5] M. J. Frisch, G. Trucks, H. B. Schlegel, G. E. Scuseria, M. A. Robb, J. Cheeseman, i. G. Scalmani, V. Barone, G. A. Petersson, H. Nakatsuji, Gaussian 16, Revision C. 01; Gaussian, Inc.: Wallingford CT, **2016**.

[6] H. Yersin, R. Czerwieniec, U. Monkowius, R. Ramazanov, R. Valiev, M. Z. Shafikov, W. M. Kwok, C. Ma, *Coord. Chem. Rev.* **2023**, *478*, 214975.

[7] Z. Wang, T. Antonio, Y. Hou, J. Zhao, B. Antonio, D. Bernhard, *Chem. Sci.* **2021***, 12,* 2829–2840.
